# Can we identify people with Alzheimer’s disease from examination of the eye? A bidirectional Mendelian randomization (MR) study

**DOI:** 10.1016/j.tjpad.2026.100635

**Published:** 2026-07-10

**Authors:** Humayun Kiser, Ashley Budu-Aggrey, Jessica N. Cooke Bailey, Ana Villaplana-Velasco, Miguel O. Bernabeu, Xiaofan Jiang, Christopher G. Owen, Jonathan L. Haines, Louis R. Pasquale, Stuart MacGregor, Xiaoyi Raymond Gao, Janey L. Wiggs, Chen Jiang, Hélène Choquet, NEIGHBORHOOD consortium, International Glaucoma Genetics Consortium, UK Biobank Eye and Vision Consortium, George Davey Smith, Patrick G. Kehoe, Neil M. Davies, Aimee L. Hanson, Emma L. Anderson, Denize Atan

**Affiliations:** a Bristol Medical School, Translational Health Sciences, University of Bristol, Bristol, UK; b Department of Statistics, Comilla University, Cumilla, Bangladesh; c Medical Research Council (MRC) Integrative Epidemiology Unit, University of Bristol, Bristol, UK; d Bristol Medical School, Population Health Sciences, University of Bristol, Bristol, UK; e Center for Health Disparities, Department of Pharmacology & Toxicology, Brody School of Medicine, East Carolina University, Greenville, NC, 27834, USA; f Baillie Gifford Pandemic Science Hub, Centre for Inflammation Research, The Queen’s Medical Research Institute, University of Edinburgh, Edinburgh, UK; g Centre for Medical Informatics, Usher Institute, The University of Edinburgh, Edinburgh, Scotland, UK; h The Bayes Centre, The University of Edinburgh, Edinburgh, Scotland, UK; i UCL Institute of Ophthalmology, London. UK; j UCL Great Ormond Street Institute of Child Health, University College London, London, UK; k Population Health Research Institute, City St. George’s, University of London, London, UK; l Department of Population and Quantitative Health Sciences, School of Medicine, Case Western Reserve University, Cleveland, OH, USA; m Cleveland Institute for Computational Biology, Case Western Reserve University, Cleveland, OH, USA; n Department of Ophthalmology, Icahn School of Medicine at Mount Sinai, New York, NY USA; o QIMR Berghofer Medical Research Institute, Brisbane, Queensland, Australia; p Departments of Ophthalmology and Visual Science and Biomedical Informatics, Division of Human Genetics, The Ohio State University, Columbus, OH 43212, USA; q Department of Ophthalmology, Massachusetts Eye and Ear Infirmary, Harvard Medical School, Boston, MA, USA; r Division of Research, Kaiser Permanente Northern California, Pleasanton, CA, USA; s Department of Health Systems Science Kaiser Permanente Bernard J. Tyson School of Medicine, Pasadena, CA, USA; t Department of Public Health and Nursing, NTNU, Norwegian University of Science and Technology, 7034 Trondheim, Norway; u Department of Statistical Science, University College London, London, UK; v Division of Psychiatry, University College London, London, UK; w School of Neuroscience and Psychology, University of Bristol, Bristol, UK; x Bristol Eye Hospital, University Hospitals Bristol & Weston NHS Foundation Trust, Bristol, UK

**Keywords:** Alzheimer’s disease, Vasculature, Retina, Optic disc, Mendelian randomization

## Abstract

**Background:**

Neurodegeneration in Alzheimer’s disease (AD) is thought to be driven by amyloid-beta and tau deposition in the cerebral vasculature and brain. As the eye is an extension of the central nervous system, this study aimed to determine which neurovascular and neuroretinal changes in the eye are caused by AD rather than associations of the disease.

**Methods:**

Bidirectional two-sample univariable and multivariable Mendelian randomization (MR) methods were applied. Instrumental variables were derived from genome-wide association studies (GWAS) of AD and the following ocular features: thickness measurements of central macula (MT), retinal nerve fibre layer (mRNFL), ganglion cell-inner plexiform layer (mGCIPL), outer nuclear layer (ONL), inner segment layer (IS), and outer segment (OS) from macular region OCT scans; arteriolar tortuosity (AT), venular tortuosity (VT), venular width (VW), fractal dimension (FD), vertical cup-to-disc ratio (VCDR), optic cup area (OCA), and optic disc area (ODA) derived from other imaging methods.

**Results:**

There was strong evidence that genetic liability to AD affected the retinal vasculature by specifically increasing AT (β = 0.007;95%CI=0.002,0.011;p-value=0.005) in UK Biobank participants (n=52,798). AD may influence the mRNFL (β=-0.047,95%CI=-0.119,0.023,p-value=0.18) and mGCIPL (β=-0.061;95%CI=-0.14,0.025,p-value=0.16) of the inner retina and OS layer (β = 0.044;95%CI=-0.0001,0.08;p-value=0.05) but the evidence was weak. Multivariable MR analysis showed that a causal relationship between optic disc area and AD (OR=0.76;95%CI=0.62,0.93,p-value=0.009) was probably mediated by refractive error.

**Conclusion:**

Early cerebrovascular signs of AD may be detected by examination of the eye. Further investigation is required to determine the clinical utility of eye screening for dementia.

## Highlights

• Genetic risk of Alzheimer’s disease (AD) causes increased retinal arteriolar tortuosity.
• AD may also cause inner retinal degeneration, but the evidence is weak.
• Odds of AD reduced by 24% for each standard deviation increase in optic disc area.
• The relationship between AD and optic disc area was mediated by refractive error.

## Graphical abstract

**Figure:**
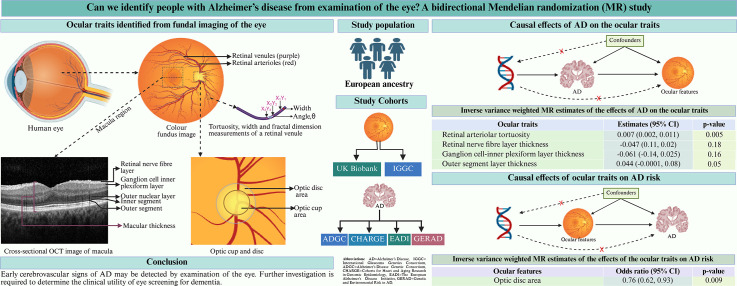

## 1 Introduction

Early identification of people with dementia has become a global research priority. By around 2050, it’s predicted that dementia will affect 152.8 million people, with an annual healthcare cost of >USD$1.4 trillion [1]. Late-onset Alzheimer’s disease (AD) is the commonest type, affecting 60–80% of people with dementia. Although new therapies are emerging to slow or delay the cognitive decline of people with AD, they do not stop progression or reverse pathology, and they can have severe side effects [2]. Hence, identifying early signs of the disease may provide opportunities to start interventions when they are more likely to have greater benefits.

AD pathology is characterised by neurodegenerative changes that are thought to be driven by the accumulation of intracellular hyperphosphorylated tau protein and neurofibrillary tangles within neurons, and extracellular amyloid-beta protein deposition in brain tissue and within cerebral blood vessel walls. As the eye is an extension of the central nervous system (CNS), human studies and mouse models of AD have shown the retina and optic nerve are also affected by amyloid-beta deposition and neurodegeneration, particularly thinning of the inner retinal layers: the retinal nerve fibre layer (RNFL) and ganglion cell inner plexiform layer (GCIPL) [3]. Similarly, inner retinal degeneration is associated with brain MRI markers of neurodegeneration, cognitive decline and future dementia diagnosis [4,5]. Moreover, amyloid microangiopathy can affect retinal and choroidal vasculature as well as cerebral blood flow, since the eye and brain share a common blood supply from the internal carotid artery [3]. Hence, abnormalities of visual function are common in AD patients [3]. As the retina and optic nerve are the only parts of the CNS that can be directly visualized by clinical examination and non-invasive imaging of the eye, it’s possible that early signs of dementia could be detected from a simple eye test.

Previous observational studies have explored whether an eye examination, e.g., to detect signs of inner retinal neurodegeneration, could be a more sensitive and cost-effective way to identify people with AD much earlier than, for example, MRI imaging; however, a recent systematic review has highlighted the heterogeneity of past studies and limitations in their study design that could lead to bias [6]. Furthermore, observational studies cannot determine the aetiology or causal relationships between AD and neurodegenerative changes in the eye. Past large-scale genome-wide association studies (GWAS) (e.g., *Kunkle* et al.; n = 21,982 AD cases) [7] have identified genetic polymorphisms associated with AD, among which *apolipoprotein E* (*APOE*) variants are the strongest genetic risk factors for late-onset AD [[7], [8], [9], [10]]. Other large GWAS have identified variants associated with the thickness of the retina and its layers (e.g., *Currant* et al.; n = 31,434) [11], optic disc morphology (e.g., *Springelkamp* et al.; n = 22,484) [12] and the retinal vasculature (e.g., *Jiang* et al.; n = 52,798) [13]. These studies provided the opportunity to use an alternative approach, Mendelian Randomization (MR), to investigate the causal effects of AD on these ocular traits with unprecedented statistical power (Supplementary Figures S1 and S2). MR is less vulnerable to bias from confounding and reverse causation than observational epidemiology studies, and can provide greater insight into the neurovascular and neuroretinal changes in the eye that are directly caused by AD rather associations with the disease.

## 2 Methods

### 2.1 Study design

MR is a statistical method that uses genetic variants (including single-nucleotide polymorphisms or SNPs) that are strongly associated with an exposure in GWAS as instrumental variables to estimate the causal effects of the exposure on the trait of interest. In this two-sample MR study, genetic variant (instrument) associations with the exposures and instrument associations with the outcomes were obtained from GWAS of non-overlapping samples to estimate the causal effects of genetic liability to AD on each ocular trait. In separate MR analyses, the causal effects of genetic liability to each ocular trait on AD risk were estimated in the other direction. These bidirectional MR analyses were used to identify the directionality of any causal relationships (Supplementary Methods, Supplementary Figure S2) [14].

All analyses were performed using R version 3.6.1 (www.r-project.org), and two-sample MR analyses were applied using the Wald ratios method in the TwoSampleMR package (version 0.6.4). The code for these MR analyses is provided here: https://github.com/HuKiser/Retinal_features_AD_MR.

### 2.2 Study cohorts

GWAS summary data for late-onset AD were obtained through the IEU Open GWAS project [15]; likewise, summary data for the ocular traits were obtained from published GWAS (Supplementary Figure S1) [[11], [12], [13],[16], [17], [18]]. Ethical approval was granted for each GWAS. Further details about participant ascertainment, recruitment, genotyping, and imaging/phenotyping procedures are available in Supplementary Methods and Supplementary Table S1.

#### 2.2.1 Genetic instruments for exposure and outcome

Genetic instruments for each exposure and outcome were selected following the same pipeline (Supplementary Figures S3-S5). Only instruments with an F-statistic>10 were retained in univariable MR analyses to minimise weak instrument bias (Supplementary Tables S2-S17).

### 2.3 Statistical analyses

The effect of each exposure on the outcome was estimated using inverse-variance weighted (IVW) regression; the results were transformed into SD units per doubling of odds of genetic liability to AD risk to compare the magnitude of the effects across different ocular traits [19]. For analyses in the reverse direction, causal estimates were expressed in odds ratio (OR) per SD increase in each ocular trait.

### 2.4 Sensitivity analyses

Alternative MR methods (MR-Egger, weighted median, weighted mode) were used in sensitivity analyses to test for horizontal pleiotropy. Horizontal pleiotropy can occur when genetic variants affect the outcome via pathways independent of the exposure of interest. Additional details about the core assumptions of MR and the differences between MR methods are provided in Supplementary Methods. The Q-statistic was used to assess the heterogeneity in causal effect estimates across the genetic variants used as instruments in each analysis; this can indicate the presence of pleiotropy. The Steiger test was applied to determine whether any genetic instruments explained more variance in the outcome than the exposure; however, Steiger filtering was only used in sensitivity analyses because Steiger filtering can infer the wrong direction of causality when applied to binary outcomes and parameters vulnerable to measurement error. Additionally, multivariable MR (MVMR) was used to investigate the effects of optic disc area (ODA) on AD to determine whether they were mediated by refractive error (RE) and/or axial length (AL).

Funnel plots were used to look for directional pleiotropy in each MR analysis visually. Leave-one-out analyses were applied to look for dominating SNPs in the estimates. Finally, to avoid winner’s curse, the MR analyses were replicated using summary data from three other GWAS of AD conducted by Lambert et al. [8] (17,008 cases, 37,154 controls), Jansen et al. [9] (71,880 cases, 383,378 controls) and Bellenguez et al. [10] (111,326 cases, 677,663 controls). The studies by Kunkle et al. [7] and Lambert et al. [8] were based on clinically confirmed AD cases. Individuals who were considered at higher risk of AD, based on their parental history, were included as AD proxy cases in the studies by Jansen et al. [9] and Bellenguez et al. [10].

### 2.5 Genetic correlation between ocular traits

Linkage disequilibrium score regression (LDSC) was used to estimate the genetic correlations between the ocular traits and AD based on the GWAS summary data using ldsc software (https://github.com/bulik/ldsc/, v1.0.1) and LD scores from the 1000 Genomes reference database [20]. Macular thickness (MT) was not included in this analysis because the full GWAS summary data weren’t available.

## 3 Results

### 3.1 AD causes greater retinal arteriolar tortuosity

Retinal vascular changes can be caused by several ischaemic risk factors for cardiovascular disease, stroke and dementia [21]; hence, the causality and direction of the causal relationships between AD and the retinal vascular traits in this study were assessed in both directions in separate MR analyses.

**Fig. 1:**
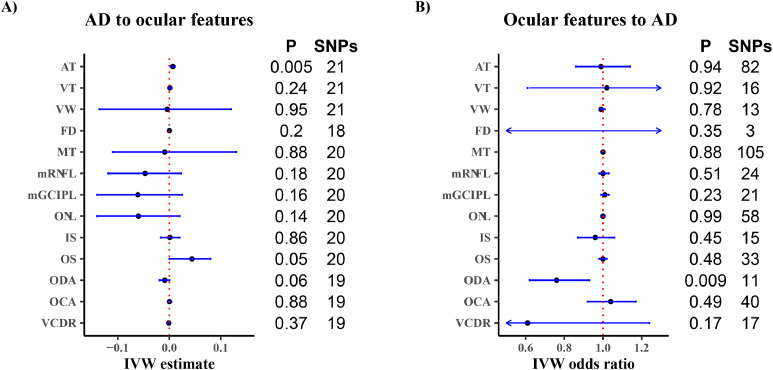
Forest plots summarizing the bi-directional causal relationships between AD and the ocular traits identified by eye examination in this study. (A) Inverse variance weighted (IVW) estimates of the causal effects of AD (in standard deviations) on the ocular traits; (B) Inverse variance weighted (IVW) odds ratio estimates of causal effects of the ocular traits (in standard deviations) on AD risk. The number of valid genetic variants (SNPs) used as instrument variables in each analysis and significance level (p-value) are shown in the figure. **Abbreviations:** MT= Macular Thickness, mRNFL= macular Retinal Nerve Fibre Layer, mGCIPL= macular Ganglion Cell Inner Plexiform Layer, ONL=outer nuclear layer, IS=inner segment layer thickness, OS=outer segment layer, AT=Arteriolar Tortuosity, VT=Venular Tortuosity, VW=Venular Width, and FD=retinal vessel fractal dimension, ODA=optic disc area, OCA=optic cup area, VCDR=vertical cup disc ratio.

The results of IVW regression provided strong evidence that increased retinal arteriolar tortuosity (AT) was caused by genetic liability to AD (IVW regression β = 0.007, 95%CI=0.002,0.011,p-value=0.005)(Table 1, Fig. 1A, Supplementary Figure S6A). In sensitivity analyses using alternate MR methods, the causal effects of AD on arteriolar tortuosity were consistent in size and direction (Table 1, Supplementary Figure S6A), and there was little evidence of horizontal pleiotropy (MR-Egger intercept=0.001; 95%CI=−0.001,0.003; p-value=0.38)(Table 1).

**Table 1:** The causal effects of Alzheimer's disease on retinal vascular morphology based on univariable Mendelian randomization.

Analysis	Estimate (95% CI)	P-value
**AD genetic risk – AT**		
IVW	0.007 (0.002, 0.011)	**0.005**
MR-Egger	0.005 (−0.0006, 0.011)	0.09
MR-Egger Intercept	0.001 (−0.001, 0.003)	0.38
Weighted Median	0.006 (0.001, 0.010)	**0.004**
Weighted Mode	0.006 (0.001, 0.011)	**0.01**
**AD genetic risk – VT**		
IVW	0.001 (−0.001, 0.004)	0.24
MR-Egger	0.002 (−0.001, 0.005)	0.31
MR-Egger Intercept	−0.0001 (−0.0015, 0.0012)	0.86
Weighted Median	0.002 (−0.0006, 0.005)	0.12
Weighted Mode	0.002 (−0.0005, 0.005)	0.13
**AD genetic risk – VW**		
IVW	−0.004 (−0.136, 0.12)	0.95
MR-Egger	0.049 (−0.11, 0.21)	0.55
MR-Egger Intercept	−0.034 (−0.09, 0.02)	0.28
Weighted Median	0.016 (−0.09, 0.13)	0.77
Weighted Mode	0.026 (−0.09, 0.14)	0.66
**AD genetic risk – Fractal Dimension**		
IVW	0.0001 (−0.00009, 0.0004)	0.20
MR-Egger	0.0002 (−0.00008, 0.0005)	0.16
MR-Egger Intercept	−0.00005 (−0.00017, 0.00007)	0.46
Weighted Median	0.0002 (−0.00006, 0.0004)	0.14
Weighted Mode	0.0002 (−0.00004, 0.0005)	0.12

*Abbreviation*: AD=Alzheimer’s Disease; CI=confidence interval; IVW=inverse-variance weighted; AT= arteriolar tortuosity; VT= venular tortuosity; VW= venular width; FD = fractal dimension. Estimates are given for SD change per doubling odds of Alzheimer’s disease genetic risk.

In the reverse direction, there was little evidence that greater retinal arteriolar tortuosity affected AD risk (IVW regression OR=0.99, 95%CI=0.86,1.14, p-value=0.94) (Fig. 1B, Supplementary Table S18), and the heterogeneity of the causal effects estimates among the genetic instruments for AT, indicated by the Q-statistic, was moderately high (Q = 98.14;p-value=0.09)(Supplementary Table S19).

When Steiger filtering was applied, there was no reduction in genetic instruments in the analysis in either direction (Supplementary Figures S4-S5). This provided further evidence that genetic liability to AD causes greater retinal arteriolar tortuosity and the effects of the variants were acting in the correct direction. Moreover, the effects of AD on the retinal vasculature were specific to arteriolar tortuosity, since there was little evidence that AD could influence venular tortuosity (VT), venular width (VW) or retinal vessel fractal dimension (FD) (Table 1, Fig. 1A) and that these retinal vascular phenotypes (VT, VW, FD) affected AD risk (Supplementary Table S18, Fig. 1B).

### 3.2 AD may cause neurodegeneration of the inner retina but the evidence is weak

Previous studies have shown that inner retinal degeneration is associated with cognitive decline and may predict future dementia diagnosis [3]. In this study, we determined whether AD is aetiologically related to any neurodegenerative signs in the retina.

The results of IVW regression provided little statistical evidence that genetic liability to AD causes thinning of the mRNFL (IVW regression β = −0.047, 95%CI=−0.119,0.023; p-value=0.18) and mGCIPL (IVW regression β = −0.061; 95%CI=−0.14,0.025; p-value=0.16) in macular OCT scans (Table 2, Fig. 1A, Supplementary Figure S6B&C). The other MR methods used in sensitivity analyses provided consistent estimates (Table 2) but there were high levels of heterogeneity in variant effects, indicated by Cochran’s Q-statistic (Supplementary Table S20), suggesting some genetic variants may have horizontally pleiotropic effects.

**Table 2:** The causal effects of Alzheimer's disease on retinal layer thickness based on univariable Mendelian randomization.

Analysis	Estimate (95% CI)	P-value
**AD genetic risk – mRNFL thickness**		
IVW	−0.047 (−0.119, 0.023)	0.18
MR-Egger	−0.08 (−0.166, 0.006)	0.08
MR-Egger Intercept	0.02 (−0.011, 0.05)	0.22
Weighted Median	−0.065 (−0.119, −0.010)	**0.02**
Weighted Mode	−0.066 (−0.119, −0.012)	**0.02**
**AD genetic risk – mGCIPL thickness**		
IVW	−0.061 (−0.14, 0.025)	0.16
MR-Egger	−0.11 (−0.21, −0.017)	**0.03**
MR-Egger Intercept	0.035 (−0.001, 0.07)	0.07
Weighted Median	−0.093 (−0.16, −0.02)	**0.01**
Weighted Mode	−0.096 (−0.17, −0.021)	**0.02**
**AD genetic risk – Macular thickness**		
IVW	−0.009 (−0.11, 0.13)	0.88
MR-Egger	−0.039 (−0.19, 0.11)	0.62
MR-Egger Intercept	0.031 (−0.02, 0.09)	0.30
Weighted Median	−0.015 (−0.12, 0.09)	0.77
Weighted Mode	−0.012 (−0.13, 0.10)	0.84
**AD genetic risk – ONL thickness**		
IVW	−0.06 (−0.14, 0.02)	0.14
MR-Egger	−0.06 (−0.16, 0.04)	0.25
MR-Egger Intercept	0.0006 (−0.03, 0.03)	0.97
Weighted Median	−0.05 (−0.13, 0.02)	0.15
Weighted Mode	−0.06 (−0.14, 0.01)	0.12
**AD genetic risk – IS thickness**		
IVW	0.001 (−0.017, 0.02)	0.86
MR-Egger	−0.004 (−0.027, 0.018)	0.70
MR-Egger Intercept	0.003 (−0.004, 0.013)	0.38
Weighted Median	−0.0004 (−0.018, 0.017)	0.96
Weighted Mode	−0.0009 (−0.019, 0.017)	0.92
**AD genetic risk – OS thickness**		
IVW	0.044 (−0.0001, 0.08)	0.05
MR-Egger	0.059 (0.005, 0.113)	**0.04**
MR-Egger Intercept	−0.01 (−0.03, 0.009)	0.33
Weighted Median	0.053 (0.005, 0.10)	**0.02**
Weighted Mode	0.052 (0.005, 0.10)	**0.04**

*Abbreviation*: AD = Alzheimer’s Disease; CI = confidence interval; IVW = inverse-variance weighted; mRNFL = macular retinal nerve fibre layer; mGCIPL = macular ganglion cell inner plexiform layer, ONL = outer nuclear layer, IS = inner segment, OS = outer segment. Estimates are given for SD change per doubling odds of Alzheimer’s disease genetic risk.

Steiger filtering showed that 10/20 (50%) instrumental variables for AD explained more variance in macular measurements of RNFL (mRNFL); likewise, Steiger filtering showed 11/20 (55%) instrumental variables for AD explained more variance in macular measurements of GCIPL (Supplementary Figure S4). As may be expected, removing these variants from the analysis showed genetic liability to AD had stronger causal effects on thinning of the mRNFL (IVW regression β = −0.062,95%CI=−0.115,−0.008;p-value=0.02) and mGCIPL (IVW regression β = −0.076;95%CI=−0.14,−0.006;p-value=0.03), and the estimates were more consistent across MR methods with insignificant levels of heterogeneity (Supplementary Tables S21-S22). However, the results of Steiger filtering need to be interpreted with caution in this context.

There was little evidence that genetic liability to AD influenced total macular thickness (MT), the ONL or IS layer across the MR methods (Table 2, Fig. 1A), and statistically weak evidence that AD caused thickening of the OS layer (IVW regression β = 0.044; 95%CI=−0.0001,0.08;p-value=0.05)(Table 2, Fig. 1A, Supplementary Figure S6D) that was consistent in sensitivity analyses and after Steiger filtering (removal of 16/20 variants) (Supplementary Figure S4). These analyses showed little heterogeneity or evidence of horizontal pleiotropy (Table 2, Supplementary Tables S20-S22). MR analyses in the reverse direction showed that the retinal layer phenotypes (mRNFL, mGCIPL, MT, ONL, IS, and OS) did not influence AD risk (p-values≥0.22) (Fig. 1B, Supplementary Table S23).

### 3.3 Optic disc area (ODA) influences AD risk but the relationship is probably mediated by refractive error

Neurodegenerative changes affecting the retina and optic nerve can result in pathological optic disc cupping from the thinning of the neuroretinal rim. Since the neuroretinal rim is physiologically wider inferiorly and superiorly compared with nasal and temporal measurements, increased vertical cup-to-disc ratio (VCDR) can indicate pathological disc cupping. Hence, MR analyses were applied to assess the direction and strength of the causal relationships between AD and optic cup area (OCA), VCDR, and optic disc area (ODA).

In these bidirectional analyses, genetic liability to AD did not appear to influence VCDR or OCA, and nor did VCDR or OCA affect AD risk (Tables 3, Supplementary Table S24, Fig. 1A and B). However, there did appear to be good evidence that ODA influenced AD risk (IVW regression OR=0.76;95%CI=0.62,0.93;p-value=0.009)(Table 3, Fig. 1B, Supplementary Figure S6E). In a separate analysis, the causal effects of genetic liability to AD on ODA were too weak and small to be clinically important (IVW regression β = −0.009; 95%CI=−0.019,0.0005;p-value=0.06)(Supplementary Table S24, Fig. 1A, Supplementary Figure S6F). The MR-Steiger directionality test confirmed that all the genetic instruments were valid in the analysis to estimate the effect of AD liability on ODA, and in the reverse direction, the effect of ODA on AD risk (Supplementary Figures S4-S5).

**Table 3:** The causal effects of optic disc morphology on Alzheimer's disease risk based on univariable Mendelian randomization.

**Analysis**	**Causal Estimate (95% CI)**	***P-*value**
**Genetically predicted optic disc area – AD**		
IVW	**0.76 (0.62, 0.93)**	**0.009**
MR-Egger	0.73 (0.49, 1.10)	0.16
MR-Egger Intercept*	0.002 (−0.017, 0.021)	0.81
Weighted Median	0.87 (0.64, 1.18)	0.39
Weighted Mode	0.89 (0.63, 1.24)	0.51
**Genetically predicted optic cup area - AD**		
IVW	1.042 (0.92, 1.17)	0.49
MR-Egger	1.033 (0.90, 1.18)	0.64
MR-Egger Intercept*	0.0016 (−0.0096, 0.012)	0.77
Weighted Median	1.03 (0.88, 1.22)	0.64
Weighted Mode	1.03 (0.87, 1.23)	0.68
**Genetically predicted VCDR - AD**		
IVW	0.61 (0.30, 1.24)	0.17
MR-Egger	0.81 (0.22, 2.99)	0.76
MR-Egger Intercept*	−0.004 (−0.02, 0.01)	0.60
Weighted Median	0.71 (0.30, 1.66)	0.43
Weighted Mode	0.78 (0.30, 1.98)	0.61

Abbreviation: AD=Alzheimer’s Disease; CI=confidence interval; IVW=inverse-variance weighted; VCDR=vertical cup disc ratio. Causal estimates are odds ratios for Alzheimer’s Disease risk except MR-Egger Intercept*.

The size of the effect of ODA on AD was large enough to be clinically meaningful, since each SD increase in ODA decreased the odds of AD by 24%. Further sensitivity analyses using alternative MR methods provided little evidence of heterogeneity (Cochran’s Q statistic Q = 8.21; P-value=0.60) or horizontal pleiotropy (MR-Egger intercept: 0.002; 95%CI=−0.017,0.021; P-value=0.81) and were consistent in size and direction (Supplementary Figure S6E, Table 3, Supplementary Table S19). Because tilting of the optic disc is associated with longer AL and myopia (negative RE) [22], and ODA measurements can be influenced by the effects of RE on image magnification, we applied two MVMR models to estimate the direct and independent effects of ODA on AD risk while adjusting separately for RE [23] and AL [24]. In the first MVMR model, the causal effects of ODA on AD risk appeared independent of AL (Supplementary Table S25). In the second MVMR model, the causal effects of ODA on the odds of AD were attenuated after adjusting for the effects of RE (IVW estimate=0.67; 95%CI=0.36,1.25;p-value=0.21) but vulnerable to bias due to weak instrument strength (F-statistic=4.92) and the presence of pleiotropy (Q-statistic=4212;p-value=0.00)(Supplementary Table S25). This finding suggests that the effects of ODA on AD risk may be mediated by RE.

### 3.4 Additional sensitivity analyses

The main MR analyses in this study were based on a GWAS meta-analysis of AD from Kunkle et al. [7]. In further sensitivity analyses, summary data from three independent GWAS of AD (Lambert et al. [8], Jansen et al. [9] & Bellenguez et al. [10]) were used in similar MR analyses for comparison.

Using summary data from Lambert et al. [8], the results of the MR analyses were similar to Kunkle et al. [7]; both these studies had included clinically confirmed cases of AD. The other two GWAS had included AD proxy cases, and the estimates based on their summary data were much less precise (on average, the standard errors were 70% greater) and more inconsistent in direction (Supplementary Figure S7, Supplementary Tables S26-S27). Subsequent leave-one-out analyses showed the rs429358T>C variant of the *APOE* gene (representing the *APOE* ε4 allele) was a major contributor to the causal estimates based on Kunkle et al. [7] and Lambert et al. [8]; another rs4128952G>C variant (that is also associated with the *APOE ε4* allele) contributed to the causal estimates based on Jansen et al. [9] whereas the rs429358 variant was absent from the summary data and was not an instrument in the analysis; while the GWAS published by Bellenguez et al. [10] had excluded all *APOE* variants from the summary data and so there were no *APOE* variants used as instruments in the analysis (Supplementary Figures S8-S12). Therefore, the effects of the *APOE* gene were diminished in the MR analysis based on the results of the Jansen et al. study [9] due to differences in case ascertainment and the specific *APOE* variant included in the summary data, and they were excluded from the MR analysis based on the Bellenguez et al. study, which did not include *APOE* variants in their summary data [10].

### 3.5 Shared genetic effects among the ocular traits

**Fig. 2:**
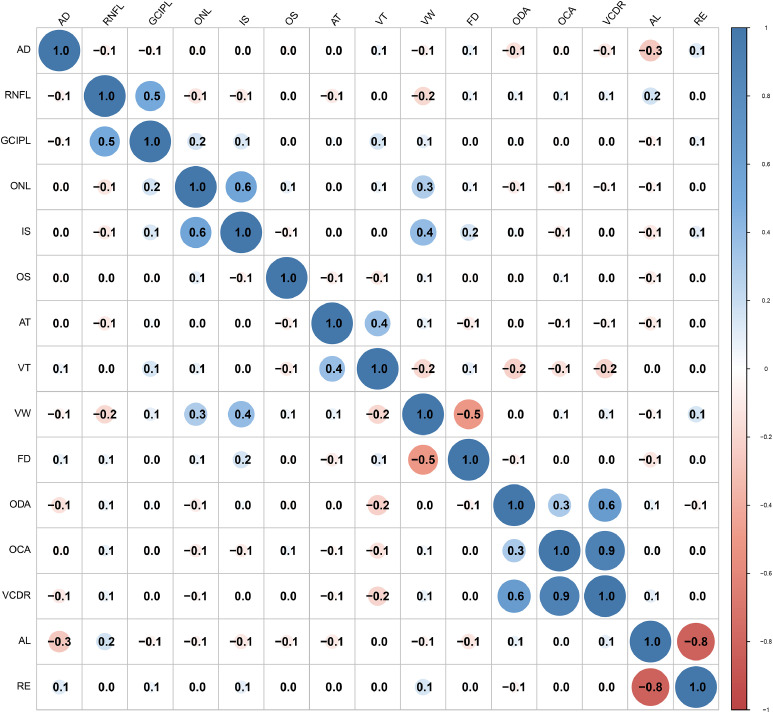
Heatmap representing the genetic correlations between the ocular traits and Alzheimer’s disease. This correlation matrix plot was visualised in R using the **corrplot** package, where the area of the circles varies with the degree of genetic correlation between the ocular traits (the absolute values are presented numerically within the circles), and the colour varies between correlations that are positive (blue) and negative (red). **Abbreviations:** AD=Alzheimer’s disease, mRNFL=macular Retinal Nerve Fibre Layer, mGCIPL=macular Ganglion Cell Inner Plexiform Layer, ONL=outer nuclear layer, and IS=inner segment layer thickness, OS=outer segment layer, AT=Arteriolar Tortuosity, VT=Venular Tortuosity, VW=Venular Width, FD=retinal vessel fractal dimension, ODA=optic disc area, OCA=optic cup area, VCDR=vertical cup disc ratio.

As some cell-types span >1 retinal layer (e.g., retinal ganglion cells contribute to the GCIPL, RNFL and optic disc), the genetic correlations between the different ocular traits and AD were explored using LDSC. As expected, there were moderate genome-wide correlations between mRNFL and mGCIPL (r_g_ = 0.51; p = 6.0e-28), and the ONL and IS layer (r_g_ = 0.55; p = 6.5e-18). Although the optic disc phenotypes showed modest-to-high correlations with each other (r_g_ = 0.3–0.9; p < 0.0001), there were minimal-to-no correlations with mRNFL and mGCIPL thickness. Among the vascular phenotypes, AT and VT were positively correlated (r_g_ = 0.37; p = 1.9e-09), and there was a negative correlation between VW and fractal dimension (r_g_ = −0.51; p = 4.1e-07, Fig. 2, Supplementary Table S28). Hence, many of the ocular traits showed moderate-high genetic correlations and were not independent phenotypes in the MR analysis.

## 4 Discussion

Observational studies have previously reported AD is associated with several ocular traits that have been proposed as predictors or biomarkers of the disease. However, previous studies were ill-suited to validate these ocular traits as early manifestations of AD or to investigate their aetiology. In contrast, MR has been likened to a randomized trial by genotype and is a powerful method to investigate the causal effects of AD on a range of neurovascular and neuroretinal traits. Since AD diagnosis is often preceded by a long prodrome of several years, there is huge potential value in diagnosing AD earlier, based on an eye examination, when interventions may have greater impact on the outcome.

Previous studies have mainly focused their attention on the association between AD and inner retinal degeneration. In a systematic review and meta-analysis of 25 studies, total MT and peripapillary RNFL (pRNFL) thickness were significantly reduced in 887 patients with established AD compared with 864 healthy controls [25]. In a cohort study (n = 3170) of people with different degrees of cognitive impairment (including healthy, mild, and all-cause dementia), a polygenic risk score (PRS) for mGCIPL (but not mRNFL) thickness was associated with all-cause dementia; however, the PRS for AD did not predict mRNFL or mGCIPL thickness [26]. Similarly, the results of the much larger analysis of UK Biobank (UKB) participants (n = 31,434) in this study have provided little statistical support for the proposition that late-onset AD causes inner retinal degeneration, as evidenced by thinning of the GCIPL and RNFL in macular OCT scans. There are several possible explanations and sources of bias to explain these MR results: first, UKB participants were relatively young (aged 40–69 years) at the time of recruitment and few had been diagnosed with AD, so the phenotypic consequences of the genetic variants acting on these features through AD may not have fully manifested, even among individuals with genetically higher risk of AD; second, OCT measures were derived from a subset of UKB participants who had images above a specific quality threshold for analysis, which may have excluded the participants who were most likely to be affected by the traits of interest; third, macular RNFL and GCIPL measurements may be less sensitive to neurodegeneration than peripapillary measurements; fourth, inner retinal thinning is not specific to dementia and may be caused by intrinsic ocular diseases and secondary degeneration of the optic nerve from other brain insults. Although it is very plausible that neurodegeneration of the inner retina may be an early manifestation of AD, the very large sample sizes and statistical power of the MR analysis in this study failed to provide much support for this hypothesis.

Age-related macular degeneration (AMD) is another common age-related neurodegenerative disease affecting the eye. AMD is characterised by drusen deposition and atrophy of the outer retina/RPE, causing an irregular thickening and thinning of the OS/RPE layers. Amyloid-beta protein is detected in drusen and there are studies reporting drusen are significantly more prevalent in the outer retina of AD patients than healthy controls [27], suggesting that AD and AMD may share similar pathogenic pathways. However, the evidence that AD can cause any of the characteristic ocular features of AMD is conflicting [[28], [29], [30]]. Similarly, the results of this study provided weak statistical evidence that genetic liability to late-onset AD had any influence on the thickness of the OS layer. Since AMD can cause irregular thickening and thinning of the OS/RPE, the MR analysis in this study was not ideally suited to test this hypothesis. Hence, other methods will be required to understand whether AMD and AD share common pathogenic mechanisms.

The amyloid-beta plaques that characterize AD pathology are frequently located close to cerebral microvasculature and within cerebral blood vessel walls. Besides AD, the *APOe4* haplotype is a risk factor for atherosclerosis and stroke [31], and *APOe4-*knock in mice have been shown to demonstrate increased retinal vascular tortuosity, inner retinal thinning and reduced visual function [32]. Hence, vascular disease is a significant component of AD aetiology [31]. Since direct investigation of the cerebral microvasculature is not possible in AD patients, exploring the relationship between AD and the retinal vasculature provides an alternative approach to determine the effects of vascular risk factors on AD risk, as well as the impact of AD on cerebrovascular health. In this study, summary data from the largest currently available GWAS of retinal AT, VT, VW, and FD (n ≥ 38,000) were used in bidirectional MR analyses and showed that among these vascular traits, genetic liability to AD had a specific effect on increasing retinal arteriolar tortuosity. This finding in UKB participants - who do not yet have the disease - suggests that genetic liability to AD causes retinal and cerebrovascular changes from amyloid angiopathy that precede the clinical manifestations of the disease, and that interventions which target these early vascular changes may have therapeutic value. Additionally, it may be possible to assess the efficacy of new treatments for AD by monitoring their effects on the retinal microvasculature.

The results of this study also showed that AD risk was, on average, 24% lower per SD increase in ODA; but in a MVMR model, this relationship was probably mediated by refractive error (RE). Myopia (short-sight) is associated with longer ALs and tilting of the optic disc, which will affect its cross-sectional area [22]. Moreover, RE influences the magnification of ophthalmic imaging and measurements based on these images. The relevance is that higher levels of educational attainment increase the risk of myopia while being protective against AD, suggesting horizontal pleiotropy in the MR analysis. Therefore, the relationship between ODA and AD is likely mediated by the causal and pleiotropic effects of higher levels of educational attainment on RE and AD.

There are further limitations to the analyses in this study and possible sources of bias. First, the GWAS summary data for the ocular traits (except optic disc morphology) were adjusted for heritable covariates (i.e., weight, height, refraction) and image quality, which can yield biased estimates in subsequent two-sample MR studies. Second, many of the ocular traits were genetically correlated, and so there was no adjustment in the analysis for multiple comparisons. Third, the results of Steiger filtering should be interpreted with caution since it is not well suited to measuring the direction of causality in analyses involving binary exposures or traits that are vulnerable to measurement errors. Fourth, most image-derived phenotypes in the analyses were obtained from UKB participants who are largely healthier than the general population, and younger than the typical AD patient. Fifth, the image-derived phenotypes in this study are vulnerable to systematic errors arising from the acquisition, quality, and automated analysis of the images in the original GWAS. Finally, the GWAS cohorts in this study were of European ancestry, meaning the results may not be representative of other ethnicities due to the differences in ocular anatomy and genetics of AD between ancestral groups.

## 5 Conclusion

In conclusion, this study provides compelling evidence that genetic liability to AD causes increased retinal arteriolar tortuosity, likely from amyloid microangiopathy; however, the causal relationship between AD and neurodegeneration of the inner retina was much weaker. These findings suggest that early cerebrovascular manifestations of AD may be detected in the eye and could prove to be a useful biomarker to monitor disease progression or treatment response in clinical research studies.

## Use of generative AI and AI-assisted technologies

This research has not used any generative AI and AI-assisted technologies in scientific writing and in figures, images, and artwork.

## Author contributions

DA, ELA, and NMD devised the study concept and design. HK and AB-A performed the statistical analysis. HK, AB-A, ALH, ELA, and DA wrote the manuscript. JW, AV, MOB, XJ, CO, JNCB, JLH, LRP, SM, and XRG provided summary GWAS data from the NEIGHBORHOOD consortium. CJ and HC provided GWAS summary statistics from the GERA cohort. All authors were involved in reviewing and editing the manuscript.

## Funding

ABA, GDS, and ALH work within the MRC Integrative Epidemiology Unit at the University of Bristol, which is supported by the Medical Research Council (MC_UU_00032/1&9). Additionally, ABA was funded by grant from Fight for Sight (SGA18_011). NMD was supported by a Norwegian Research Council Grant number 295989. The NEIGHBORHOOD consortium was supported, in part, by NIH R01 EY015473 (LRP), NIH R01 EY022305 (JLH, JNCB, JLW), NIH R01 EY033829 (JNCB). HC was supported by NIH NEI R01EY027004.

## Consent statement

The study used GWAS summary data, and ethical approval was granted for each GWAS when it was performed. No additional consent was necessary for this study.

## Data sharing statement

The genetic instruments used to perform the MR analyses in this study are provided in the supplementary material. All GWAS summary data used in this study are publicly available or can be provided by the authors upon request.

## Declaration of competing interest

DA is on the clinical advisory board of Siloton Ltd. GDS reports grants from the MRC Integrative Epidemiology Unit at the University of Bristol; he is a member of the Scientific Advisory Board for Bristol Myers Squibb, Relation Therapeutics and Insitro. JLW reports consulting fees from Editas and CRISPR Therapeutics. MOB reports research grants from Gates Ventures, LifeArc, Eisai, Wellcome Leap, Optos and UKRI; and consulting fees from LifeArc. SM is a director of Seonix Pty Ltd, a company commercialising polygenic risk scores, and he holds equity in Seonix Pty Ltd. HK, ABA, JNCB, AVV, XJ, CGO, JLH, LRP, XRG, CJ, HC, PGK, NMD, ALH, and ELA have no conflicts of interest.

## Appendix Supplementary materials

Image, application 1

Image, application 2
