# Supplementary material for: Can we identify people with Alzheimer’s disease from examination of the eye? A bidirectional Mendelian randomization (MR) study
Source: J Prev Alzheimers Dis. 2026 Jul 10;13(8):100635. doi: 10.1016/j.tjpad.2026.100635 (PMC13380462; doi:10.1016/j.tjpad.2026.100635)
Supplement: Supplementary file 1 [file mmc1.docx]

**STROBE-MR checklist of recommended items to address in reports of Mendelian randomization studies**^1^ ^2^

| **Item No.** | **Section** | **Checklist item** | **Page No.** | **Relevant text from manuscript** |
| --- | --- | --- | --- | --- |
| 1 | **TITLE and ABSTRACT** | Indicate Mendelian randomization (MR) as the study’s design in the title and/or the abstract if that is a main purpose of the study | Pages 1 & 3 | The title of the manuscript is: Can we identify people with Alzheimer’s disease from examination of the eye? A bidirectional Mendelian randomization (MR) study.  In the abstract: Bidirectional two-sample Mendelian randomization (MR) analyses using instrument variables derived from genome-wide association studies of AD and imaging of the retina, optic disc, and retinal vasculature. |
|  | **INTRODUCTION** |  |  |  |
| 2 | **Background** | Explain the scientific background and rationale for the reported study. What is the exposure? Is a potential causal relationship between exposure and outcome plausible? Justify why MR is a helpful method to address the study question | Pages 4, 5 | Past studies have shown that inner retinal neurodegeneration is associated with brain MRI markers of neurodegeneration, cognitive decline and dementia…however, a recent systematic review has highlighted the heterogeneity of these studies and limitations in their study design that could lead to bias….MR is less vulnerable to bias from confounding and reverse causation than observational studies, and can provide greater insight into the neurovascular and neuroretinal changes in the eye that are directly caused by AD rather than associations with the disease. |
| 3 | **Objectives** | State specific objectives clearly, including pre-specified causal hypotheses (if any). State that MR is a method that, under specific assumptions, intends to estimate causal effects | Page 3 abstract, page 5-6, supplementary pages 4, 12-13 | Neurodegeneration in Alzheimer’s disease (AD) is thought to be driven by amyloid-beta and tau deposition in the cerebral vasculature and brain. As the eye is an extension of the central nervous system, this study aimed to determine which neurovascular and neuroretinal changes in the eye are caused by AD rather than associations of the disease.  MR is a statistical method that uses genetic variants (including single-nucleotide polymorphisms or SNPs) that are strongly associated with an exposure in GWAS as instrumental variables to estimate the causal effects of the exposure on the trait of interest. Additional details about the core assumptions of MR and the differences between MR methods are provided in Supplementary Methods. |
|  | **METHODS** |  |  |  |
| 4 | **Study design and data sources** | Present key elements of the study design early in the article. Consider including a table listing sources of data for all phases of the study. For each data source contributing to the analysis, describe the following: |  |  |
|  | a) | Setting: Describe the study design and the underlying population, if possible. Describe the setting, locations, and relevant dates, including periods of recruitment, exposure, follow-up, and data collection, when available. | Page 5-6  Supplementary  pages 4-11, 24-25 | In this two-sample MR study, genetic variant (instrument) associations with the exposures and instrument associations with the outcomes were obtained from GWAS of non-overlapping samples to estimate the causal effects of genetic liability to AD on each ocular trait. In separate MR analyses, the causal effects of genetic liability to each ocular trait on AD risk were estimated in the other direction… (Supplementary Methods, Supplementary Table S1). |
|  | b) | Participants: Give the eligibility criteria, and the sources and methods of selection of participants. Report the sample size, and whether any power or sample size calculations were carried out prior to the main analysis | Page 6  Supplementary  Pages 4-11, 24-25 | GWAS summary data for late-onset AD were obtained through the IEU Open GWAS project; likewise, summary data for the ocular traits were obtained from published GWAS. Further details about participant ascertainment, recruitment, genotyping, and imaging/phenotyping procedures are available in Supplementary Methods and Supplementary Table S1. |
|  | c) | Describe measurement, quality control and selection of genetic variants | Page 6  Supplementary  Pages 4-16, 24-25 | Genetic instruments for each exposure and outcome were selected following the same pipeline (Supplementary Figures S3-S5). Only instruments with an F-statistic>10 were retained in univariable MR analyses to minimise weak instrument bias (Supplementary Tables S2-S17). |
|  | d) | For each exposure, outcome, and other relevant variables, describe methods of assessment and diagnostic criteria for diseases | Page 7  Supplementary  Pages 3-10 | Further details about participant ascertainment, recruitment, genotyping, and imaging/phenotyping procedures are available in Supplementary Methods and Supplementary Table S1. |
|  | e) | Provide details of ethics committee approval and participant informed consent, if relevant | Page 6  Supplementary  Page 4-11 | Ethical approval was granted for each GWAS. Further details about participant ascertainment, recruitment, genotyping, and imaging/phenotyping procedures are available in Supplementary Methods and Supplementary Table S1. |
| 5 | **Assumptions** | Explicitly state the three core IV assumptions for the main analysis (relevance, independence and exclusion restriction) as well assumptions for any additional or sensitivity analysis | Supplementary  Pages 4, 12 | MR analyses are based on several core assumptions: (1) the genetic variants are robustly associated with the exposure; (2) there are no common confounders of the genetic variants and the outcome; (3) the genetic variants do not influence the outcome via biological pathways independent of the exposure under investigation (horizontal pleiotropy)(Figure S2). |
| 6 | **Statistical methods: main analysis** | Describe statistical methods and statistics used |  |  |
|  | a) | Describe how quantitative variables were handled in the analyses (i.e., scale, units, model) | Page 6 | The results were transformed into SD units per doubling of odds of genetic liability to AD risk to compare the magnitude of the effects across different ocular traits. For analyses in the reverse direction, causal estimates were expressed in odds ratio (OR) per SD increase in each ocular trait |
|  | b) | Describe how genetic variants were handled in the analyses and, if applicable, how their weights were selected | Page 7  Supplementary Page 4 | The effect of each exposure on the outcome was estimated using inverse-variance weighted (IVW) regression. Alternative MR methods (MR-Egger, weighted median, weighted mode) were used in sensitivity analyses to test for horizontal pleiotropy. Inverse-variance weighted (IVW) regression is a MR method, in which the SNP-exposure and SNP-outcome associations are combined in a random effects meta-analysis.  However, MR estimates can be biased by SNPs that act via horizontally pleiotropic pathways, which would invalidate one of the key assumptions of MR analyses described above. An alternative method, MR-Egger regression gives a valid causal estimate under the InSIDE assumption, where each SNP-exposure association is independent of the direct pleiotropic effect of the SNP on the outcome; in MR-Egger regression, deviation of the intercept estimate from zero suggests the existence of directional horizontal pleiotropy. Additionally, the weighted median provides consistent causal estimates when >50% of the information in the analysis comes from valid genetic instruments, while the weighted mode will provide a robust causal estimate in the presence of pleiotropy when SNPs producing the most common MR estimate have no horizontal pleiotropic effects. The study was conducted and reported following the STROBE-MR guidelines |
|  | c) | Describe the MR estimator (e.g. two-stage least squares, Wald ratio) and related statistics. Detail the included covariates and, in case of two-sample MR, whether the same covariate set was used for adjustment in the two samples | Page 6  Supplementary  Pages 4-8 | Two-sample MR analyses were applied using the Wald ratios method in the TwoSampleMR package  Details of the adjustments for covariates in the original GWAS summary data are described in the supplementary file. |
|  | d) | Explain how missing data were addressed | NA | NA |
|  | e) | If applicable, indicate how multiple testing was addressed | Pages 7-8 & 15 | Linkage disequilibrium score regression (LDSC) was used to estimate the genetic correlations between the ocular traits and AD based on the GWAS summary data.  Many of the ocular traits were genetically correlated, and so there was no adjustment in the analysis for multiple comparisons. |
| 7 | **Assessment of assumptions** | Describe any methods or prior knowledge used to assess the assumptions or justify their validity | Supplementary Page 4 | MR-Egger regression gives a valid causal estimate under the InSIDE assumption, where each SNP-exposure association is independent of the direct pleiotropic effect of the SNP on the outcome. Deviation of the intercept estimate from zero in MR-Egger regression suggests the existence of directional horizontal pleiotropy. |
| 8 | **Sensitivity analyses and additional analyses** | Describe any sensitivity analyses or additional analyses performed (e.g. comparison of effect estimates from different approaches, independent replication, bias analytic techniques, validation of instruments, simulations) | Pages 7 | Sensitivity analyses were performed using MR Egger, weighted median and weighted mode to look for evidence of horizontal pleiotropy The Q-statistic was used to assess heterogeneity in the estimated effects of each exposure, which can also indicate the presence of pleiotropy.  The main MR analyses in this study were based on a GWAS meta-analysis of AD from Kunkle *et al*. In further sensitivity analyses, summary data from three independent GWAS of AD (Lambert *et al*, Jansen *et al* & Bellenguez *et al*) were used in similar MR analyses for comparison. |
| 9 | **Software and pre-registration** |  |  |  |
|  | a) | Name statistical software and package(s), including version and settings used | Page 6  Supplementary Page 11 | R version 3.6.1 (two-sample MR package and MVMR package) |
|  | b) | State whether the study protocol and details were pre-registered (as well as when and where) | NA | NA |
|  | **RESULTS** |  |  |  |
| 10 | **Descriptive data** |  |  |  |
|  | a) | Report the numbers of individuals at each stage of included studies and reasons for exclusion. Consider use of a flow diagram | Page 6  Supplementary  Pages 4-11 | Further details about participant ascertainment, recruitment, genotyping, and imaging/phenotyping procedures are available in Supplementary Methods and Supplementary Table S1. |
|  | b) | Report summary statistics for phenotypic exposure(s), outcome(s), and other relevant variables (e.g. means, SDs, proportions) |  | These details were provided in their respective GWAS papers |
|  | c) | If the data sources include meta-analyses of previous studies, provide the assessments of heterogeneity across these studies | NA | NA |
|  | d) | For two-sample MR:  i.  Provide justification of the similarity of the genetic variant-exposure associations between the exposure and outcome samples  ii.  Provide information on the number of individuals who overlap between the exposure and outcome studies | Page 5 | In this two-sample MR study, genetic variant (instrument) associations with the exposures and instrument associations with the outcomes were obtained from GWAS of non-overlapping samples to estimate the causal effects of genetic liability to AD on each ocular trait. |
| 11 | **Main results** |  |  |  |
|  | a) | Report the associations between genetic variant and exposure, and between genetic variant and outcome, preferably on an interpretable scale | Supplementary pages 26-48 | Supplementary Table S2-S17 |
|  | b) | Report MR estimates of the relationship between exposure and outcome, and the measures of uncertainty from the MR analysis, on an interpretable scale, such as odds ratio or relative risk per SD difference | Pages 8-12  Supplementary pages 49, 52, 54-62 | The effect of each exposure on the outcome was estimated using inverse-variance weighted (IVW) regression; the results were transformed into SD units per doubling of odds of genetic liability to AD risk to compare the magnitude of the effects across different ocular traits. For analyses in the reverse direction, causal estimates were expressed in odds ratio (OR) per SD increase in each ocular trait. These results are reported in Tables 1-3, supplementary Tables S18, S21, S23-S27 |
|  | c) | If relevant, consider translating estimates of relative risk into absolute risk for a meaningful time period | NA | NA |
|  | d) | Consider plots to visualize results (e.g. forest plot, scatterplot of associations between genetic variants and outcome versus between genetic variants and exposure) | Pages 8-12  Supplementary pages 17-23 | Figure 1, supplementary Figure: S6-S12 |
| 12 | **Assessment of assumptions** |  |  |  |
|  | a) | Report the assessment of the validity of the assumptions | Page 8-11 | The results of sensitivity analyses using alternate MR methods and the likely presence of pleiotropy (based on the MR-Egger intercept and Q-statistic) and weak instrument bias (based on F-statistic) are reported for each MR estimate. |
|  | b) | Report any additional statistics (e.g., assessments of heterogeneity across genetic variants, such as *I^2^*, Q statistic or E-value) | Page 8-11 | The Q-statistic was used to assess heterogeneity in the estimated effects of each exposure, which can also indicate the presence of pleiotropy |
| 13 | **Sensitivity analyses and additional analyses** |  |  |  |
|  | a) | Report any sensitivity analyses to assess the robustness of the main results to violations of the assumptions | Pages 7 | Sensitivity analyses were performed using MR Egger, weighted median and weighted mode to look for evidence of horizontal pleiotropy The Q-statistic was used to assess heterogeneity in the estimated effects of each exposure, which can also indicate the presence of pleiotropy.  Only instruments with an F-statistic>10 were retained to minimise weak instrument bias in univariable MR analyses. Additionally, leave-one-out analyses were conducted. Moreover, MR analyses were repeated using summary data from 3 other independent GWAs of AD. |
|  | b) | Report results from other sensitivity analyses or additional analyses | Page 9-12  Supplementary pages 50-62 | The results of the sensitivity analyses listed above are reported in the main results and supplementary material. |
|  | c) | Report any assessment of direction of causal relationship (e.g., bidirectional MR) | Page 8-12 | All MR analyses were conducted in both directions. |
|  | d) | When relevant, report and compare with estimates from non-MR analyses |  | Not relevant |
|  | e) | Consider additional plots to visualize results (e.g., leave-one-out analyses) | Supplementary pages 19-23 | Supplementary Figure: S8-S12 |
|  | **DISCUSSION** |  |  |  |
| 14 | **Key results** | Summarize key results with reference to study objectives | Page 16 | This study aimed to determine which neurovascular and neuroretinal changes in the eye are caused by AD rather than associations of the disease. The results have provided compelling evidence that genetic liability to AD causes increased retinal arteriolar tortuosity; however, the causal relationship between AD and neurodegeneration of the inner retina was much weaker. |
| 15 | **Limitations** | Discuss limitations of the study, taking into account the validity of the IV assumptions, other sources of potential bias, and imprecision. Discuss both direction and magnitude of any potential bias and any efforts to address them | Pages 15-16 | There are several limitations to the analyses in this study and possible sources of bias that are discussed in detail in the main manuscript (pp15-16).. |
| 16 | **Interpretation** |  |  |  |
|  | a) | Meaning: Give a cautious overall interpretation of results in the context of their limitations and in comparison with other studies | Pages 12-16 | Previous studies have mainly focused their attention on the association between AD and inner retinal degeneration.…. the results of the much larger analysis of UK Biobank (UKB) participants (n=31,434) in this study have provided weak statistical support for the proposition that late-onset AD causes inner retinal degeneration… and…genetic liability to AD had a specific effect on increasing retinal arteriolar tortuosity. There are several possible explanations and sources of bias to explain these MR results. |
|  | b) | Mechanism: Discuss underlying biological mechanisms that could drive a potential causal relationship between the investigated exposure and the outcome, and whether the gene-environment equivalence assumption is reasonable. Use causal language carefully, clarifying that IV estimates may provide causal effects only under certain assumptions | Pages 14-16 | The amyloid-beta plaques that characterize AD pathology are frequently located close to cerebral microvasculature and within cerebral blood vessel walls. Besides AD, the *APOe4* haplotype is a risk factor for atherosclerosis and stroke, and *APOe4-*knock in mice have been shown to demonstrate increased retinal vascular tortuosity, inner retinal thinning and reduced visual function. Hence, vascular disease is a significant component of AD aetiology. |
|  | c) | Clinical relevance: Discuss whether the results have clinical or public policy relevance, and to what extent they inform effect sizes of possible interventions | Page 15-16 | …genetic liability to AD had a specific effect on increasing retinal arteriolar tortuosity. This finding in UKB participants - who do not yet have the disease - suggests that genetic liability to AD causes retinal and cerebrovascular changes from amyloid angiopathy that precede the clinical manifestations of the disease, and that interventions which target these early vascular changes may have therapeutic value. Additionally, it may be possible to assess the efficacy of new treatments for AD by monitoring their effects on the retinal microvasculature. |
| 17 | **Generalizability** | Discuss the generalizability of the study results (a) to other populations, (b) across other exposure periods/timings, and (c) across other levels of exposure | Page 16 | The GWAS cohorts in this study were of European ancestry, meaning the results may not be representative of other ethnicities due to differences in genetics of AD between ancestral groups. |
|  | **OTHER INFORMATION** |  |  |  |
| 18 | **Funding** | Describe sources of funding and the role of funders in the present study and, if applicable, sources of funding for the databases and original study or studies on which the present study is based | Page 17 | ABA, GDS, and ALH work within the MRC Integrative Epidemiology Unit at the University of Bristol, which is supported by the Medical Research Council (MC_UU_00032/1&9). Additionally, ABA was funded by grant from Fight for Sight (SGA18_011). NMD was supported by a Norwegian Research Council Grant number 295989. The NEIGHBORHOOD consortium was supported, in part, by NIH R01 EY015473 (LRP), NIH R01 EY022305 (JLH, JNCB, JLW), NIH R01 EY033829 (JNCB). HC was supported by NIH NEI R01EY027004. |
| 19 | **Data and data sharing** | Provide the data used to perform all analyses or report where and how the data can be accessed, and reference these sources in the article. Provide the statistical code needed to reproduce the results in the article, or report whether the code is publicly accessible and if so, where | Page 17-18 | The genetic instruments used to perform the MR analyses in this study are provided in the supplementary material. All GWAS summary data used in this study are publicly available or can be provided by the authors upon request. |
| 20 | **Conflicts of Interest** | All authors should declare all potential conflicts of interest | Page 17 | DA is on the clinical advisory board of Siloton Ltd. GDS reports grants from the MRC Integrative Epidemiology Unit at the University of Bristol; he is a member of the Scientific Advisory Board for Bristol Myers Squibb, Relation Therapeutics and Insitro. JLW reports consulting fees from Editas and CRISPR Therapeutics. MOB reports research grants from Gates Ventures, LifeArc, Eisai, Wellcome Leap, Optos and UKRI; and consulting fees from LifeArc. SM is a director of Seonix Pty Ltd, a company commercialising polygenic risk scores, and he holds equity in Seonix Pty Ltd. HK, ABA, JCB, AVV, XJ, CGO, JLH, LRP, XRG, CJ, HC, PGK, NMD, ALH, and ELA have no conflicts of interest. |

This checklist is copyrighted by the Equator Network under the Creative Commons Attribution 3.0 Unported (CC BY 3.0) license.

1. Skrivankova VW, Richmond RC, Woolf BAR, Yarmolinsky J, Davies NM, Swanson SA, et al. Strengthening the Reporting of Observational Studies in Epidemiology using Mendelian Randomization (STROBE-MR) Statement. JAMA. 2021;under review.

2. Skrivankova VW, Richmond RC, Woolf BAR, Davies NM, Swanson SA, VanderWeele TJ, et al. Strengthening the Reporting of Observational Studies in Epidemiology using Mendelian Randomisation (STROBE-MR): Explanation and Elaboration. BMJ. 2021;375:n2233.
