# Supplementary material for: Can we identify people with Alzheimer’s disease from examination of the eye? A bidirectional Mendelian randomization (MR) study
Source: J Prev Alzheimers Dis. 2026 Jul 10;13(8):100635. doi: 10.1016/j.tjpad.2026.100635 (PMC13380462; doi:10.1016/j.tjpad.2026.100635)
Supplement: Supplementary file 2 [file mmc2.docx]

**Can we identify people with Alzheimer’s disease from examination of the eye? A bidirectional Mendelian randomization (MR) study**

**Kiser et al**

**Supplementary Information**

[1. Supplementary Methods 4](#_Toc213766720)

[1.1 Core assumptions and rationale for different MR methods 4](#_Toc213766721)

[1.2 Genome-wide association studies (GWAS) used for Mendelian randomisation (MR) analysis 4](#_Toc213766722)

[1.2.1 GWAS Of Late Onset Alzheimer’s Disease 4](#_Toc213766723)

[1.1.2 GWAS of retinal structure and vasculature 5](#_Toc213766724)

[1.1.3 GWAS of optic disc morphology 7](#_Toc213766725)

[1.1.4 GWAS of refractive error and axial length 7](#_Toc213766726)

[1.2 Selection of genetic instruments for Mendelian randomisation analyses 8](#_Toc213766727)

[1.2.1 Alzheimer’s Disease 8](#_Toc213766728)

[1.2.2 Macular thickness 9](#_Toc213766729)

[1.2.3 Retinal nerve fibre layer thickness 9](#_Toc213766730)

[1.2.4 Ganglion cell inner plexiform layer thickness 9](#_Toc213766731)

[1.2.5 Outer nuclear layer thickness 9](#_Toc213766732)

[1.2.6 Inner segment layer thickness 9](#_Toc213766733)

[1.2.7 Outer segment layer thickness 10](#_Toc213766734)

[1.2.8 Arteriolar tortuosity 10](#_Toc213766735)

[1.2.9 Venular tortuosity 10](#_Toc213766736)

[1.2.10 Venular width 10](#_Toc213766737)

[1.2.11 Retinal vessel fractal dimension 10](#_Toc213766738)

[1.2.12 Optic disc area 10](#_Toc213766739)

[1.2.13 Optic cup area 10](#_Toc213766740)

[1.2.14 Vertical cup:disc ratio 11](#_Toc213766741)

[1.3 Selection of genetic instruments for multivariable Mendelian randomization analyses 11](#_Toc213766742)

[1.3.1 refractive error 11](#_Toc213766743)

[1.3.2 axial length 11](#_Toc213766744)

[1.3.3 Combined selection of genetic instruments for MVMR 11](#_Toc213766745)

[2. Supplementary Figures 12](#_Toc213766746)

[Figure S1. GWAS summary data used in the Mendelian randomization analyses of this study. 12](#_Toc213766747)

[Figure S2. Visual representation of the possible causal relationships between AD and the ocular traits that are tested in this study. 12](#_Toc213766748)

[Figure S3. Pipeline for defining genetic instruments 14](#_Toc213766749)

[Figure S4. Pipeline for genetic instrument selection for Alzheimer’s Disease 15](#_Toc213766750)

[Figure S5. Pipelines for genetic instrument selection for each of the ocular traits 16](#_Toc213766751)

[Figure S6. Scatter plots showing the causal relationships between Alzheimer's disease and selected ocular traits. 17](#_Toc213766752)

[Figure S7. Forest plot summarising the causal associations between AD and the ocular traits in sensitivity analyses based on summary data from 3 other independent GWAS of AD 18](#_Toc213766753)

[Figure S8: Mendelian Randomization leave-one-out sensitivity analysis of the causal effect of AD on AT based on the summary data of four independent GWAS of AD. 19](#_Toc213766754)

[Figure S9: Mendelian Randomization leave-one-out sensitivity analysis of the causal effect of AD on mRNFL thickness based on the summary data of four independent GWAS of AD. 20](#_Toc213766755)

[Figure S10: Mendelian Randomization leave-one-out sensitivity analysis of the causal effect of AD on mGCIPL thickness based on the summary data of four independent GWAS of AD. 21](#_Toc213766756)

[Figure S11: Mendelian Randomization leave-one-out sensitivity analysis of the causal effect of AD on OS layer thickness based on the summary data of four independent GWAS of AD. 22](#_Toc213766757)

[Figure S12: Mendelian Randomization leave-one-out sensitivity analysis of the causal effect of AD on ODA based on the summary data of four independent GWAS of AD. 23](#_Toc213766758)

[3. Supplementary Tables 24](#_Toc213766759)

[Table S1: Summary of GWAS data sources 24](#_Toc213766760)

[Table S2. Genetic instruments for Alzheimer’s Disease 26](#_Toc213766761)

[Table S3. Genetic instruments for macular thickness 27](#_Toc213766762)

[Table S4. Genetic instruments for macular retinal nerve fibre layer thickness 29](#_Toc213766763)

[Table S5. Genetic instruments for macular ganglion cell inner plexiform layer thickness 30](#_Toc213766764)

[Table S6. Genetic instruments for retinal outer nuclear layer thickness 31](#_Toc213766765)

[Table S7. Genetic instruments for retinal inner segment thickness 33](#_Toc213766766)

[Table S8. Genetic instruments for retinal outer segment thickness 34](#_Toc213766767)

[Table S9. Genetic instruments for retinal arteriolar tortuosity 35](#_Toc213766768)

[Table S10: Genetic instruments for retinal venular tortuosity 37](#_Toc213766769)

[Table S11. Genetic instruments for retinal venular width 38](#_Toc213766770)

[Table S12. Genetic instruments for retinal vessel fractal dimension 39](#_Toc213766771)

[Table S13. Genetic instruments for optic disc area 40](#_Toc213766772)

[Table S14. Genetic instruments for optic cup area 41](#_Toc213766773)

[Table S15. Genetic instruments for vertical cup:disc ratio 42](#_Toc213766774)

[Table S16. Genetic instruments for refractive error 43](#_Toc213766775)

[Table S17. Genetic instruments for axial length 48](#_Toc213766776)

[Table S18: Mendelian Randomization (MR) analyses for the causal effect of retinal vascular phenotypes on Alzheimer's Disease risk. 49](#_Toc213766777)

[Table S19. Levels of heterogeneity in MR analysis of exposure to ocular traits on Alzheimer’s disease risk before Steiger filtering. 50](#_Toc213766778)

[Table S20. Levels of heterogeneity in MR analysis of AD exposure on ocular traits before Steiger filtering. 50](#_Toc213766779)

[Table S21. Mendelian Randomization analyses for the causal effect of Alzheimer's disease on retinal nerve fibre layer thickness after Steiger filtering 52](#_Toc213766780)

[Table S22. Levels of heterogeneity in MR analysis of AD exposure on ocular traits after Steiger filtering 52](#_Toc213766781)

[Table S23: Mendelian Randomization (MR) analyses for the causal effect of the retinal phenotypes on Alzheimer's Disease risk 54](#_Toc213766782)

[Table S24. The causal effects of Alzheimer's disease on optic disc morphology based on univariable Mendelian randomization. 55](#_Toc213766783)

[Table S25: The direct causal effects of optic disc area, refractive error and axial length on Alzheimer’s Disease risk using multivariable Mendelian randomization. 55](#_Toc213766784)

[Table S26. The causal effects of Alzheimer's disease on ocular features based on univariable Mendelian randomization for other AD GWASs. 57](#_Toc213766785)

[Table S27. The causal effects of ocular features on Alzheimer's disease based on univariable Mendelian randomization for other AD GWASs. 60](#_Toc213766786)

[Table S28. Genetic correlations between ocular traits or Alzheimer’s disease. 63](#_Toc213766787)

[Supplementary References 66](#_Toc213766788)

# Supplementary Methods

## **Core assumptions and rationale for different MR methods**

MR analyses are based on several core assumptions: (1) the genetic variants are robustly associated with the exposure; (2) there are no common confounders of the genetic variants and the outcome; (3) the genetic variants do not influence the outcome via biological pathways independent of the exposure under investigation (horizontal pleiotropy)(Figure S2).

Inverse-variance weighted (IVW) regression is a MR method, in which the SNP-exposure and SNP-outcome associations are combined in a random effects meta-analysis. However, MR estimates can be biased by SNPs that act via horizontally pleiotropic pathways, which would invalidate one of the key assumptions of MR analyses described above. An alternative method, MR-Egger regression gives a valid causal estimate under the InSIDE assumption, where each SNP-exposure association is independent of the direct pleiotropic effect of the SNP on the outcome; in MR-Egger regression, deviation of the intercept estimate from zero suggests the existence of directional horizontal pleiotropy. [1] Additionally, the weighted median provides consistent causal estimates when >50% of the information in the analysis comes from valid genetic instruments, [1] while the weighted mode will provide a robust causal estimate in the presence of pleiotropy when SNPs producing the most common MR estimate have no horizontal pleiotropic effects. [2] The study was conducted and reported following the STROBE-MR guidelines. [3]

Typical univariable MR analysis estimates the total causal effect of exposure to one trait of interest on a particular disease outcome. In contrast, multivariable MR analysis can be used to investigate the effects of multiple traits on the outcome. [4] This method is useful if several exposures are associated with each other, or if one particular exposure is believed to mediate the effects of related exposures on the outcome. [5]

##

## **1.2 Genome-wide association studies (GWAS) used for Mendelian randomisation (MR) analysis**

The following information is summarised in Table S1.

### 1.2.1 GWAS Of Late Onset Alzheimer’s Disease

Summary data from a meta-analysis of GWAS of late-onset Alzheimer’s Disease (AD) that was undertaken by the International Genomics of Alzheimer’s Project (IGAP) were available from four consortia through the IEU Open GWAS project. [6] The 4 consortia were the Alzheimer Disease Genetic Consortium (ADGC), Cohorts for Heart and Aging Research in Genomic Epidemiology (CHARGE), the European Alzheimer’s Disease Initiative (EADI), and Genetic and Environmental Risk in AD (GERD). [7] In total, there were 63,926 non-Hispanic white (NHW) participants in this meta-analysis, which included 21,982 AD cases and 41,944 cognitively normal controls. AD cases were confirmed clinically or by autopsy. The participants were selected from 46 case-control studies performed by the four consortia; additional information about these case-control studies is reported elsewhere. [7] The appropriate institutional review boards (IRBs) approved the study protocols for all case-control studies. The participants were recruited at various time points and provided written consent before enrolment. Standard quality control was carried out for all individual datasets and following defined exclusion and inclusion criteria before meta-analysis. Genotyping data were imputed for discovery datasets using the 1000 Genomes project and SNPs were excluded if MAF<0.01 and information score <0.40. The genotype dosages were analyzed for each of the studies and then combined in meta-analysis with adjustment for age, sex and principal components. This Stage 1 discovery meta-analysis of AD identified 12 genome-wide significance loci (p<5x10^-8^), which are previously known. After that, Stage 2 meta-analysis was carried out using the I-select chip developed by Lambert *et al*. [8] and with the combination of stage 1 and stage II, overall meta-analysis identified 21 genome-wide significance loci (p<5x10^-8^).

### 1.1.2 GWAS of retinal structure and vasculature

The UK Biobank recruited ~500,000 participants who were 40-69 years old at enrolment between 2006 and 2010 and were registered with the National Health Service (NHS). At 22 assessment centres across the UK, the participants completed baseline questionnaires on their health, undertook a variety of physical measurements, and several biological samples were collected, including blood for genotyping. [9] An ophthalmic assessment was introduced in 2009 at six assessment centres after the original baseline assessments had started. Spectral-domain OCT devices (3D OCT-1000 Mark II, Topcon, Japan) were used to capture colour fundus images and OCT images of both eyes of the participants between 2009-2010 and 2012-2013. A raster scan protocol of 512 A-scans by 128-B scans captured the 3D volume of the macula in an area of 6mm x 6 mm centred on the fovea. [10] Automated analysis of retinal thickness was conducted with Topcon Advanced Boundary Segmentation (TABS^TM^) software that automatically segments the inner and outer retinal boundaries and sublayers of the retina. [11] Additionally, the software quantifies image quality with indices that include image quality score, internal limiting membrane (ILM), valid count and motion indicators to identify blinks, segmentation errors, and eye motion artefacts.

Since the acquisition of this fundal imaging data, several GWAS have investigated the thickness of the macula and its individual layers and the morphology of the retinal vasculature, as described in the following sections.

#### **1.1.2.1 Macular thickness**

A GWAS of macular thickness considered the OCT images of 81,807 self-reported white participants in the UK Biobank who had an ophthalmic assessment between 2009-2010 or 2012-2013. [12] Macular thickness was reported as the average macular thickness in micrometres (μm) within an area (diameter = 6mm) corresponding with the Early Treatment Diabetic Retinopathy Study (ETDRS) regions of the eye. In the GWAS, the average macular thickness of both eyes was used, if available, after removing outliers and images with quality scores less than 45. Additional quality control parameters were applied to imputed genetic data in the UK Biobank to exclude variants with MAF<0.1, info score <0.3, and missing rate >0.1. These quality controls on the phenotype and genotype meant that data from 68,423 participants of European ancestry were used for the final macular thickness GWAS. Based on relatedness, study participants were subdivided into two groups: unrelated participants (n=59,841) were used in discovery analysis and related participants (n=8609) in the replication analysis. Firstly, an additive genetic mixed model was used to measure the associations between macular thickness and imputed genetic variants by adjusting age, sex, genotyping array, study centre, refraction and the first 10 principal components in the discovery data. Then, the genetic variants with p-values less than 5x10^-8^ in the discovery dataset were analysed again in the replication data to check their effect sizes and directions were consistent. This GWAS identified 139 independent loci, most of which were highly expressed in the retina.

#### **1.1.2.2 Inner retinal layers**

The GWAS of macular retinal nerve fibre layer thickness (mRNFL) and ganglion cell inner plexiform layer thickness (mGCIPL) considered the OCT imaging of 67,713 UK Biobank participants who had undergone the ophthalmic assessment between 2009-2010. [13] The RNFL and GCIPL are the first two inner retinal layers in the macula region of the eye and were measured in micrometres (μm). A total of 31,434 European participants met the inclusion and exclusion criteria and stringent image quality control measures and were included in the GWAS. [13] The GWAS used an additive linear model while factoring age, sex, height, weight, ID of the OCT scan machine, and the first 20 genotyped principal components in the analysis. The analyses were then replicated with two external datasets, the Raine Study and the Rotterdam study, and the results were combined in a meta-analysis that identified 46 loci associated with RNFL or GCIPL thickness in the study.

#### **1.1.2.3 Outer retinal layers**

Three GWAS were conducted by Currant *et al* to identify genetic variants associated with mean thickness of each of the outer retinal layers: Outer nuclear layer (ONL), inner segment (IS) and outer segment (OS) layers. These quantitative phenotypes were derived from the OCT images within UK Biobank cohort. [14] Genotyping and phenotyping quality controls were applied, which included removing participants with OCT image quality scores <45, excluding the poorest 20% of the images based on their segmentation indices (inner limiting membrane (ILM), valid count, minimum motion correlation, maximum motion delta, and maximum motion factor), and removing outliers in refractive error. The final data set included 31,135 White British individuals for genomic modelling. Additive linear models were applied with adjustments for: age, weight, sex, height, OCT machine, and the first 20 genotypic PCs. Next, the summary data for each of the GWAS were meta-analyzed by multi-trait analysis of GWAS (MTAG). From the three independent GWAS, a total of 111 loci were identified as significantly associated with the thickness of at least one of the outer retinal layers.

#### **1.1.2.4 Vessel width and tortuosity**

Colour photos taken by the SD-OCT device (3D OCT-1000) used in the UK Biobank study were centred on the macula and included the optic disc. Anatomical features of the retinal vasculature (arterioles and venules) were automatically identified from the retinal images using the QUARTZ system. [15] After that, the tortuosity of the retinal blood vessels was measured in microns in each image of retina and weighted by the length of the vessel segments (in microns); arteriolar and venular vessels were analysed separately. [15] Subsequently, the mean arteriolar and venular tortuosity for both eyes of the participants were summarized in arbitrary units to conduct the GWAS. [15] The mean width of the retinal arteriolar and venular blood vessels was measured in microns to perform GWAS of venular width and arteriolar width. [15] Next, 4 separate GWAS were performed of arteriolar tortuosity, venular tortuosity, arteriolar width, and venular width based on imaging data from 52,798 individuals of European ancestry in UK Biobank. The GWAS were conducted with Bolt-LMM software and the additive genetic effect of each SNP was adjusted for age, sex, spherical equivalent and the first ten principal components. The study identified 119 loci associated with these vasculature phenotypes: 89 loci were associated with arteriolar tortuosity, 17 loci with venular tortuosity, 2 with arteriolar width, and 11 loci associated with venular width. AT had the highest SNP heritability (h^2^=0.51) among the retinal vasculature traits.

All 4 vascular phenotypes were considered in this study, but as there were only 2 SNPs associated with arteriolar width, this trait was not included in our MR analyses because not all MR methods could be applied.

#### **1.1.2.5 Retinal vessel fractal dimension**

The fractal dimension of retinal vasculature is a measure of its complexity. Based on the colour fundus images of UK Biobank participants, Villaplana-Velasco et al conducted a GWAS of retinal vessel fractal dimension. [16] The study used the QUARTZ system and a semi-automatic algorithm developed in VAMPIRE software to create a binary vessel map from 175,611 colour fundus images from 67,725 UK Biobank participants. After exclusions based on image quality score (IQS) (a score based on imaging artefacts, connectivity, and the sharpness of the binary vessel maps), 98,603 images were classified as good quality (49,903 from the right eye and 48,700 from the left eye). The fractal dimension was measured from these good-quality colour fundus images by applying the multifractal analysis method [17] in VAMPIRE software. [16] The left and right eye fractal dimension values were compared when the images of both eyes of the participants passed the quality control process and there was asymmetry in fractal dimensions between the eyes. Next, separate GWAS of left eye, and right eye, and mean fractal dimensions were conducted based on the data from ~38,000 unrelated individuals with white British ancestry in UK Biobank. [16] Genetic data from these unrelated participants were included in the analysis if HWE >10^-6^, MAF >5x10^-3^, call rate >0.9, kinship <0.0442 and imputed score >0.9. Genome-wide associations between fractal dimension and each SNP were determined using a linear regression model, adjusting for age, sex, IQS, assessment centre, genotyping batch, and the first 10 principal components. The GWAS result for mean fractal dimension was analogous to the GWAS of left and right eye fractal measures and identified three genes (OCA2, HERC2 and IRF4) that were significantly associated with retinal fractal dimension.

### 1.1.3 GWAS of optic disc morphology

Optic disc morphology can be defined by optic disc area, optic cup area and the vertical cup-to-disc ratio VCDR. A GWAS meta-analysis was conducted of these traits by the International Glaucoma Genetics Consortium (IGGC), based on the results of 19 studies of individuals with European ancestry who underwent an ophthalmic examination: 18 studies contributed to the meta-analysis of VCDR, and 16 studies to the meta-analysis of optic cup area and optic disc area. [18] The phenotyping methods for optic nerve head measurement varied across the individual studies; for instance, the Brisbane Adolescent Twins Study (BATS) and Twins Eye Study in Tasmania (TEST) used the Nidek 3-Dx fundus camera (Nidek, Gamagori, Japan) to capture simultaneous stereoscopic images of the optic disc that were digitalized using a Nikon CoolScan IV ED slide scanner before the images were magnified using participants’ refraction and keratometry data and analyzed using SterioDx software (StereoGraphics Corp., Beverly Hills, CA, USA). Other studies included in this GWAS used a variety of other imaging techniques, including Heidelberg Retinal Tomography and indirect ophthalmoscopy, that are described in more detail in the supplementary material. [18] Overall, the study used the 1000 Genomes phase 1 imputation reference samples and excluded SNPs with MAF<0.01, imputation score <0.3 or R^2^<0.3 leaving 23,899 individuals in the final meta-analysis of VCDR, 22,504 individuals in the analysis of optic disc area, and 22,489 individuals in the analysis of optic cup area. In all IGCC cohorts, OCA was adjusted for ODA (since larger optic discs tend to have larger physiological cups). The genetic models were adjusted for age, sex and the first five principal components.

### 1.1.4 GWAS of refractive error and axial length

#### **1.1.4.1 Refractive error**

Genetic associations with refractive error (RE) were investigated in a two-stage GWAS meta-analysis of 542,934 European participants from the following cohorts: UK Biobank, 23andMe, the Genetic Epidemiology Research on Adult Health and Ageing (GERA) and Consortium for Refractive Error and Myopia (CREAM). [19] RE was defined by spherical equivalent=spherical power + (cylinder power) / 2. In the first (discovery) stage, two independent GWAS were conducted based on data from 102,117 UK Biobank participants: the first GWAS of directly measured spherical equivalent and the second GWAS of the probability of myopia, then their summary statistics were combined. Subsequently, two additional GWAS were performed of the spherical equivalent of 34,998 individuals from the GERA cohort, and self-reported myopia from the 23andMe cohort that comprised 106,086 cases and 85,757 controls. In the final meta-analysis, all available summary statistics were pooled together with a previously reported GWAS from the CREAM cohort. All the GWAS analyses were adjusted for age, sex and major principal components. The study identified 336 novel genetic loci associated with RE and reported that these genetically associated variants explained 18.4% of the heritability of RE.

#### **1.1.4.2 Axial length**

Axial length (AL) is a measurement of eye length from the surface of the cornea to the retinal pigment epithelium of the eye; it is strongly associated with refractive error. A GWAS of AL was conducted based on 16,523 non-Hispanic white participants from the GERA cohort who had at least one recorded AL measurement of both eyes. [20] Linear regression was performed on the mean AL of the participants’ eyes, adjusting for the following covariates: age, sex and ancestry principal components. The analysis identified 16 genomic loci, including 5 novel SNPs, that were associated with AL with genome-wide significance. The associations between the 5 novel SNPs and AL were replicated in a subset of the CREAM cohort of European ancestry.

## **1.2 Selection of genetic instruments for Mendelian randomisation analyses**

The pipeline for selecting SNPs to use as genetic instruments in each MR analysis is summarised in Figure S3. First, genetic variants associated with each exposure at genome-wide significance (p<5x10^-8^) were identified from GWAS summary data. Next, clumping was applied using a European population reference (1000 genomes, clumping window 10,000 kb, r^2^<0.001) to retain independent variants with the lowest p-values. Then, after extracting the selected genetic variants from the outcome GWAS summary statistics, exposure and outcome datasets were harmonized to ensure the correct orientation of alleles and palindromic SNPs were removed. In addition, Steiger filtering was applied to remove the genetic instruments with a stronger correlation with the outcome than the exposure and to check the sensitivity of the main MR findings. [2] This pipeline was followed in the selection of genetic instruments for all exposures and outcomes in the MR analyses; further details are provided in the following sections and summarised in Supplementary Figures S4 & S5. To make effective decisions on the inclusion of palindromic SNPs in the MR study and performing Steiger filtering, a few GWAS summary data, e.g. AD, FD, ODA, OCA and VCDR had missing effect allele frequency, therefore, we obtained the allele frequency information from the 1000 Genomes project for the European population.

### 1.2.1 Alzheimer’s Disease

In a GWAS meta-analysis, there were 21 SNPs independently associated with AD that were identified in a combined meta-analysis (phase 1 and phase 2) and met the p-value threshold (p<5x10^-8^), reported in (<https://www.nature.com/articles/s41588-019-0358-2/tables/1>). [7] First, summary data for these reported 21 SNPs were extracted from the IEU OpenGWAS project ( ID: ieu-b-2) (Supplementary Table S2). Next, SNP-outcome estimates were extracted from the GWAS summary data for each of the ocular features. For some ocular features, 1-2 SNPs were excluded at this stage because they were not available in the outcome summary statistics (Supplementary Figure S3). No SNPs were excluded during harmonization. To estimate the causal effect of AD on each of the ocular features, there were 18 SNPs available for FD; 19 SNPs for ODA, OCA, VCDR; 21 SNPs for AT, VT and VW, respectively, and 20 SNPs remained for the rest of the ocular features. For further sensitivity analysis, some SNPs were removed by Steiger filtering because they had stronger associations with the outcomes than the exposure (Supplementary Figure S4).

### 1.2.2 Macular thickness

A GWAS identified 19,062 SNPs associated with macular thickness (MT) below the genome-wide threshold of p<5x10^-8^ shared by the author. [12] Of these, 112 were independently associated with MT after clumping. The effect sizes, standard errors and p-values of 108 SNPs were available in the outcome (AD) GWAS summary data for MR analysis, including proxies for 15 SNPs. Three palindromic SNPs were excluded during harmonization. The Steiger test confirmed the direction of causality for all 105 remaining SNPs was correct in the MR analysis (Supplementary Figure S5 & Supplementary Table S3).

### 1.2.3 Retinal nerve fibre layer thickness

Summary data from the GWAS of mRNFL thickness [13] was available online through the GWAS Catalog (<https://www.ebi.ac.uk/gwas/efotraits/OBA_2050111>https://www.ebi.ac.uk/gwas/efotraits/OBA_2050111). Of 9,231,066 SNPs that were extracted from the summary statistics, there were 2,604 genetic variants that were associated with RNFL with GWAS significance (p<5x10^-8^). After clumping, there were 25 genetic variants that were independently associated with RNFL thickness; the effects of 21 SNPs on the outcome (AD) were directly extracted from the outcome summary statistics, proxies were used for three SNPs, one unmatched variant was removed, and no palindromic variant was excluded during the harmonization process. The Steiger test confirmed the direction of the remaining 24 genetic variants was correct in the MR analysis (Supplementary Figure S5 & Supplementary Table S4).

### 1.2.4 Ganglion cell inner plexiform layer thickness

Summary data from the GWAS of mGCIPL thickness [13] was available from the GWAS Catalog (<https://www.ebi.ac.uk/gwas/efotraits/OBA_2050104>). From 9,231,066 genetic variants, there were 1661 SNPs associated with GCIPL thickness with p<5x10^-8^. During clumping, 1639 SNPs were removed due to linkage disequilibrium (LD) with other variants or because they were not found in the European reference panel. Of the remaining 22 genetic variants, 20 SNPs were directly found in the outcome summary data and proxies were used for 2 SNPs. One palindromic SNP was removed during harmonization. The Steiger test confirmed that the final 21 SNPs were associated with the exposure > outcome in the MR analysis (Supplementary Figure S5 & Supplementary Table S5).

### 1.2.5 Outer nuclear layer thickness

Summary data from the GWAS of ONL thickness [14] was available from the GWAS Catalogue (<https://www.ebi.ac.uk/gwas/efotraits/EFO_0803371>). Among 9,231,216 SNPs, there were 8,991 SNPs associated with ONL thickness with GWAS significance (p<5x10^-8^). After clumping, there were 61 SNPs that were independently associated with ONL, and 58 SNPs remained after harmonisation and the removal of palindromic SNPs. Steiger filtering confirmed that the direction of causality of these SNPs was correct in the MR analysis (Supplementary Figure S5 & Supplementary Table S6).

### 1.2.6 Inner segment layer thickness

Summary data from the GWAS of IS thickness [14] was available from the GWAS Catalog (<https://www.ebi.ac.uk/gwas/efotraits/EFO_0803372>). There were 1,234 SNPs associated with IS thickness below the GWAS threshold for significance. However, only 15 SNPs were independently associated with IS thickness after clumping. No SNPs were removed during harmonization. Steiger filtering confirmed the direction of effect of these 15 SNPs was correct in the MR analysis (Supplementary Figure S5 & Supplementary Table S7).

### 1.2.7 Outer segment layer thickness

Currant *et al.* also reported the results of their GWAS of OS thickness [14] , and the summary data was available from the GWAS Catalog (<https://www.ebi.ac.uk/gwas/efotraits/EFO_0803373>). There were 36 SNPs that were independently associated with OS thickness below the genome-wide significance threshold, and 35 of them were available in the outcome summary statistics. There were 2 palindromic SNPs removed during harmonization and the Steiger filtering test confirmed the causal direction of effect of the remaining 33 SNPs was correct in the MR analysis (Supplementary Figure S5 & Supplementary Table S8).

### 1.2.8 Arteriolar tortuosity

A GWAS of retinal arteriolar tortuosity identified 10,308 SNPs associated with arteriolar tortuosity below the GWAS significance threshold shared by authors, [15] but only 89 SNPs remained after clumping for independently associated SNPs. There were 86 SNPs available in the outcome summary statistics: 75 were identified directly, proxies were found for 11 SNPs; and three unmatched SNPs were removed. Four palindromic SNPs were removed during harmonization. Finally, the Steiger test confirmed the 82 remaining SNPs were acting in the appropriate direction in the MR analysis (Supplementary Figure S5 & Supplementary Table S9).

### 1.2.9 Venular tortuosity

A GWAS summary data of VT was shared by authors [15] where there were 1,652 SNPs associated with VT that met the GWAS significance threshold, and 16 independently associated SNPs remained after clumping. No palindromic SNPs were removed during harmonization. Steiger filtering removed one SNP that was acting in the incorrect direction in the MR analysis (Supplementary Figure S5 & Supplementary Table S10).

### 1.2.10 Venular width

A GWAS of VT was performed by Jiang *et al*., [15] and the summary data were shared by the authors. There were 995 SNPs associated with venular width below the GWAS threshold for significance. However, 982 variants were removed during clumping using the European population reference. Of the remaining 13 SNPs, there were matched data for 12 SNPs in the outcome summary statistics, and a proxy was found for one SNP. No SNPs were removed during harmonization. The remaining 13 SNPs were acting in the correct direction in the MR analysis according to the Steiger test (Supplementary Figure S5 & Supplementary Table S11).

### 1.2.11 Retinal vessel fractal dimension

In a GWAS of retinal vessel fractal dimension, [16] shared by the authors, 306 SNPs were associated with the phenotype below the GWAS significance threshold. However, only four genetic variants were found to be independently associated with fractal dimension after using the European population reference for clumping. Data was available for the 4 SNPs in the outcome summary statistics. Harmonisation removed one palindromic SNP (rs16891982). The Steiger test suggested all three remaining SNPs were associated with the outcome > exposure. Therefore, no SNPs remained to perform further sensitivity analysis. (Supplementary Figure S5 & Supplementary Table S12).

### 1.2.12 Optic disc area

Summary data from the GWAS of ODA was available from the GWAS Catalog (<https://www.ebi.ac.uk/gwas/studies/GCST004076>). [18] Based on this GWAS of optic disc area, 12 SNPs were found to be independently associated with the phenotype below the GWAS significance threshold. One palindromic SNP (rs5762752) was removed during harmonization. Steiger filtering confirmed all 11 SNPs were acting in the correct causal direction in the MR analysis (Supplementary Figure S5 & Supplementary Table S13).

### 1.2.13 Optic cup area

Summary data from the GWAS of OCA was available from the GWAS Catalog (<https://www.ebi.ac.uk/gwas/studies/GCST004137>). [18] This GWAS identified 50 genome-wide significance SNPs that were independently associated with optic cup area. Of these 50 SNPs, 34 SNPs were identified directly in the outcome summary statistics, proxies were used for 9 SNPs and the remaining 7 SNPs were unmatched and consequently removed. Three additional palindromic SNPs (rs1346, rs4820791, rs76230764) were excluded during harmonization. There were 40 SNPs that were used in the MR analysis and the Steiger test confirmed they were all acting in the correct direction of causality (Supplementary Figure S5 & Supplementary Table S14).

### 1.2.14 Vertical cup:disc ratio

Summary data from the GWAS of VCDR were available from the GWAS Catalog (<https://www.ebi.ac.uk/gwas/studies/GCST004075>). [18] From this GWAS of vertical cup:disc ratio, there were 24 SNPs that were independently associated with VCDR below the GWAS significance threshold that remained after clumping using the European population reference. However, one of these SNPs was excluded as it remained unidentified in the AD GWAS, so the SNP-outcome estimates for all 23 SNPs were extracted using the same source. Six palindromic SNPs were excluded; thus, all 17 SNPs were available for final MR analysis. One SNPs were removed from the Steiger filtering for the additional sensitivity analysis (Supplementary Figure S5 & Supplementary Table S15).

## **1.3 Selection of genetic instruments for multivariable Mendelian randomization analyses**

Multivariable Mendelian randomization was applied using the summary-level genetic data from the GWAS of optic disc area, refractive error and axial length. As described in Section 1.2.12, we found 12 SNPs were associated with optic disc area with genome-wide significance. The genetic instruments for refractive error and axial length were selected as follows:

### 1.3.1 refractive error

Summary-level data for refractive error were obtained from the publicly available GWAS Catalog (<https://www.ebi.ac.uk/gwas/studies/GCST010003>). [19] From a list of 8,754,054 genomic loci, 294 SNPs were identified that were associated with refractive error with genome-wide significance and remained after applying clumping using the European population as a reference panel. The F statistics for these variants were between 29 and 990 (Supplementary Table S16).

### 1.3.2 axial length

A GWAS of axial length provided summary data for 9,316,693 genomic loci. [20] Among these variants, a total of 381 SNPs were associated with axial length and met the GWAS significance threshold; after clumping, 15 SNPs remained that were independently associated with AL (Supplementary Table S17).

### 1.3.3 Combined selection of genetic instruments for MVMR

In the MVMR analysis, a total of 321 SNPs were used as genetic instruments after merging the SNPs that were independently associated with optic disc area, refractive error and axial length with genome-wide significance. The gene-exposure summary data were obtained for all the included SNPs from each of the individual GWAS. After reapplying the clumping, gene-outcome associations were obtained from the summary data of the outcome of interest (AD). The harmonization process yielded a total of 257 SNPs after removing the palindromic SNPs. After formatting the dataset for the MVMR package [21] and multivariable MR analysis was applied with the genetic covariance set equal to zero.

# Supplementary Figures

## **Figure S1. GWAS summary data used in the Mendelian randomization analyses of this study.**

(A) Alzheimer’s Disease study cohorts; (B) Cohorts for ocular traits. **Abbreviations:** ADGC=Alzheimer’s Disease Genetic Consortium, CHARGE=Cohorts for Heart and Aging Research in Genomic Epidemiology, EADI=The European Alzheimer’s Disease Initiative, GERAD=Genetic and Environmental Risk in AD, mRNFL=macular Retinal Nerve Fibre Layer, mGCIPL=macular Ganglion Cell Inner Plexiform Layer, MT=Macular thickness, ONL= Outer Nuclear Layer, IS= Inner Segment Layer, OS= Outer Segment Layer,  AT=Arteriolar Tortuosity, VT=Venular Tortuosity, VW=Venular Width, FD = Fractal Dimension, VCDR=Vertical Disc Cup Ratio, ODA= Optic Disc Area, OCA= Optic Cup Area.

## **Figure S2. Visual representation of the possible causal relationships between AD and the ocular traits that are tested in this study.**

Genetic variants associated with an exposure, Alzheimer's Disease (AD), are used as proxies to estimate the causal effect of the exposure on the outcome of interest, e.g., retinal nerve fiber layer (RNFL) thickness. The Mendelian randomization (MR) analysis is also repeated in the reverse direction to estimate the effect of RNFL thickness on AD risk. These analyses are based on three assumptions: (1) the genetic instruments are truly associated with the exposure (2); there are no variant-confounder associations (depicted by dashed arrow with a red cross); (3) the variants do not influence the outcome via different biological pathways from the exposure under investigation (depicted by dashed arrow with red cross) known as horizontal pleiotropy. SNPs that act via vertically pleiotropic pathways do not invalidate the assumptions of MR.

## **Figure S****3. Pipeline for defining genetic instruments**

## **Figure S4*.* Pipeline for genetic instrument selection for *Alzheimer’s Disease***

Abbreviations: MT = Macular Thickness, RNFL = Retinal Nerve Fibre Layer, GCIPL = Ganglion Cell Inner Plexiform Layer, ONL = outer nuclear layer, IS = inner segment layer thickness, OS = outer segment layer, ODA = optic disc area, OCA = optic cup area, VCDR = vertical cup disc ratio, AT = Arteriolar Tortuosity, VT = Venular Tortuosity, VW = Venular Width, and FD = retinal vessel fractal dimension.

## **Figure S5. Pipelines for genetic instrument selection for each of the ocular traits**

Abbreviations: MT= Macular Thickness, RNFL=Retinal Nerve Fibre Layer, GCIPL=Ganglion Cell Inner Plexiform Layer, ONL=outer nuclear layer, IS=inner segment layer thickness, OS=outer segment layer, ODA=optic disc area, OCA=optic cup area, VCDR=vertical cup disc ratio, AT=Arteriolar Tortuosity, VT=Venular Tortuosity, VW=Venular Width, and FD=retinal vessel fractal dimension.

## **Figure S6. Scatter plots showing the causal relationships between Alzheimer's disease and selected ocular traits.**

The plots represent the results of the main and sensitivity MR analyses to estimate the causal effects of the genetic risk of AD on: (A) arteriolar tortuosity (AT); (B) macular retinal fibre layer (mRNFL); (C) macular ganglion cell inner plexiform layer; (D) outer segment (OS) layer thickness; (F) optic disc area (ODA); whereas plot (E) represents the results of MR analyses to estimate the causal effects of the genetical liability of optic disc area on AD. In plots A-D & F, the x-axis represents the effect of the genetic variant on AD, and the y-axis represents the effects of the same genetic instruments on the AT, mRNFL, mGCIPL, OS, and ODA, respectively. In plot E, the x-axis represents the effects of the genetic variants on ODA, and the y-axis represents the effects of the same genetic instruments on AD. The regression lines represent the different MR methods, i.e., inverse variance weighted (light blue), MR-Egger (dark blue), weighted median (light green) and weighted mode (dark green).

## **Figure S7. Forest plot summarising the causal associations between AD and the ocular traits in sensitivity analyses based on summary data from 3 other independent GWAS of AD**

**Abbreviations:** AD=Alzheimer’s disease, IVW = inverse weighted variance, MT= Macular Thickness, mRNFL=macular Retinal Nerve Fibre Layer, mGCIPL=macular Ganglion Cell Inner Plexiform Layer, ONL=outer nuclear layer, IS=inner segment layer thickness, OS=outer segment layer, ODA=optic disc area, OCA=optic cup area, VCDR=vertical cup disc ratio, AT=Arteriolar Tortuosity, VT=Venular Tortuosity, VW=Venular Width, and FD=retinal vessel fractal dimension.

## **Figure S8: Mendelian Randomization leave-one-out sensitivity analysis of the causal effect of AD on AT based on the summary data of four independent GWAS of AD.**

MR leave-one-out plots summarising the causal effect of AD on AT by iteratively removing each SNP from the summary data of significantly associated SNPs identified in the genome-wide association studies (GWAS) of AD derived by (A) Kunkle et al [7] (B) Lambert et al [8] (C) Jansen et al [22], and (D) Bellenguez et al. [23] Overall estimates of the causal effect are shown in red, and the error bars represent the 95% confidence interval.

**Abbreviations:** AD=Alzheimer’s disease, AT=Arteriolar Tortuosity, SNP= single nucleotide polymorphism

## **Figure S9: Mendelian Randomization leave-one-out sensitivity analysis of the causal effect of AD on mRNFL thickness based on the summary data of four independent GWAS of AD.**

MR leave-one-out plots summarising the causal effect of AD on mRNFL thickness by iteratively removing each SNP from the summary data of significantly associated SNPs identified in the genome-wide association studies (GWAS) of AD derived by (A) Kunkle et al [7] (B) Lambert et al [8] (C) Jansen et al [22], and (D) Bellenguez et al. [23] Overall estimates of the causal effect are shown in red, and the error bars represent the 95% confidence interval.

**Abbreviations:** AD=Alzheimer’s disease, mRNFL=macular Retinal Nerve Fibre Layer, SNP= single nucleotide polymorphism.

## **Figure S10: Mendelian Randomization leave-one-out sensitivity analysis of the causal effect of AD on mGCIPL thickness based on the summary data of four independent GWAS of AD.**

MR leave-one-out plots summarising the causal effect of AD on mGCIPL thickness by iteratively removing each SNP from the summary data of significantly associated SNPs identified in the genome-wide association studies (GWAS) of AD derived by (A) Kunkle et al [7] (B) Lambert et al [8] (C) Jansen et al [22], and (D) Bellenguez et al. [23] Overall estimates of the causal effect are shown in red, and the error bars represent the 95% confidence interval.

**Abbreviations:** AD=Alzheimer’s disease, mGCIPL=macular Ganglion Cell Inner Plexiform Layer, SNP= single nucleotide polymorphism.

## **Figure S11: Mendelian Randomization leave-one-out sensitivity analysis of the causal effect of AD on OS layer thickness based on the summary data of four independent GWAS of AD.**

MR leave-one-out plots summarising the causal effect of AD on OS layer thickness by iteratively removing each SNP from the summary data of significantly associated SNPs identified in the genome-wide association studies (GWAS) of AD derived by (A) Kunkle et al [7] (B) Lambert et al [8] (C) Jansen et al [22], and (D) Bellenguez et al. [23] Overall estimates of the causal effect are shown in red, and the error bars represent the 95% confidence interval.

**Abbreviations:** AD=Alzheimer’s disease, OS=outer segment, SNP= single nucleotide polymorphism

## **Figure S12: Mendelian Randomization leave-one-out sensitivity analysis of the causal effect of AD on ODA based on the summary data of four independent GWAS of AD.**

MR leave-one-out plots summarising the causal effect of AD on ODA by iteratively removing each SNP from the summary data of significantly associated SNPs identified in the genome-wide association studies (GWAS) of AD derived by (A) Kunkle et al [7] (B) Lambert et al [8] (C) Jansen et al [22], and (D) Bellenguez et al. [23] Overall estimates of the causal effect are shown in red, and the error bars represent the 95% confidence interval.

**Abbreviations:** AD=Alzheimer’s disease, ODA=optic disc area, SNP= single nucleotide polymorphism.

# Supplementary Tables

## **Table S1: Summary of GWAS data sources**

| **Trait** | **Authors** | **Population** | **Cohort** | **Date** | **Eligibility criteria** | **Selection method** | **Sample size** | **Measurement** | **Assessment method** |
| --- | --- | --- | --- | --- | --- | --- | --- | --- | --- |
| AD | Kunkle et al [7] | European | ADGC, CHARGE, EADI, GERAD | Various time points | Aged between 60-100 years | Participants were selected from 46 case control studies. | 21,982 cases  41,499 controls | Case and control | AD cases were ascertained clinically or by autopsy |
| MT | Gao et al [12] | European | UK Biobank | 2006-2010 | NHS registered & aged 40-69 | Voluntary recruitment. | 68,423 | micrometres (μm) | 3D OCT-1000 Mark II Topcon device and TABS^TM^ software |
| mRNFL | Currant et al [13] | European | UK Biobank | 2006-2010 | NHS registered & aged 40-69 years | Voluntary recruitment. | 31,434 | micrometres (μm) | 3D OCT-1000 Mark II Topcon device and Advanced Boundary Segmentation (TABS^TM^) software |
| mGCIPL | Currant et al [13] | European | UK Biobank | 2006-2010 | NHS registered & aged 40-69 years | Voluntary recruitment. | 31,434 | micrometres (μm) | 3D OCT-1000 Mark II Topcon device and TABS^TM^ software |
| ONL | Currant et al [14] | European | UK Biobank | 2006-2010 | NHS registered & aged 40-69 years | Voluntary recruitment. | 31,135 | micrometres (μm) | 3D OCT-1000 Mark II Topcon device and TABS^TM^ software |
| IS | Currant et al [14] | European | UK Biobank | 2006-2010 | NHS registered & aged 40-69 years | Voluntary recruitment. | 31,135 | micrometres (μm) | 3D OCT-1000 Mark II Topcon device and TABS^TM^ software |
| OS | Currant et al [14] | European | UK Biobank | 2006-2010 | NHS registered & aged 40-69 years | Voluntary recruitment. | 31,135 | micrometres (μm) | 3D OCT-1000 Mark II Topcon device and TABS^TM^ software |
| AT | Jiang et al [15] | European | UK Biobank | 2006-2010 | NHS registered & aged 40-69 years | Voluntary recruitment. | 52,798 | arbitrary unit | Colour fundal photography with 3D OCT-1000 Mark II Topcon device and QUARTZ software |
| VT | Jiang et al [15] | European | UK Biobank | 2006-2010 | NHS registered & aged 40-69 years | Voluntary recruitment. | 52,798 | arbitrary unit | Colour fundal photography with 3D OCT-1000 Mark II Topcon device and QUARTZ software |
| VW | Jiang et al [15] | European | UK Biobank | 2006-2010 | NHS registered & aged 40-69 years | Voluntary recruitment. | 52,798 | micrometres (μm) | Colour fundal photography with 3D OCT-1000 Mark II Topcon device and QUARTZ software |
| FD | Villaplana-Velasco et al [16] | European | UK Biobank | 2006-2010 | NHS registered & aged 40-69 years | Voluntary recruitment. | ~38,000 | micrometres (μm) | Colour fundal photography with 3D OCT-1000 Mark II Topcon device and VAMPIRE software |
| ODA | Springelkamp et al [18] | European | IGGC | Various time point | Age >40 years | Participants were selected from 16 studies | 22,489 | millimetres (mm) | Varied phenotyping methods were used and summarised in Table S1b of Springelkamp et al. [18] |
| OCA | Springelkamp et al [18] | European | IGGC | Various time point | Age >40 years | Participants were selected from 16 studies | 22,504 | millimetres (mm) | Varied phenotyping methods were used and summarised in Table S1b of Springelkamp et al. [18] |
| VCDR | Springelkamp et al [18] | European | IGGC | Various time point | Age >40 years | Participants were selected from 19 studies | 23,899 | ratio | Varied phenotyping methods were used and summarised in Table S1b of Springelkamp et al. [18] |
| AL^*^ | Jiang et al. [20] | European | GERA | Various time point | Age > 18 years | Voluntary recruitment. | 16,523 | millimetres (mm) | Haag-Streit Lenstar 900 device |
| RE^*^ | Hysi et al. [19] | European | UK Biobank  23andME  GERA | Various time point | UK Biobank: aged 40-69 years  GERA: age>18 years | Voluntary recruitment. | 542,934 | diopter | Tomey RC 5000 Auto Refkeratometer, Tomey Corp., Nagoya, Japan |

**Abbreviations:** MT= Macular Thickness, RNFL=Retinal Nerve Fibre Layer, GCIPL=Ganglion Cell Inner Plexiform Layer, ONL=outer nuclear layer, IS=inner segment layer thickness, OS=outer segment layer, AT=Arteriolar Tortuosity, VT=Venular Tortuosity, VW=Venular Width, FD=retinal vessel fractal dimension, ODA=optic disc area, OCA=optic cup area, VCDR=vertical cup disc ratio, AL=axial length, and RE=refractive error, ADGC=Alzheimer’s Disease Genetic Consortium, CHARGE=Cohorts for Heart and Aging Research in Genomic Epidemiology, EADI=The European Alzheimer’s Disease Initiative, GERAD=Genetic and Environmental Risk in A, IGGC= International Glaucoma Genetics Consortium, GERA=Genetic Epidemiology Research on Adults Health and Aging, NHS= National Health Service, OCT= Optical Coherence Tomograph, TABS= Topcon Advanced Boundary Segmentation.

^*^Axial length (AL) and refractive error (RE) were used to perform multivariable Mendelian randomisation only.

## **Table S2. Genetic instruments for Alzheimer’s Disease**

Variants extracted from *Kunkle et al* [7]

| **SNP** | **CHR** | **Position** | **Effect Allele** | **Beta** | **SE** | ***P*-value** | **F-statistic** |
| --- | --- | --- | --- | --- | --- | --- | --- |
| rs4844610 | 1 | 207802552 | A | 0.146 | 0.018 | 8.2E-16 | 67.9 |
| rs6733839 | 2 | 127892810 | T | 0.169 | 0.015 | 4.0E-28 | 120.8 |
| rs10933431 | 2 | 233981912 | C | 0.100 | 0.019 | 2.5E-07 | 47.8 |
| rs9271058 | 6 | 32575406 | A | 0.094 | 0.017 | 5.1E-08 | 33.9 |
| rs75932628 | 6 | 41129252 | T | 0.698 | 0.100 | 2.9E-12 | 30.7 |
| rs9473117 | 6 | 47431284 | A | -0.082 | 0.015 | 2.3E-07 | 31.9 |
| rs12539172 | 7 | 100091795 | T | -0.067 | 0.015 | 2.0E-05 | 71.1 |
| rs10808026 | 7 | 143099133 | A | -0.101 | 0.018 | 3.0E-08 | 37.4 |
| rs73223431 | 8 | 27219987 | T | 0.093 | 0.015 | 8.3E-10 | 29.8 |
| rs9331896 | 8 | 27467686 | T | 0.126 | 0.015 | 3.6E-16 | 68.3 |
| rs7920721 | 10 | 11720308 | A | -0.078 | 0.015 | 1.9E-07 | 65.5 |
| rs11218343 | 11 | 121435587 | T | 0.205 | 0.036 | 2.6E-08 | 42.1 |
| rs3740688 | 11 | 47380340 | T | 0.093 | 0.014 | 9.7E-11 | 33.3 |
| rs7933202 | 11 | 59936926 | A | 0.116 | 0.014 | 2.1E-15 | 44.7 |
| rs3851179 | 11 | 85868640 | T | -0.119 | 0.014 | 5.8E-16 | 234.7 |
| rs17125924 | 14 | 53391680 | A | -0.122 | 0.024 | 6.6E-07 | 466.7 |
| rs12881735 | 14 | 92932828 | T | 0.088 | 0.017 | 4.8E-07 | 40.1 |
| rs138190086 | 17 | 61538148 | A | 0.253 | 0.056 | 7.4E-06 | 44.1 |
| rs3752246 | 19 | 1056492 | C | -0.124 | 0.020 | 6.6E-10 | 962.3 |
| rs429358 | 19 | 45411941 | T | -1.201 | 0.018 | 1.0E-200 | 33.6 |
| rs6024870 | 20 | 54997568 | A | -0.127 | 0.026 | 1.1E-06 | 74.3 |

**Abbreviations:** CHR, chromosome; SE, standard error. Effect estimates are for the late-onset AD.

## **Table S3. Genetic instruments for macular thickness**

Variants extracted from Gao et al [12]

| **SNP** | **CHR** | **Position** | **Effect Allele** | **Beta** | **SE** | ***P*-value** | **F statistic** |
| --- | --- | --- | --- | --- | --- | --- | --- |
| rs113442694 | 1 | 227569107 | A | 0.854 | 0.128 | 1.6E-12 | 44.1 |
| rs11576909 | 1 | 202812654 | T | -0.885 | 0.069 | 1.7E-38 | 163.8 |
| rs12409048 | 1 | 170699149 | G | 0.477 | 0.078 | 2.0E-09 | 37.2 |
| rs28502486 | 1 | 165342002 | C | 0.515 | 0.090 | 4.4E-09 | 32.6 |
| rs55992320 | 1 | 113493415 | T | 0.639 | 0.082 | 5.4E-18 | 60.7 |
| rs74454622 | 1 | 214107008 | C | 1.115 | 0.128 | 1.4E-17 | 75.8 |
| rs9426785 | 1 | 22249589 | A | -0.383 | 0.072 | 5.0E-08 | 28.0 |
| rs1010659 | 2 | 25595397 | C | -0.458 | 0.074 | 4.0E-10 | 38.1 |
| rs11689553 | 2 | 170067947 | C | 0.538 | 0.079 | 3.7E-11 | 45.4 |
| rs13410155 | 2 | 56212234 | A | -0.493 | 0.077 | 6.4E-12 | 40.3 |
| rs34183224 | 2 | 218514964 | G | 0.908 | 0.134 | 4.3E-12 | 45.7 |
| rs4309622 | 2 | 48283919 | G | 0.413 | 0.070 | 5.1E-10 | 34.0 |
| rs6756117 | 2 | 228069010 | A | 0.417 | 0.070 | 2.0E-10 | 35.4 |
| rs7607305 | 2 | 169028254 | T | 0.706 | 0.078 | 5.2E-19 | 80.0 |
| rs1494836 | 3 | 27720363 | C | -0.732 | 0.095 | 7.3E-14 | 58.4 |
| rs17279437 | 3 | 45814094 | G | 1.958 | 0.111 | 1.4E-71 | 309.9 |
| rs2278806 | 3 | 150142260 | A | -0.817 | 0.140 | 1.5E-08 | 33.6 |
| rs35667547 | 3 | 64547477 | G | -0.610 | 0.103 | 1.4E-09 | 34.8 |
| rs10033769 | 4 | 81554507 | C | 0.519 | 0.081 | 8.5E-11 | 40.3 |
| rs12641813 | 4 | 41454320 | A | 0.832 | 0.147 | 5.3E-09 | 32.0 |
| rs2309957 | 4 | 184942348 | C | 0.536 | 0.070 | 1.0E-14 | 57.2 |
| rs4864847 | 4 | 55071953 | G | -0.499 | 0.081 | 7.5E-10 | 37.3 |
| rs13164947 | 5 | 78041976 | C | 0.535 | 0.072 | 9.9E-14 | 54.3 |
| rs13171669 | 5 | 148601243 | A | -0.778 | 0.069 | 6.8E-32 | 125.5 |
| rs17421627 | 5 | 87847586 | T | -2.992 | 0.131 | 5.1E-120 | 521.1 |
| rs2004187 | 5 | 2612747 | A | -0.480 | 0.069 | 1.2E-12 | 47.5 |
| rs2190989 | 5 | 11584342 | T | -0.499 | 0.068 | 1.0E-13 | 52.6 |
| rs30373 | 5 | 55745334 | C | -0.464 | 0.071 | 5.0E-11 | 41.9 |
| rs6894037 | 5 | 173054910 | C | 0.498 | 0.070 | 3.0E-13 | 49.9 |
| rs14314 | 6 | 150141531 | A | -0.615 | 0.073 | 2.5E-19 | 70.8 |
| rs1567168 | 6 | 79536455 | C | -0.423 | 0.068 | 3.7E-11 | 37.7 |
| rs17507554 | 6 | 11394287 | G | -1.589 | 0.158 | 2.7E-25 | 100.5 |
| rs2324447 | 6 | 84300371 | C | -0.416 | 0.069 | 6.9E-10 | 35.7 |
| rs7764472 | 6 | 35479574 | G | -0.541 | 0.097 | 6.4E-09 | 31.0 |
| rs807897 | 6 | 164605713 | C | -0.451 | 0.069 | 4.4E-12 | 42.4 |
| rs9398171 | 6 | 108983527 | C | -0.704 | 0.075 | 5.8E-22 | 86.0 |
| rs9487729 | 6 | 112151975 | T | 0.528 | 0.087 | 1.1E-08 | 36.5 |
| rs12537870 | 7 | 8118915 | G | -0.643 | 0.102 | 3.8E-11 | 39.8 |
| rs12672451 | 7 | 106853817 | C | -0.511 | 0.080 | 2.1E-10 | 40.0 |
| rs12673771 | 7 | 46637249 | T | -0.773 | 0.069 | 1.7E-29 | 122.9 |
| rs12719025 | 7 | 51100190 | A | -1.039 | 0.069 | 1.1E-54 | 222.3 |
| rs187136683 | 7 | 27084648 | A | -0.425 | 0.071 | 1.8E-10 | 35.8 |
| rs2237473 | 7 | 50742937 | G | -0.736 | 0.083 | 4.4E-20 | 78.2 |
| rs3197597 | 7 | 100172858 | C | 0.613 | 0.097 | 6.2E-10 | 39.8 |
| rs7807296 | 7 | 155617259 | T | 0.486 | 0.069 | 7.6E-12 | 48.5 |
| rs617117 | 8 | 109114464 | T | -0.461 | 0.080 | 1.7E-09 | 33.1 |
| rs7812312 | 8 | 96020400 | T | 0.603 | 0.099 | 1.5E-10 | 36.7 |
| rs2787394 | 9 | 103007414 | C | 0.621 | 0.070 | 3.0E-19 | 78.4 |
| rs28491365 | 9 | 98276374 | T | 0.478 | 0.083 | 6.8E-09 | 33.1 |
| rs543070 | 9 | 126596547 | A | 0.773 | 0.101 | 2.9E-15 | 58.0 |
| rs62567129 | 9 | 71789104 | G | 0.445 | 0.084 | 3.4E-08 | 27.8 |
| rs679038 | 9 | 22029080 | G | -0.442 | 0.069 | 1.3E-10 | 40.1 |
| rs7033598 | 9 | 21565674 | C | 0.719 | 0.072 | 8.2E-26 | 99.6 |
| rs7853479 | 9 | 77121684 | G | 0.500 | 0.072 | 3.3E-13 | 47.4 |
| rs1947075 | 10 | 49741135 | C | 0.701 | 0.072 | 8.8E-24 | 95.0 |
| rs2579136 | 10 | 79619461 | G | 0.436 | 0.076 | 3.6E-10 | 32.9 |
| rs4838578 | 10 | 49410677 | C | -0.517 | 0.081 | 6.8E-12 | 40.1 |
| rs735877 | 10 | 102104521 | C | -0.418 | 0.071 | 2.2E-10 | 34.6 |
| rs786391 | 10 | 28609690 | A | -0.472 | 0.079 | 1.8E-09 | 35.5 |
| rs1042602 | 11 | 88911696 | C | 0.574 | 0.071 | 1.2E-15 | 65.3 |
| rs10893946 | 11 | 128862342 | C | -0.375 | 0.070 | 1.6E-08 | 28.5 |
| rs11606813 | 11 | 69018588 | C | 1.537 | 0.168 | 1.9E-22 | 83.0 |
| rs1255529 | 11 | 95299637 | G | 0.390 | 0.069 | 6.6E-09 | 31.7 |
| rs3802849 | 11 | 112912430 | T | -0.444 | 0.079 | 3.9E-08 | 31.6 |
| rs4936637 | 11 | 121447188 | T | -0.631 | 0.082 | 8.3E-16 | 58.3 |
| rs61878480 | 11 | 31525712 | T | 0.536 | 0.074 | 5.1E-12 | 52.5 |
| rs7127818 | 11 | 17004090 | G | -0.636 | 0.075 | 6.6E-19 | 70.3 |
| rs3138142 | 12 | 56115585 | C | -1.693 | 0.080 | 9.2E-105 | 445.7 |
| rs55971426 | 12 | 96204536 | A | 1.084 | 0.087 | 1.5E-37 | 154.3 |
| rs61955214 | 12 | 123482424 | T | 0.470 | 0.074 | 4.0E-10 | 40.3 |
| rs7985441 | 13 | 111893413 | C | 0.462 | 0.070 | 8.8E-12 | 43.0 |
| rs10220706 | 14 | 60800986 | A | 0.508 | 0.075 | 5.0E-12 | 45.9 |
| rs3811183 | 14 | 23452128 | C | 0.419 | 0.070 | 7.4E-10 | 35.4 |
| rs78042854 | 14 | 73924760 | C | -1.010 | 0.171 | 3.8E-10 | 34.9 |
| rs8008961 | 14 | 68752643 | C | 0.605 | 0.076 | 2.3E-16 | 62.6 |
| rs887595 | 14 | 74666641 | A | -1.116 | 0.089 | 7.9E-36 | 154.8 |
| rs973879 | 14 | 75297727 | T | -0.692 | 0.069 | 3.5E-25 | 100.5 |
| rs11638121 | 15 | 31425002 | C | 0.546 | 0.089 | 1.2E-11 | 37.1 |
| rs1531638 | 15 | 59924275 | G | -0.466 | 0.072 | 2.2E-11 | 41.0 |
| rs16956273 | 15 | 72117102 | G | -0.487 | 0.080 | 1.2E-08 | 36.3 |
| rs1800407 | 15 | 28230318 | C | 1.136 | 0.123 | 3.7E-21 | 85.0 |
| rs4402521 | 15 | 81879174 | G | 0.495 | 0.085 | 3.6E-10 | 33.4 |
| rs4775761 | 15 | 48723509 | T | -0.656 | 0.103 | 2.5E-10 | 40.6 |
| rs72763822 | 15 | 89143949 | C | -0.658 | 0.072 | 9.2E-20 | 82.7 |
| rs77450879 | 15 | 70749486 | T | 0.697 | 0.127 | 5.0E-08 | 29.9 |
| rs12708665 | 16 | 24728227 | A | -0.422 | 0.076 | 2.8E-09 | 30.7 |
| rs142963458 | 16 | 84561361 | C | -1.376 | 0.192 | 1.5E-13 | 51.1 |
| rs4635359 | 16 | 80537760 | A | -0.699 | 0.078 | 2.0E-22 | 80.0 |
| rs12449442 | 17 | 65947640 | G | -0.577 | 0.086 | 7.2E-12 | 44.5 |
| rs143619610 | 17 | 79356566 | C | -0.562196 | 0.100203 | 4.8E-09 | 31.5 |
| rs2732631 | 17 | 44289232 | G | -0.959832 | 0.0822333 | 3.0E-35 | 136.2 |
| rs36057734 | 17 | 68394967 | T | -0.44937 | 0.0777358 | 2.0E-08 | 33.4 |
| rs7222046 | 17 | 7806529 | A | -0.411201 | 0.0694674 | 2.5E-09 | 35.0 |
| rs723835 | 17 | 59282508 | T | 0.619592 | 0.0833386 | 5.1E-16 | 55.3 |
| rs7405453 | 17 | 79615572 | A | -1.57282 | 0.0714864 | 1.2E-118 | 484.1 |
| rs11662962 | 18 | 57052843 | G | -0.783019 | 0.102856 | 6.0E-15 | 58.0 |
| rs7237204 | 18 | 6730719 | A | 0.510112 | 0.0694295 | 2.0E-14 | 54.0 |
| rs8087238 | 18 | 56943097 | T | -0.756586 | 0.0766748 | 7.0E-24 | 97.4 |
| rs4803297 | 19 | 40310084 | C | 0.556091 | 0.0694798 | 5.3E-17 | 64.1 |
| rs76076446 | 19 | 3771586 | G | 1.29611 | 0.235314 | 2.7E-09 | 30.3 |
| rs113871531 | 20 | 61178527 | C | 0.376993 | 0.0719394 | 1.5E-08 | 27.5 |
| rs9976042 | 21 | 47328990 | T | -0.448075 | 0.0823871 | 8.7E-09 | 29.6 |
| rs75159625 | 22 | 46377008 | T | -0.646923 | 0.0744694 | 7.7E-20 | 75.5 |
| rs9606708 | 22 | 30646126 | G | -0.481125 | 0.0762329 | 7.8E-11 | 39.8 |
| rs9614123 | 22 | 30371350 | A | 0.625081 | 0.0793847 | 5.8E-17 | 62.0 |

**Abbreviations:** CHR, chromosome; SE, standard error.

## **Table S4. Genetic instruments for macular retinal nerve fibre layer thickness**

*V*ariants were extracted from *Currant et al* [13]

| **SNP** | **CHR** | **Position** | **Effect Allele** | **Beta** | **SE** | ***P*-value** | **F-statistic** |
| --- | --- | --- | --- | --- | --- | --- | --- |
| rs13010692 | 2 | 48800667 | C | 0.225 | 0.038 | 5.8E-09 | 33.9 |
| rs2271758 | 2 | 172701157 | G | -0.222 | 0.036 | 1.5E-09 | 36.5 |
| rs980772 | 2 | 145442190 | T | -0.211 | 0.038 | 3.2E-08 | 30.5 |
| rs149831820 | 3 | 77192591 | C | -0.415 | 0.074 | 2.6E-08 | 31.0 |
| rs62252355 | 3 | 69572006 | C | -0.372 | 0.045 | 1.7E-16 | 68.0 |
| rs9818982 | 3 | 45812514 | A | -0.318 | 0.055 | 1.1E-08 | 32.5 |
| rs2004187 | 5 | 2612747 | C | 0.247 | 0.036 | 1.9E-11 | 45.0 |
| rs9486916 | 6 | 109013930 | T | -0.274 | 0.046 | 3.9E-09 | 34.7 |
| rs73348111 | 7 | 50364291 | C | 1.126 | 0.182 | 6.8E-10 | 38.1 |
| rs13271359 | 8 | 109114426 | T | -0.439 | 0.041 | 2.5E-26 | 112.9 |
| rs6989495 | 8 | 74230223 | T | 0.233 | 0.038 | 1.2E-09 | 36.9 |
| rs118031671 | 9 | 10521068 | G | 0.931 | 0.156 | 2.7E-09 | 35.4 |
| rs10762201 | 10 | 70040111 | G | 0.458 | 0.042 | 6.8E-27 | 115.5 |
| rs1947075 | 10 | 49741135 | T | -0.210 | 0.037 | 2.8E-08 | 30.8 |
| rs1042602 | 11 | 88911696 | A | -0.360 | 0.037 | 5.6E-22 | 93.0 |
| rs12574166 | 11 | 69291285 | T | 0.280 | 0.050 | 3.0E-08 | 30.7 |
| rs10140252 | 14 | 74528023 | T | 0.526 | 0.050 | 1.6E-25 | 109.1 |
| rs1254276 | 14 | 60847001 | T | -0.277 | 0.036 | 6.0E-14 | 56.4 |
| rs146652416 | 14 | 29907103 | G | 0.622 | 0.113 | 4.5E-08 | 29.9 |
| rs17095953 | 14 | 59719393 | A | -0.278 | 0.043 | 1.4E-10 | 41.1 |
| rs1470108 | 15 | 89153744 | A | 0.235 | 0.038 | 7.9E-10 | 37.8 |
| rs117304899 | 16 | 15055042 | G | 0.917 | 0.152 | 1.8E-09 | 36.1 |
| rs117300236 | 17 | 44753350 | G | -0.251 | 0.043 | 7.2E-09 | 33.5 |
| rs62075723 | 17 | 79611326 | A | 0.368 | 0.038 | 6.5E-22 | 92.7 |

**Abbreviations:** CHR, chromosome; SE, standard error.

## **Table S5. Genetic instruments for macular ganglion cell inner plexiform layer thickness**

Variants were extracted from *Currant et al* [13]

| **SNP** | **CHR** | **Position** | **Effect Allele** | **Beta** | **SE** | ***P*-value** | **F statistic** |
| --- | --- | --- | --- | --- | --- | --- | --- |
| rs72739513 | 1 | 203080149 | A | 0.716 | 0.125 | 1.1E-08 | 32.7 |
| rs12998032 | 2 | 159095496 | C | 0.300 | 0.048 | 3.9E-10 | 39.2 |
| rs13083522 | 3 | 3270368 | G | 0.319 | 0.057 | 2.5E-08 | 31.0 |
| rs17279437 | 3 | 45814094 | A | -0.776 | 0.076 | 5.5E-24 | 102.2 |
| rs66511946 | 4 | 184932935 | G | -0.367 | 0.049 | 1.0E-13 | 55.4 |
| rs17318932 | 5 | 87688421 | T | -0.535 | 0.096 | 2.6E-08 | 31.0 |
| rs17421627 | 5 | 87847586 | G | 1.024 | 0.090 | 1.6E-29 | 127.5 |
| rs2004187 | 5 | 2612747 | C | 0.276 | 0.048 | 1.1E-08 | 32.6 |
| rs13215351 | 6 | 84313801 | T | -0.340 | 0.054 | 5.7E-10 | 38.4 |
| rs9398171 | 6 | 108983527 | T | 0.502 | 0.052 | 8.3E-22 | 92.2 |
| rs4446688 | 7 | 50993477 | T | 0.334 | 0.054 | 7.9E-10 | 37.8 |
| rs4871827 | 8 | 121061879 | A | -0.293 | 0.050 | 6.6E-09 | 33.7 |
| rs2787394 | 9 | 103007414 | T | -0.284 | 0.048 | 4.3E-09 | 34.5 |
| rs12285584 | 11 | 88935088 | T | -0.315 | 0.049 | 2.1E-10 | 40.4 |
| rs2008905 | 11 | 17184623 | T | -0.361 | 0.048 | 6.5E-14 | 56.2 |
| rs35337422 | 14 | 104407243 | C | 0.404 | 0.067 | 1.8E-09 | 36.1 |
| rs62005075 | 14 | 74476024 | T | 0.526 | 0.075 | 2.6E-12 | 49.0 |
| rs1800407 | 15 | 28230318 | T | -0.594 | 0.086 | 6.5E-12 | 47.2 |
| rs7503894 | 17 | 79583473 | C | 0.553 | 0.050 | 3.2E-28 | 121.6 |
| rs4803297 | 19 | 40310084 | T | -0.265 | 0.048 | 3.2E-08 | 30.5 |
| rs7277632 | 21 | 47327542 | G | 0.348 | 0.053 | 5.6E-11 | 43.0 |

**Abbreviations:** CHR, chromosome; SE, standard error.

## **Table S6. Genetic instruments for retinal outer nuclear layer thickness**

Variants were extracted from *Current et al.* [14]

| **SNP** | **CHR** | **Position** | **Effect Allele** | **Beta** | **SE** | ***P*-value** | **F-statistic** |
| --- | --- | --- | --- | --- | --- | --- | --- |
| rs11118920 | 1 | 222155060 | T | 0.297 | 0.052 | 1.5E-08 | 32.0 |
| rs2128416 | 1 | 10700448 | C | -0.519 | 0.073 | 1.9E-12 | 49.6 |
| rs6426584 | 1 | 227374949 | T | 0.301 | 0.053 | 1.6E-08 | 31.9 |
| rs6427827 | 1 | 200398387 | A | 0.417 | 0.051 | 7.2E-16 | 65.1 |
| rs919655 | 1 | 214157972 | A | 0.471 | 0.077 | 1.0E-09 | 37.2 |
| rs116350483 | 2 | 145338686 | T | -2.125 | 0.198 | 1.0E-26 | 114.6 |
| rs72959706 | 2 | 218533177 | G | -0.598 | 0.099 | 1.8E-09 | 36.2 |
| rs11129176 | 3 | 25049310 | A | 0.314 | 0.055 | 1.6E-08 | 31.9 |
| rs4681617 | 3 | 150122569 | C | 0.467 | 0.083 | 1.9E-08 | 31.6 |
| rs6775323 | 3 | 27721085 | T | 0.493 | 0.062 | 3.4E-15 | 62.1 |
| rs1109114 | 5 | 148615946 | T | 0.382 | 0.050 | 6.1E-14 | 56.4 |
| rs17421627 | 5 | 87847586 | G | 1.150 | 0.095 | 3.6E-33 | 144.3 |
| rs30373 | 5 | 55745334 | G | 0.320 | 0.052 | 9.6E-10 | 37.4 |
| rs62391700 | 5 | 126093395 | C | -0.425 | 0.062 | 1.0E-11 | 46.2 |
| rs63338061 | 5 | 71486228 | T | 0.327 | 0.052 | 4.6E-10 | 38.8 |
| rs6875105 | 5 | 173054917 | C | -0.366 | 0.051 | 1.5E-12 | 50.1 |
| rs12192672 | 6 | 7229619 | A | -0.334 | 0.054 | 1.2E-09 | 36.9 |
| rs4711420 | 6 | 35495811 | T | 0.359 | 0.062 | 1.0E-08 | 32.7 |
| rs6910414 | 6 | 56726737 | G | 0.374 | 0.065 | 1.1E-08 | 32.6 |
| rs74526772 | 6 | 106515218 | A | -1.245 | 0.137 | 1.3E-19 | 82.1 |
| rs12719025 | 7 | 51100190 | G | 0.490 | 0.051 | 9.3E-22 | 92.0 |
| rs34926272 | 7 | 129591807 | C | -1.070 | 0.157 | 9.7E-12 | 46.4 |
| rs929511 | 7 | 8003017 | T | 0.459 | 0.077 | 2.9E-09 | 35.2 |
| rs9639276 | 7 | 867033 | T | 0.427 | 0.066 | 1.1E-10 | 41.6 |
| rs13263941 | 8 | 109121945 | C | 0.854 | 0.057 | 6.1E-50 | 221.5 |
| rs61675430 | 8 | 61671071 | A | -0.405 | 0.063 | 1.4E-10 | 41.2 |
| rs10781177 | 9 | 76593011 | T | -0.293 | 0.051 | 1.0E-08 | 32.8 |
| rs717299 | 9 | 77185933 | G | 0.359 | 0.050 | 1.2E-12 | 50.4 |
| rs9298817 | 9 | 21576591 | C | -0.488 | 0.052 | 2.6E-20 | 85.3 |
| rs1947075 | 10 | 49741135 | T | -0.326 | 0.052 | 5.9E-10 | 38.4 |
| rs7916697 | 10 | 69991853 | G | -0.427 | 0.058 | 3.3E-13 | 53.0 |
| rs1016934 | 11 | 31720621 | G | -0.371 | 0.055 | 1.6E-11 | 45.4 |
| rs116233906 | 11 | 68968271 | A | -0.926 | 0.126 | 2.7E-13 | 53.4 |
| rs1485995 | 11 | 69307707 | A | -0.326 | 0.053 | 1.3E-09 | 36.8 |
| rs6483429 | 11 | 95239787 | T | -0.296 | 0.050 | 4.5E-09 | 34.4 |
| rs3138142 | 12 | 56115585 | T | 0.929 | 0.058 | 1.6E-56 | 251.9 |
| rs76629482 | 12 | 96178789 | G | -0.528 | 0.065 | 6.8E-16 | 65.2 |
| rs2793783 | 13 | 100253216 | G | -0.288 | 0.052 | 3.8E-08 | 30.2 |
| rs112109204 | 14 | 74101756 | T | 0.977 | 0.125 | 7.1E-15 | 60.6 |
| rs118186707 | 14 | 74686575 | A | 1.778 | 0.149 | 9.7E-33 | 142.3 |
| rs28468687 | 14 | 36026342 | A | 0.378 | 0.066 | 1.3E-08 | 32.3 |
| rs7157918 | 14 | 69641807 | A | 0.334 | 0.057 | 6.0E-09 | 33.8 |
| rs887595 | 14 | 74666641 | G | 1.922 | 0.064 | 8.9E-191 | 880.1 |
| rs888413 | 14 | 75267396 | T | 0.392 | 0.050 | 7.1E-15 | 60.6 |
| rs10083695 | 15 | 53987147 | G | -0.367 | 0.050 | 3.4E-13 | 53.0 |
| rs1372613 | 15 | 101204835 | T | 0.357 | 0.054 | 6.9E-11 | 42.6 |
| rs1648303 | 15 | 45445692 | G | -0.278 | 0.050 | 4.0E-08 | 30.1 |
| rs4782636 | 16 | 84585176 | T | 0.406 | 0.064 | 2.5E-10 | 40.0 |
| rs7206532 | 16 | 80490131 | C | 0.293 | 0.050 | 5.1E-09 | 34.1 |
| rs111825734 | 17 | 44019107 | G | 0.484 | 0.059 | 6.2E-16 | 65.4 |
| rs8077430 | 17 | 79511135 | G | -0.488 | 0.059 | 2.7E-16 | 67.0 |
| rs12458015 | 18 | 53305735 | C | -0.294 | 0.053 | 3.0E-08 | 30.7 |
| rs17696543 | 18 | 56971398 | T | -0.560 | 0.065 | 1.7E-17 | 72.5 |
| rs76076446 | 19 | 3771586 | A | -1.231 | 0.168 | 2.6E-13 | 53.5 |
| rs8132685 | 21 | 34220618 | T | -0.296 | 0.050 | 4.2E-09 | 34.5 |
| rs2032576 | 22 | 27089655 | T | -0.277 | 0.050 | 4.4E-08 | 30.0 |
| rs2073946 | 22 | 30619599 | A | 0.469 | 0.055 | 2.0E-17 | 72.2 |
| rs75159625 | 22 | 46377008 | G | 0.339 | 0.054 | 3.9E-10 | 39.2 |

**Abbreviations:** CHR, chromosome; SE, standard error.

## **Table S7. Genetic instruments for retinal inner segment thickness**

Variants were extracted from *Current et al.* [14]

| **SNP** | **CHR** | **Position** | **Effect Allele** | **Beta** | **SE** | ***P*-value** | **F-statistic** |
| --- | --- | --- | --- | --- | --- | --- | --- |
| rs112248193 | 1 | 3718402 | A | -0.105 | 0.017 | 1.3E-09 | 36.7 |
| rs6665290 | 1 | 227201106 | T | 0.070 | 0.011 | 3.2E-09 | 35.0 |
| rs1463229 | 3 | 150167912 | G | -0.091 | 0.013 | 2.8E-11 | 44.3 |
| rs2351248 | 3 | 47829277 | T | -0.071 | 0.012 | 2.7E-08 | 30.9 |
| rs72761109 | 5 | 71506529 | T | 0.071 | 0.012 | 2.3E-08 | 31.2 |
| rs3779791 | 8 | 87676710 | C | 0.095 | 0.013 | 4.4E-12 | 47.9 |
| rs7816990 | 8 | 10463944 | C | -0.078 | 0.013 | 7.1E-09 | 33.5 |
| rs2808051 | 10 | 32205630 | C | 0.070 | 0.011 | 2.4E-09 | 35.6 |
| rs35314358 | 10 | 62647787 | A | 0.085 | 0.014 | 2.5E-09 | 35.5 |
| rs2292962 | 11 | 1778321 | T | 0.098 | 0.017 | 2.7E-08 | 30.9 |
| rs618838 | 11 | 66328719 | C | 0.070 | 0.011 | 2.2E-09 | 35.7 |
| rs1272131 | 14 | 60886150 | C | -0.089 | 0.012 | 1.0E-13 | 55.4 |
| rs887595 | 14 | 74666641 | G | 0.182 | 0.015 | 8.3E-33 | 142.6 |
| rs10220961 | 16 | 71789550 | G | 0.081 | 0.011 | 4.2E-12 | 48.0 |
| rs35638197 | 17 | 47284735 | C | -0.139 | 0.024 | 2.1E-08 | 31.4 |

**Abbreviations:** CHR, chromosome; SE, standard error.

## **Table S8. Genetic instruments for retinal outer segment thickness**

Variants were extracted from *Current et al*. [14]

| **SNP** | **CHR** | **Position** | **Effect Allele** | **Beta** | **SE** | ***P*-value** | **F-statistic** |
| --- | --- | --- | --- | --- | --- | --- | --- |
| rs6668533 | 1 | 113064468 | A | -0.275 | 0.034 | 1.8E-15 | 63.3 |
| rs6669802 | 1 | 110163076 | G | 0.168 | 0.030 | 4.1E-08 | 30.1 |
| rs72683442 | 1 | 113468825 | C | -0.341 | 0.036 | 9.7E-21 | 87.3 |
| rs1866665 | 2 | 198925980 | G | 0.230 | 0.040 | 1.2E-08 | 32.4 |
| rs6737602 | 2 | 169062731 | C | 0.194 | 0.031 | 5.1E-10 | 38.6 |
| rs7564805 | 2 | 234228946 | G | -0.789 | 0.069 | 1.4E-29 | 127.7 |
| rs7594221 | 2 | 24112724 | A | 0.271 | 0.035 | 3.3E-14 | 57.6 |
| rs34234056 | 3 | 14418444 | G | 0.173 | 0.030 | 1.5E-08 | 32.0 |
| rs62282867 | 3 | 100972078 | A | 0.351 | 0.037 | 9.4E-21 | 87.4 |
| rs7430585 | 3 | 150112041 | A | 0.221 | 0.035 | 4.4E-10 | 38.9 |
| rs115237855 | 5 | 17186336 | A | -0.692 | 0.096 | 8.5E-13 | 51.2 |
| rs2326838 | 6 | 6901663 | A | -0.227 | 0.031 | 7.2E-13 | 51.5 |
| rs375435 | 6 | 42661404 | C | -0.253 | 0.030 | 2.3E-16 | 67.3 |
| rs9470055 | 6 | 35521961 | A | 0.222 | 0.039 | 1.9E-08 | 31.5 |
| rs947340 | 6 | 76747944 | C | -0.248 | 0.033 | 1.5E-13 | 54.6 |
| rs111963714 | 7 | 99948655 | G | 0.265 | 0.037 | 1.27E-12 | 50.4 |
| rs62490856 | 8 | 10469030 | A | 0.349 | 0.046 | 3.5E-14 | 57.5 |
| rs11200922 | 10 | 85961758 | G | 0.322 | 0.030 | 2.6E-26 | 112.8 |
| rs60401382 | 10 | 124227624 | T | -0.304 | 0.036 | 5.3E-17 | 70.3 |
| rs1126809 | 11 | 89017961 | A | -0.335 | 0.033 | 3.3E-24 | 103.2 |
| rs12574286 | 11 | 76937602 | C | -0.296 | 0.037 | 2.0E-15 | 63.1 |
| rs2080402 | 12 | 345175 | C | 0.224 | 0.030 | 2.7E-13 | 53.4 |
| rs3138141 | 12 | 56115778 | A | 0.428 | 0.034 | 4.0E-35 | 153.3 |
| rs9796234 | 13 | 114323997 | C | 0.260 | 0.030 | 1.1E-17 | 73.3 |
| rs1254260 | 14 | 60835737 | A | -0.226 | 0.033 | 1.5E-11 | 45.5 |
| rs1800407 | 15 | 28230318 | T | 0.580 | 0.055 | 9.3E-26 | 110.3 |
| rs183626410 | 15 | 55738274 | T | -0.727 | 0.128 | 1.5E-08 | 32.0 |
| rs3825991 | 15 | 89761664 | A | 0.357 | 0.030 | 3.5E-31 | 135.2 |
| rs142963458 | 16 | 84561361 | T | -0.778 | 0.084 | 2.1E-20 | 85.7 |
| rs4794029 | 17 | 47280301 | C | 0.237 | 0.032 | 4.2E-13 | 52.6 |
| rs7405453 | 17 | 79615572 | G | -0.410 | 0.032 | 1.8E-37 | 164.0 |
| rs1232603 | 20 | 10612963 | T | -0.228 | 0.032 | 2.2E-12 | 49.3 |
| rs6077977 | 20 | 10930708 | G | 0.279 | 0.030 | 3.0E-20 | 85.1 |

**Abbreviations:** CHR, chromosome; SE, standard error.

## **Table S9. Genetic instruments for retinal arteriolar tortuosity**

Variants extracted from *Jiang et al* [15]

| **SNP** | **CHR** | **Position** | **Effect Allele** | **Beta** | **SE** | ***P*-value** | **F-statistic** |
| --- | --- | --- | --- | --- | --- | --- | --- |
| rs12116477 | 1 | 110636625 | G | -0.035 | 0.003 | 5.8E-29 | 133.3 |
| rs12118331 | 1 | 68314967 | C | -0.020 | 0.003 | 1.3E-09 | 37.6 |
| rs12569180 | 1 | 92332419 | G | -0.029 | 0.003 | 4.4E-19 | 85.1 |
| rs16846877 | 1 | 227236070 | G | -0.027 | 0.004 | 6.1E-11 | 44.7 |
| rs2147324 | 1 | 150255587 | T | -0.042 | 0.002 | 9.8E-45 | 215.6 |
| rs2500257 | 1 | 3264297 | A | 0.020 | 0.003 | 1.8E-09 | 41.0 |
| rs2820299 | 1 | 201907874 | A | 0.038 | 0.005 | 1.0E-11 | 52.8 |
| rs3007364 | 1 | 169018902 | G | -0.018 | 0.003 | 1.2E-09 | 37.1 |
| rs4656767 | 1 | 170379727 | A | 0.020 | 0.003 | 1.7E-10 | 39.1 |
| rs533713 | 1 | 91900353 | A | -0.021 | 0.003 | 9.9E-10 | 39.7 |
| rs9426677 | 1 | 22249836 | C | -0.019 | 0.003 | 3.9E-08 | 33.9 |
| rs10170172 | 2 | 134303364 | G | 0.023 | 0.003 | 4.4E-14 | 63.3 |
| rs12694223 | 2 | 212031854 | T | 0.023 | 0.003 | 1.8E-13 | 61.0 |
| rs2571461 | 2 | 218670945 | T | 0.051 | 0.003 | 6.4E-65 | 294.8 |
| rs28446330 | 2 | 242392759 | T | -0.039 | 0.004 | 2.1E-17 | 66.7 |
| rs6720394 | 2 | 111989372 | T | 0.024 | 0.004 | 4.4E-09 | 27.1 |
| rs68106030 | 2 | 11495670 | C | 0.056 | 0.006 | 2.6E-15 | 70.3 |
| rs2130320 | 3 | 149548794 | G | -0.021 | 0.003 | 5.2E-11 | 47.6 |
| rs59213344 | 3 | 20171631 | G | -0.040 | 0.006 | 2.4E-10 | 38.2 |
| rs113409736 | 4 | 120195841 | G | 0.026 | 0.003 | 1.6E-14 | 67.1 |
| rs4450996 | 4 | 1795321 | C | 0.019 | 0.003 | 3.2E-08 | 35.5 |
| rs62320736 | 4 | 120278368 | C | -0.059 | 0.003 | 2.2E-46 | 232.3 |
| rs6833227 | 4 | 8270320 | G | 0.032 | 0.003 | 4.5E-23 | 98.9 |
| rs9992012 | 4 | 85029012 | G | 0.050 | 0.002 | 4.3E-59 | 289.4 |
| rs10060136 | 5 | 77907628 | A | 0.036 | 0.002 | 9.9E-33 | 157.6 |
| rs1019992 | 5 | 89523523 | T | -0.027 | 0.004 | 2.5E-09 | 36.7 |
| rs10805388 | 5 | 63888835 | A | -0.019 | 0.003 | 2.6E-08 | 36.8 |
| rs2885809 | 5 | 78018768 | G | -0.022 | 0.003 | 1.0E-12 | 52.5 |
| rs41287110 | 5 | 149509446 | C | 0.060 | 0.009 | 8.5E-11 | 42.3 |
| rs6864465 | 5 | 1110797 | T | 0.020 | 0.003 | 1.3E-10 | 45.9 |
| rs6867399 | 5 | 52135543 | C | -0.018 | 0.003 | 2.9E-08 | 30.9 |
| rs9112 | 5 | 121518309 | G | 0.018 | 0.003 | 1.2E-09 | 39.2 |
| rs1055366 | 6 | 907745 | C | -0.021 | 0.003 | 3.2E-10 | 42.4 |
| rs17080102 | 6 | 151004770 | G | 0.049 | 0.005 | 3.8E-16 | 73.6 |
| rs2504803 | 6 | 39822183 | T | -0.026 | 0.004 | 4.4E-08 | 36.5 |
| rs36017416 | 6 | 39871532 | A | -0.053 | 0.006 | 1.0E-15 | 71.3 |
| rs9296392 | 6 | 12476696 | A | 0.036 | 0.004 | 2.0E-17 | 87.3 |
| rs9328455 | 6 | 864095 | C | -0.029 | 0.003 | 1.8E-16 | 80.7 |
| rs9405472 | 6 | 1385814 | C | -0.033 | 0.003 | 2.8E-22 | 114.1 |
| rs11771811 | 7 | 45200374 | A | 0.023 | 0.003 | 3.4E-09 | 43.8 |
| rs10101740 | 8 | 38556927 | T | 0.021 | 0.003 | 2.9E-09 | 35.9 |
| rs10958733 | 8 | 38424123 | T | 0.048 | 0.004 | 1.9E-22 | 100.0 |
| rs118139256 | 8 | 38411245 | G | -0.082 | 0.010 | 4.7E-14 | 60.9 |
| rs396498 | 8 | 109156783 | C | -0.023 | 0.003 | 7.4E-10 | 46.5 |
| rs9657494 | 8 | 18941575 | G | -0.017 | 0.003 | 3.8E-09 | 33.7 |
| rs10820860 | 9 | 94253603 | T | 0.026 | 0.003 | 9.9E-14 | 69.1 |
| rs115478735 | 9 | 136149711 | A | -0.029 | 0.004 | 1.6E-13 | 58.8 |
| rs913990 | 9 | 130538720 | T | -0.021 | 0.003 | 1.1E-10 | 46.3 |
| rs10733793 | 10 | 65323809 | T | -0.016 | 0.003 | 2.9E-08 | 32.7 |
| rs11191822 | 10 | 105599770 | T | 0.018 | 0.003 | 2.9E-10 | 38.4 |
| rs181676 | 10 | 119391213 | G | -0.023 | 0.003 | 1.5E-13 | 54.5 |
| rs2446573 | 10 | 13999224 | G | -0.017 | 0.003 | 4.8E-08 | 30.9 |
| rs11042104 | 11 | 8890924 | G | 0.021 | 0.003 | 2.4E-09 | 41.8 |
| rs3781660 | 11 | 70002689 | C | -0.036 | 0.003 | 3.6E-26 | 122.0 |
| rs11045245 | 12 | 20589390 | A | -0.048 | 0.003 | 1.2E-54 | 259.1 |
| rs12827748 | 12 | 80088578 | T | 0.032 | 0.003 | 4.2E-22 | 104.1 |
| rs1747223 | 13 | 47224681 | A | -0.030 | 0.003 | 5.7E-18 | 80.0 |
| rs61963281 | 13 | 110883774 | C | 0.031 | 0.005 | 5.2E-10 | 41.8 |
| rs7319323 | 13 | 110955187 | T | -0.028 | 0.003 | 5.3E-18 | 79.8 |
| rs9551466 | 13 | 28967817 | G | 0.034 | 0.003 | 5.9E-26 | 117.4 |
| rs10483727 | 14 | 61072875 | T | 0.018 | 0.003 | 6.9E-10 | 36.6 |
| rs3748344 | 14 | 21494477 | C | 0.016 | 0.003 | 4.2E-08 | 28.9 |
| rs56130943 | 14 | 105906522 | A | -0.049 | 0.003 | 1.2E-41 | 197.1 |
| rs7140617 | 14 | 76109364 | C | 0.021 | 0.003 | 1.7E-08 | 40.7 |
| rs12913832 | 15 | 28365618 | A | -0.020 | 0.003 | 4.1E-09 | 33.3 |
| rs2009972 | 15 | 52106301 | G | -0.028 | 0.002 | 2.1E-20 | 88.7 |
| rs4886410 | 15 | 75065644 | G | 0.046 | 0.003 | 2.5E-45 | 213.9 |
| rs62046379 | 15 | 56299233 | G | 0.043 | 0.005 | 1.1E-12 | 53.1 |
| rs12918234 | 16 | 75508453 | C | 0.025 | 0.003 | 2.3E-12 | 44.7 |
| rs5020073 | 16 | 86386971 | A | 0.025 | 0.004 | 2.2E-08 | 37.1 |
| rs6498489 | 16 | 14087829 | C | -0.021 | 0.003 | 4.2E-09 | 34.7 |
| rs11655719 | 17 | 56518534 | T | -0.031 | 0.004 | 5.9E-13 | 47.2 |
| rs16975575 | 17 | 68323746 | T | 0.047 | 0.003 | 9.0E-37 | 186.0 |
| rs4790870 | 17 | 1970201 | C | -0.027 | 0.003 | 1.7E-17 | 84.4 |
| rs11662380 | 18 | 20638059 | C | 0.019 | 0.003 | 2.5E-08 | 27.5 |
| rs10415219 | 19 | 39150235 | A | 0.044 | 0.003 | 2.1E-49 | 226.9 |
| rs10415769 | 19 | 46158643 | G | -0.019 | 0.003 | 3.2E-09 | 34.3 |
| rs76301301 | 19 | 14589068 | T | -0.022 | 0.003 | 8.6E-09 | 35.0 |
| rs143373163 | 20 | 33752110 | G | 0.032 | 0.005 | 9.9E-11 | 38.8 |
| rs2267406 | 22 | 39633749 | T | -0.034 | 0.003 | 8.2E-22 | 103.3 |
| rs2587106 | 22 | 18320311 | A | -0.018 | 0.003 | 4.3E-08 | 34.1 |
| rs5750494 | 22 | 38176979 | T | -0.016 | 0.003 | 2.0E-08 | 30.8 |

**Abbreviations:** CHR, chromosome; SE, standard error.

## **Table S10: Genetic instruments for retinal venular tortuosity**

Variants extracted from *Jiang et al* [15]

| **SNP** | **CHR** | **Position** | **Effect Allele** | **Beta** | **SE** | ***P*-value** | **F-statistic** |
| --- | --- | --- | --- | --- | --- | --- | --- |
| rs11122106 | 1 | 6681262 | A | 0.013 | 0.002 | 8.4E-12 | 49.2 |
| rs28446330 | 2 | 242392759 | T | -0.020 | 0.003 | 2.9E-12 | 45.2 |
| rs6430425 | 2 | 134277480 | C | 0.013 | 0.002 | 2.8E-12 | 49.8 |
| rs918949 | 2 | 218674697 | C | 0.018 | 0.002 | 2.2E-23 | 102.0 |
| rs115929714 | 3 | 8497234 | G | -0.030 | 0.004 | 5.1E-13 | 54.8 |
| rs7669726 | 4 | 146891096 | T | -0.011 | 0.002 | 2.5E-09 | 36.9 |
| rs168189 | 5 | 77974427 | C | -0.018 | 0.002 | 4.7E-19 | 82.5 |
| rs9641196 | 7 | 96462308 | G | -0.022 | 0.002 | 8.4E-32 | 143.1 |
| rs10760220 | 9 | 125026162 | A | -0.012 | 0.002 | 5.3E-11 | 44.4 |
| rs10982622 | 9 | 100304531 | A | -0.010 | 0.002 | 1.0E-08 | 31.6 |
| rs2249006 | 9 | 130330607 | G | 0.016 | 0.002 | 4.4E-15 | 62.3 |
| rs12910951 | 15 | 77806797 | A | -0.014 | 0.002 | 1.1E-13 | 54.6 |
| rs8053277 | 16 | 51469726 | T | -0.013 | 0.002 | 1.4E-10 | 43.3 |
| rs12603193 | 17 | 70387448 | A | -0.010 | 0.002 | 6.6E-09 | 29.9 |
| rs35825162 | 17 | 75373188 | G | -0.011 | 0.002 | 6.4E-10 | 39.1 |
| rs10415219 | 19 | 39150235 | A | 0.044 | 0.002 | 4.4E-128 | 584.0 |

**Abbreviations:** CHR, chromosome; SE, standard error.

## **Table S11. Genetic instruments for retinal venular width**

Variants extracted from *Jiang et al* [15]

| **SNP** | **CHR** | **Position** | **Effect Allele** | **Beta** | **SE** | ***P*-value** | **F-statistic** |
| --- | --- | --- | --- | --- | --- | --- | --- |
| rs10737680 | 1 | 196679455 | A | 0.488 | 0.077 | 1.6E-10 | 40.0 |
| rs115456027 | 5 | 87919700 | C | -1.066 | 0.143 | 2.4E-13 | 55.1 |
| rs225647 | 6 | 142483643 | A | -0.798 | 0.088 | 9.6E-18 | 81.1 |
| rs10089615 | 8 | 10634132 | C | 0.538 | 0.077 | 3.4E-12 | 47.7 |
| rs77391671 | 8 | 130183779 | A | -0.659 | 0.118 | 2.1E-08 | 31.0 |
| rs10733840 | 10 | 69980221 | A | 0.604 | 0.096 | 8.9E-10 | 39.3 |
| rs9513111 | 13 | 28997563 | C | 0.584 | 0.0873 | 2.4E-11 | 44.8 |
| rs2614205 | 15 | 54004516 | C | 0.536 | 0.076 | 2.7E-12 | 49.7 |
| rs4932480 | 15 | 89723858 | T | -0.505 | 0.076 | 9.6E-12 | 43.7 |
| rs2303040 | 19 | 39138608 | T | -0.508 | 0.076 | 2.1E-11 | 44.6 |
| rs4002471 | 19 | 49215095 | C | -0.671 | 0.076 | 9.9E-19 | 76.5 |
| rs7265807 | 20 | 22695089 | C | -0.471 | 0.081 | 7.0E-09 | 33.1 |
| rs2836445 | 21 | 39872696 | G | 0.432 | 0.078 | 2.6E-08 | 30.7 |

**Abbreviations:** CHR, chromosome; SE, standard error.

## **Table S12. Genetic instruments for retinal vessel fractal dimension**

Variants extracted from *Villaplana-Velasco et al [16]*

| **SNP** | **CHR** | **Position** | **Effect Allele** | **Beta** | **SE** | ***P*-value** | **F statistic** |
| --- | --- | --- | --- | --- | --- | --- | --- |
| rs12203592 | 6 | 396321 | C | -0.003 | 0.0002 | 6.2E-39 | 170.7 |
| rs3138141 | 12 | 56115778 | C | -0.001 | 0.0002 | 1.0E-09 | 37.3 |
| rs12913832 | 15 | 28365618 | G | 0.006 | 0.0002 | 6.3E-173 | 793.9 |

CHR, chromosome; SE, standard error.

## **Table S13. Genetic instruments for optic disc area**

Variants extracted from *Springelkamp et al*^1^

| **SNP** | **CHR** | **Position** | **Effect Allele** | **Beta** | **SE** | ***P*-value** | **F statistic** |
| --- | --- | --- | --- | --- | --- | --- | --- |
| rs11811982 | 1 | 227393819 | A | -0.062 | 0.008 | 9.7E-15 | 60.3 |
| rs2239854 | 1 | 169556570 | A | 0.030 | 0.004 | 2.5E-10 | 39.6 |
| rs4658101 | 1 | 91611852 | A | 0.089 | 0.005 | 1.9E-60 | 272.2 |
| rs9843102 | 3 | 100932085 | A | -0.034 | 0.006 | 1.5E-09 | 36.0 |
| rs10021731 | 4 | 114560759 | T | 0.025 | 0.004 | 1.6E-08 | 31.4 |
| rs6468996 | 8 | 87723109 | T | 0.025 | 0.004 | 2.2E-09 | 36.3 |
| rs7916410 | 10 | 68235910 | T | -0.101 | 0.005 | 1.6E-90 | 408.8 |
| rs1223068 | 11 | 31458802 | T | -0.033 | 0.005 | 1.8E-10 | 39.7 |
| rs442376 | 12 | 83584338 | T | -0.034 | 0.004 | 3.3E-13 | 52.9 |
| rs1345467 | 16 | 51448410 | A | -0.034 | 0.005 | 3.7E-13 | 52.6 |
| rs56385951 | 22 | 37513532 | A | 0.046 | 0.007 | 2.3E-10 | 39.7 |

**Abbreviations:** CHR, chromosome; SE, standard error.

## **Table S14. Genetic instruments for optic cup area**

Variants extracted from the GWAS reported by *Springelkamp et al*^1^

| **SNP** | **CHR** | **Position** | **Effect Allele** | **Beta** | **SE** | ***P*-value** | **F-statistic** |
| --- | --- | --- | --- | --- | --- | --- | --- |
| rs140491439 | 1 | 12840151 | A | -0.249 | 0.033 | 6.7E-14 | 56.2 |
| rs146096977 | 1 | 153581642 | A | 0.838 | 0.064 | 3.4E-39 | 171.5 |
| rs41308373 | 1 | 204142779 | A | -0.197 | 0.027 | 3.6E-13 | 52.8 |
| rs73102394 | 1 | 227388451 | T | 0.027 | 0.004 | 3.3E-09 | 34.4 |
| rs151199024 | 2 | 66645530 | T | 0.180 | 0.032 | 3.1E-08 | 30.7 |
| rs188127885 | 2 | 33897567 | T | 0.237 | 0.032 | 2.8E-13 | 53.2 |
| rs112312463 | 3 | 173365932 | T | 0.186 | 0.0291 | 1.5E-10 | 41.0 |
| rs147595840 | 3 | 141034771 | T | 0.217 | 0.027 | 1.6E-15 | 63.2 |
| rs113418905 | 4 | 56173287 | T | -0.212 | 0.036 | 8.2E-09 | 33.2 |
| rs143650238 | 4 | 9575658 | T | -0.282 | 0.037 | 6.8E-14 | 56.2 |
| rs35084382 | 5 | 172770036 | T | 0.033 | 0.006 | 2.2E-08 | 31.5 |
| rs75613839 | 5 | 147392985 | A | -0.353 | 0.051 | 8.8E-12 | 46.5 |
| rs117598310 | 8 | 75086861 | T | 0.030 | 0.005 | 1.3E-08 | 32.1 |
| rs62502385 | 8 | 28309567 | T | 0.254 | 0.032 | 2.6E-15 | 62.7 |
| rs72697677 | 8 | 144681585 | A | 0.322 | 0.035 | 4.2E-20 | 84.1 |
| rs75336139 | 8 | 120438132 | A | 0.690 | 0.060 | 4.1E-30 | 129.9 |
| rs1360589 | 9 | 22045318 | T | 0.025 | 0.002 | 3.1E-27 | 118.1 |
| rs140770901 | 9 | 130186561 | T | 0.195 | 0.031 | 8.8E-10 | 37.6 |
| rs147126801 | 9 | 130042848 | T | 0.164 | 0.022 | 1.7E-13 | 54.4 |
| rs190500685 | 9 | 1477038 | A | 0.291 | 0.035 | 1.4E-16 | 68.5 |
| rs113357323 | 10 | 106281573 | T | -0.179 | 0.031 | 1.0E-08 | 32.8 |
| rs140547220 | 10 | 76209754 | T | 0.232 | 0.032 | 9.9E-13 | 50.8 |
| rs151081473 | 10 | 35828063 | T | 0.201 | 0.028 | 1.8E-12 | 49.6 |
| rs7916697 | 10 | 68232096 | A | -0.021 | 0.002 | 6.3E-15 | 60.1 |
| rs11613189 | 12 | 124158257 | T | -0.015 | 0.002 | 3.7E-10 | 38.4 |
| rs149889322 | 12 | 132550482 | T | 0.466 | 0.077 | 1.7E-09 | 36.3 |
| rs482507 | 12 | 83585507 | T | 0.018 | 0.002 | 6.0E-15 | 61.4 |
| rs138060893 | 13 | 113747895 | A | -0.241 | 0.037 | 1.1E-10 | 41.5 |
| rs7323428 | 13 | 36069464 | T | 0.020 | 0.002 | 3.3E-14 | 57.6 |
| rs4436712 | 14 | 60341284 | T | 0.0226 | 0.0025 | 9.8E-20 | 81.7 |
| rs6598351 | 15 | 98264882 | T | 0.0192 | 0.0034 | 1.7E-08 | 31.9 |
| rs11646917 | 16 | 51394997 | T | -0.016 | 0.0028 | 8.8E-09 | 32.7 |
| rs186736573 | 16 | 69631718 | T | 0.2 | 0.0329 | 1.1E-09 | 37.0 |
| rs11867840 | 17 | 61195904 | A | 0.0204 | 0.0028 | 1.3E-13 | 53.1 |
| rs138454650 | 19 | 4735883 | A | 0.1827 | 0.0289 | 2.7E-10 | 40.0 |
| rs184227198 | 19 | 45374792 | T | 0.6402 | 0.0704 | 9.3E-20 | 82.7 |
| rs6054375 | 20 | 6597982 | T | -0.0212 | 0.0026 | 2.9E-16 | 66.5 |
| rs149475779 | 21 | 28022957 | T | 0.2197 | 0.0379 | 6.4E-09 | 33.6 |
| rs111819122 | 22 | 24424312 | A | 0.3191 | 0.0352 | 1.2E-19 | 82.2 |
| rs141215242 | 22 | 48280331 | A | -0.2225 | 0.0334 | 2.6E-11 | 44.4 |

**Abbreviations:** CHR, chromosome; SE, standard error.

## **Table S15. Genetic instruments for vertical cup:disc ratio**

Variants extracted from *Springelkamp et al.* [18]

| **SNP** | **CHR** | **Position** | **Effect Allele** | **Beta** | **SE** | ***P*-value** | **F-statistic** |
| --- | --- | --- | --- | --- | --- | --- | --- |
| rs10753787 | 1 | 169580537 | T | -0.007 | 0.001 | 2.1E-09 | 36.0 |
| rs1192414 | 1 | 91609577 | A | 0.015 | 0.001 | 3.0E-20 | 89.1 |
| rs6804624 | 3 | 99440303 | T | -0.008 | 0.001 | 1.5E-09 | 38.6 |
| rs114503346 | 5 | 172765347 | T | -0.022 | 0.003 | 5.1E-09 | 34.7 |
| rs72759609 | 5 | 31951945 | T | 0.013 | 0.002 | 4.5E-09 | 35.0 |
| rs12208686 | 6 | 65006043 | T | -0.158 | 0.027 | 1.1E-08 | 32.6 |
| rs4960295 | 6 | 7205563 | A | 0.006 | 0.001 | 1.6E-08 | 33.1 |
| rs10274998 | 7 | 14205752 | T | 0.009 | 0.001 | 2.3E-08 | 32.6 |
| rs2157719 | 9 | 22033367 | T | 0.014 | 0.001 | 6.2E-33 | 140.0 |
| rs7916697 | 10 | 68232096 | A | -0.020 | 0.001 | 8.7E-43 | 190.4 |
| rs4936099 | 11 | 130410830 | A | 0.008 | 0.001 | 2.7E-09 | 35.1 |
| rs324780 | 12 | 83610087 | A | 0.011 | 0.001 | 5.7E-21 | 90.2 |
| rs7323428 | 13 | 36069464 | T | 0.009 | 0.001 | 6.1E-10 | 39.3 |
| rs8015152 | 14 | 60345281 | T | 0.010 | 0.001 | 2.9E-13 | 55.2 |
| rs6107845 | 20 | 6598094 | A | -0.010 | 0.001 | 4.8E-17 | 73.7 |
| rs1003342 | 22 | 30174033 | A | 0.006 | 0.001 | 4.3E-08 | 30.2 |
| rs2092172 | 22 | 37511062 | A | 0.008 | 0.001 | 3.5E-08 | 30.2 |

**Abbreviations:** CHR, chromosome; SE, standard error.

## **Table S16. Genetic instruments for refractive error**

Variants extracted from *Hysi et al. [19]*

| **SNP** | **CHR** | **Position** | **Effect Allele** | **Beta** | **SE** | ***P*-value** | **F-statistic** |
| --- | --- | --- | --- | --- | --- | --- | --- |
| rs11121599 | 1 | 10666735 | A | 0.012 | 0.002 | 1.1E-09 | 37.2 |
| rs4839270 | 1 | 113477320 | A | -0.017 | 0.002 | 2.3E-17 | 71.8 |
| rs11581857 | 1 | 114669174 | T | -0.013 | 0.002 | 1.2E-10 | 41.4 |
| rs12138787 | 1 | 150246070 | T | -0.013 | 0.002 | 7.0E-12 | 47.0 |
| rs4845700 | 1 | 154981708 | T | 0.012 | 0.002 | 2.7E-09 | 35.4 |
| rs17420551 | 1 | 158039425 | A | 0.022 | 0.002 | 2.2E-29 | 126.7 |
| rs6673517 | 1 | 164176470 | A | -0.019 | 0.002 | 5.3E-22 | 93.0 |
| rs10919475 | 1 | 170771306 | A | 0.012 | 0.002 | 1.0E-09 | 37.3 |
| rs505789 | 1 | 179896885 | A | 0.013 | 0.002 | 2.6E-10 | 40.0 |
| rs77980226 | 1 | 198767159 | T | -0.011 | 0.002 | 3.4E-08 | 30.5 |
| rs76200047 | 1 | 200058491 | T | 0.012 | 0.002 | 5.1E-09 | 34.1 |
| rs2808514 | 1 | 200343081 | A | 0.024 | 0.002 | 3.4E-34 | 148.7 |
| rs1858001 | 1 | 207488004 | C | 0.027 | 0.002 | 2.7E-43 | 190.4 |
| rs2745939 | 1 | 208142081 | A | 0.015 | 0.002 | 4.3E-14 | 57.0 |
| rs6674686 | 1 | 218859416 | A | 0.012 | 0.002 | 1.2E-09 | 36.9 |
| rs10746387 | 1 | 219787214 | A | 0.022 | 0.002 | 1.4E-29 | 127.6 |
| rs6680922 | 1 | 42331212 | T | -0.021 | 0.002 | 7.3E-27 | 115.2 |
| rs3001723 | 1 | 44037685 | A | 0.012 | 0.002 | 4.4E-09 | 34.4 |
| rs74658637 | 1 | 44494076 | C | 0.011 | 0.002 | 2.0E-08 | 31.5 |
| rs4927030 | 1 | 54182886 | A | 0.012 | 0.002 | 5.9E-10 | 38.4 |
| rs1563953 | 1 | 61058816 | T | -0.018 | 0.002 | 8.0E-21 | 87.6 |
| rs1779868 | 1 | 61476048 | A | 0.015 | 0.002 | 5.1E-14 | 56.7 |
| rs12568187 | 1 | 91193691 | T | 0.019 | 0.002 | 6.2E-21 | 88.1 |
| rs2710873 | 1 | 978193 | A | 0.012 | 0.002 | 4.0E-08 | 30.2 |
| rs11190752 | 10 | 102677376 | T | 0.013 | 0.002 | 2.4E-11 | 44.6 |
| rs807037 | 10 | 102824349 | C | -0.016 | 0.002 | 6.2E-17 | 69.9 |
| rs72826094 | 10 | 114801488 | A | 0.022 | 0.002 | 1.5E-28 | 122.9 |
| rs2142308 | 10 | 124234037 | C | -0.019 | 0.002 | 1.6E-22 | 95.4 |
| rs1693655 | 10 | 126843661 | T | 0.013 | 0.002 | 8.9E-12 | 46.6 |
| rs3739996 | 10 | 30316872 | T | 0.016 | 0.002 | 2.5E-15 | 62.6 |
| rs10793568 | 10 | 45415209 | A | -0.012 | 0.002 | 4.7E-10 | 38.8 |
| rs3906617 | 10 | 49412661 | T | -0.025 | 0.002 | 9.6E-36 | 155.7 |
| rs2842083 | 10 | 60329230 | A | -0.022 | 0.002 | 6.2E-30 | 129.2 |
| rs7101298 | 10 | 62313176 | A | 0.015 | 0.002 | 6.2E-15 | 60.8 |
| rs9415063 | 10 | 74086652 | T | 0.015 | 0.002 | 1.3E-13 | 54.8 |
| rs7912297 | 10 | 75861664 | A | -0.011 | 0.002 | 2.2E-08 | 31.3 |
| rs10824526 | 10 | 79083871 | T | -0.028 | 0.002 | 1.7E-46 | 204.9 |
| rs4517452 | 10 | 86021024 | T | 0.030 | 0.002 | 8.3E-53 | 233.9 |
| rs3781196 | 10 | 90042154 | T | 0.018 | 0.002 | 1.0E-20 | 87.1 |
| rs7916761 | 10 | 94941927 | T | -0.015 | 0.002 | 1.8E-14 | 58.7 |
| rs1002191 | 11 | 105692052 | C | -0.021 | 0.002 | 1.8E-27 | 117.9 |
| rs228592 | 11 | 108123189 | A | 0.016 | 0.002 | 2.7E-15 | 62.5 |
| rs3819132 | 11 | 117671489 | A | 0.020 | 0.002 | 1.1E-23 | 100.6 |
| rs35310980 | 11 | 119236410 | A | 0.013 | 0.002 | 1.1E-09 | 37.1 |
| rs1029278 | 11 | 123421402 | A | -0.016 | 0.002 | 2.4E-16 | 67.3 |
| rs654169 | 11 | 128691920 | A | -0.012 | 0.002 | 1.5E-09 | 36.5 |
| rs7122003 | 11 | 128825500 | T | -0.015 | 0.002 | 5.6E-14 | 56.5 |
| rs949146 | 11 | 131927703 | T | -0.013 | 0.002 | 2.1E-11 | 44.9 |
| rs476637 | 11 | 132991583 | A | -0.011 | 0.002 | 2.5E-08 | 31.0 |
| rs4752755 | 11 | 1756848 | T | -0.013 | 0.002 | 8.2E-11 | 42.2 |
| rs7950091 | 11 | 18758431 | A | -0.016 | 0.002 | 1.5E-15 | 63.6 |
| rs6484377 | 11 | 28653488 | T | 0.014 | 0.002 | 4.6E-13 | 52.3 |
| rs524373 | 11 | 30027074 | A | -0.025 | 0.002 | 9.6E-38 | 164.9 |
| rs67745574 | 11 | 30234839 | T | 0.017 | 0.002 | 2.8E-17 | 71.5 |
| rs11606250 | 11 | 40149300 | A | -0.043 | 0.002 | 1.5E-102 | 462.4 |
| rs770769 | 11 | 43305863 | A | -0.013 | 0.002 | 1.1E-10 | 41.6 |
| rs2959101 | 11 | 46018139 | T | -0.012 | 0.002 | 5.7E-09 | 33.9 |
| rs4752829 | 11 | 47396654 | A | -0.013 | 0.002 | 2.6E-11 | 44.4 |
| rs198444 | 11 | 61505168 | A | -0.015 | 0.002 | 1.7E-14 | 58.8 |
| rs1002126 | 11 | 73104196 | A | -0.012 | 0.002 | 3.3E-10 | 39.5 |
| rs6592145 | 11 | 83536043 | T | -0.012 | 0.002 | 4.3E-10 | 39.0 |
| rs2155413 | 11 | 84634790 | A | -0.022 | 0.002 | 3.8E-28 | 121.0 |
| rs17218455 | 12 | 106716441 | T | -0.012 | 0.002 | 2.8E-10 | 39.8 |
| rs4766878 | 12 | 118236577 | T | -0.014 | 0.002 | 5.2E-12 | 47.6 |
| rs12308700 | 12 | 131157564 | A | -0.014 | 0.002 | 6.0E-12 | 47.3 |
| rs905224 | 12 | 133684321 | T | -0.013 | 0.002 | 1.3E-10 | 41.4 |
| rs61914291 | 12 | 14043113 | A | -0.020 | 0.002 | 3.7E-25 | 107.4 |
| rs144311425 | 12 | 22555028 | T | -0.018 | 0.002 | 6.0E-20 | 83.6 |
| rs11046546 | 12 | 22855267 | A | -0.012 | 0.002 | 3.2E-09 | 35.1 |
| rs11048611 | 12 | 26761513 | T | 0.013 | 0.002 | 1.7E-10 | 40.8 |
| rs1560634 | 12 | 31563280 | A | -0.011 | 0.002 | 1.3E-08 | 32.3 |
| rs11181913 | 12 | 43574200 | A | 0.012 | 0.002 | 1.3E-09 | 36.7 |
| rs11183201 | 12 | 46170982 | T | 0.018 | 0.002 | 1.3E-19 | 82.1 |
| rs3138142 | 12 | 56115585 | T | 0.045 | 0.002 | 7.0E-115 | 519.0 |
| rs12322902 | 12 | 57513866 | A | -0.013 | 0.002 | 2.3E-11 | 44.7 |
| rs12230919 | 12 | 71281530 | A | 0.017 | 0.002 | 5.2E-17 | 70.3 |
| rs117821803 | 12 | 96194586 | T | 0.015 | 0.002 | 2.2E-14 | 58.3 |
| rs9513696 | 13 | 100693002 | A | -0.029 | 0.002 | 3.9E-49 | 217.1 |
| rs138364921 | 13 | 101109158 | C | 0.012 | 0.002 | 5.0E-09 | 34.2 |
| rs587289 | 13 | 21596183 | A | 0.012 | 0.002 | 6.0E-10 | 38.3 |
| rs34961350 | 13 | 28991902 | C | -0.020 | 0.002 | 1.6E-24 | 104.5 |
| rs45502300 | 13 | 36246512 | A | 0.016 | 0.002 | 2.0E-16 | 67.6 |
| rs2038881 | 13 | 50135177 | A | 0.021 | 0.002 | 9.1E-27 | 114.7 |
| rs2325024 | 13 | 67727334 | T | 0.012 | 0.002 | 2.0E-09 | 35.9 |
| rs3850060 | 13 | 80518205 | T | 0.014 | 0.002 | 6.5E-13 | 51.7 |
| rs9547035 | 13 | 85573496 | T | 0.019 | 0.002 | 2.2E-22 | 94.7 |
| rs72634624 | 13 | 85823201 | A | 0.011 | 0.002 | 3.4E-08 | 30.5 |
| rs9517544 | 13 | 99666142 | T | -0.012 | 0.002 | 2.1E-09 | 35.9 |
| rs35337422 | 14 | 104407243 | A | 0.016 | 0.002 | 7.9E-16 | 64.9 |
| rs17522122 | 14 | 33302882 | T | 0.020 | 0.002 | 5.5E-24 | 102.0 |
| rs34217772 | 14 | 42273570 | C | 0.019 | 0.002 | 4.9E-23 | 97.7 |
| rs2855530 | 14 | 54421917 | C | -0.027 | 0.002 | 5.2E-42 | 184.4 |
| rs928109 | 14 | 57279583 | T | -0.011 | 0.002 | 8.6E-09 | 33.1 |
| rs10151339 | 14 | 60813416 | T | -0.020 | 0.002 | 5.8E-25 | 106.5 |
| rs75316250 | 14 | 63783391 | A | -0.011 | 0.002 | 7.5E-09 | 33.4 |
| rs73294458 | 14 | 74933162 | A | 0.018 | 0.002 | 9.6E-17 | 69.1 |
| rs56284621 | 14 | 89429230 | T | 0.014 | 0.002 | 2.0E-12 | 49.5 |
| rs12590449 | 14 | 92629979 | T | 0.013 | 0.002 | 1.3E-11 | 45.8 |
| rs524952 | 15 | 35005886 | A | -0.055 | 0.002 | 1.4E-171 | 779.7 |
| rs1055356 | 15 | 35148102 | T | 0.015 | 0.002 | 1.6E-14 | 59.0 |
| rs8038122 | 15 | 40941242 | A | 0.011 | 0.002 | 5.2E-09 | 34.1 |
| rs2017765 | 15 | 48749703 | T | 0.018 | 0.002 | 7.6E-21 | 87.7 |
| rs7162310 | 15 | 63571234 | T | 0.020 | 0.002 | 5.0E-24 | 102.2 |
| rs893820 | 15 | 74229603 | T | 0.016 | 0.002 | 3.6E-16 | 66.4 |
| rs2046144 | 15 | 79074562 | A | -0.012 | 0.002 | 2.2E-09 | 35.8 |
| rs1961579 | 15 | 79380516 | A | -0.033 | 0.002 | 6.3E-64 | 284.9 |
| rs6497041 | 15 | 93740207 | A | -0.012 | 0.002 | 7.3E-10 | 37.9 |
| rs12440787 | 15 | 95154389 | A | -0.012 | 0.002 | 3.3E-10 | 39.5 |
| rs1213321 | 16 | 10077586 | A | 0.012 | 0.002 | 8.7E-10 | 37.6 |
| rs710902 | 16 | 1376510 | T | 0.015 | 0.002 | 1.0E-12 | 50.8 |
| rs7185575 | 16 | 71672569 | A | 0.013 | 0.002 | 6.5E-11 | 42.7 |
| rs13336069 | 16 | 7272395 | T | 0.014 | 0.002 | 4.0E-13 | 52.6 |
| rs79296018 | 16 | 7452481 | T | 0.017 | 0.002 | 2.6E-18 | 76.1 |
| rs17648524 | 16 | 7459683 | C | -0.044 | 0.002 | 2.5E-109 | 493.5 |
| rs4635359 | 16 | 80537760 | A | -0.019 | 0.002 | 1.6E-21 | 90.7 |
| rs7188775 | 16 | 8196544 | A | 0.015 | 0.002 | 9.5E-15 | 60.0 |
| rs2908972 | 17 | 11407259 | A | -0.031 | 0.002 | 3.7E-57 | 253.9 |
| rs11650808 | 17 | 14116814 | C | 0.013 | 0.002 | 2.4E-10 | 40.1 |
| rs62070231 | 17 | 31229597 | C | -0.023 | 0.002 | 6.8E-32 | 138.1 |
| rs35497503 | 17 | 37620627 | T | 0.016 | 0.002 | 6.5E-16 | 65.3 |
| rs8081968 | 17 | 3984117 | T | -0.013 | 0.002 | 1.5E-11 | 45.5 |
| rs4792863 | 17 | 43563358 | A | 0.013 | 0.002 | 2.4E-09 | 35.6 |
| rs55634267 | 17 | 47283815 | T | 0.025 | 0.002 | 2.8E-36 | 158.2 |
| rs1963456 | 17 | 54715143 | T | 0.023 | 0.002 | 2.9E-31 | 135.3 |
| rs11656528 | 17 | 55546968 | A | -0.014 | 0.002 | 1.3E-12 | 50.3 |
| rs3785837 | 17 | 59468942 | A | -0.016 | 0.002 | 7.9E-15 | 60.4 |
| rs4793501 | 17 | 68718734 | T | -0.017 | 0.002 | 2.2E-17 | 72.0 |
| rs9038 | 17 | 75495397 | T | 0.012 | 0.002 | 2.7E-10 | 39.9 |
| rs60750901 | 17 | 75925242 | C | 0.012 | 0.002 | 4.6E-09 | 34.4 |
| rs4792058 | 17 | 7889206 | A | 0.011 | 0.002 | 8.9E-09 | 33.1 |
| rs67050149 | 17 | 79557043 | A | -0.023 | 0.002 | 3.9E-31 | 134.7 |
| rs4789729 | 17 | 80146089 | A | 0.012 | 0.002 | 2.0E-09 | 36.0 |
| rs12957702 | 18 | 25635271 | C | -0.015 | 0.002 | 1.2E-13 | 55.0 |
| rs10221329 | 18 | 39203393 | A | -0.012 | 0.002 | 6.2E-10 | 38.3 |
| rs11665106 | 18 | 42362589 | A | -0.012 | 0.002 | 3.1E-09 | 35.1 |
| rs8086981 | 18 | 42892099 | T | 0.020 | 0.002 | 1.2E-23 | 100.5 |
| rs12965607 | 18 | 47391025 | T | 0.027 | 0.002 | 3.3E-42 | 185.3 |
| rs9646588 | 18 | 62990567 | A | -0.012 | 0.002 | 9.4E-10 | 37.4 |
| rs1346171 | 18 | 6452548 | A | -0.014 | 0.002 | 2.2E-13 | 53.9 |
| rs734559 | 18 | 72179579 | A | 0.015 | 0.002 | 8.0E-14 | 55.8 |
| rs113254113 | 19 | 13191646 | T | 0.012 | 0.002 | 2.8E-09 | 35.3 |
| rs57436187 | 19 | 19429915 | T | -0.018 | 0.002 | 1.5E-20 | 86.4 |
| rs429358 | 19 | 45411941 | T | -0.013 | 0.002 | 4.5E-11 | 43.4 |
| rs77128495 | 19 | 48533700 | T | 0.019 | 0.002 | 3.3E-21 | 89.4 |
| rs351973 | 19 | 795457 | A | -0.013 | 0.002 | 3.1E-09 | 35.1 |
| rs10416763 | 19 | 8232734 | T | 0.014 | 0.002 | 1.2E-12 | 50.5 |
| rs2570495 | 2 | 104443942 | T | 0.013 | 0.002 | 7.7E-11 | 42.3 |
| rs111259917 | 2 | 1104737 | T | -0.011 | 0.002 | 4.4E-08 | 30.0 |
| rs10205158 | 2 | 144538706 | C | -0.011 | 0.002 | 1.2E-08 | 32.4 |
| rs7349306 | 2 | 146941510 | A | -0.028 | 0.002 | 1.1E-46 | 205.8 |
| rs2656321 | 2 | 149073434 | T | -0.011 | 0.002 | 8.2E-09 | 33.2 |
| rs2352096 | 2 | 157376170 | T | -0.019 | 0.002 | 5.5E-23 | 97.5 |
| rs72772496 | 2 | 16430349 | T | 0.013 | 0.002 | 8.6E-10 | 37.6 |
| rs62183776 | 2 | 172858117 | T | 0.021 | 0.002 | 1.6E-25 | 109.0 |
| rs2573081 | 2 | 178828507 | C | 0.030 | 0.002 | 2.8E-53 | 236.1 |
| rs6736760 | 2 | 184347376 | A | -0.012 | 0.002 | 6.8E-09 | 33.6 |
| rs6705199 | 2 | 208820252 | A | 0.011 | 0.002 | 4.4E-08 | 30.0 |
| rs300751 | 2 | 209063 | A | -0.016 | 0.002 | 1.2E-16 | 68.7 |
| rs121908120 | 2 | 219755011 | A | -0.012 | 0.002 | 4.4E-10 | 38.9 |
| rs2343240 | 2 | 233087483 | T | -0.013 | 0.002 | 2.7E-11 | 44.4 |
| rs3906715 | 2 | 233315444 | A | -0.013 | 0.002 | 1.2E-10 | 41.4 |
| rs1550094 | 2 | 233385396 | A | 0.041 | 0.002 | 3.3E-83 | 373.5 |
| rs62187839 | 2 | 241748185 | T | 0.011 | 0.002 | 4.6E-08 | 29.9 |
| rs11127261 | 2 | 30475153 | A | -0.014 | 0.002 | 1.4E-12 | 50.2 |
| rs77791728 | 2 | 36608132 | T | 0.011 | 0.002 | 4.8E-08 | 29.8 |
| rs75120545 | 2 | 44271496 | T | -0.019 | 0.002 | 1.5E-21 | 90.9 |
| rs13000834 | 2 | 45139807 | T | 0.015 | 0.002 | 4.5E-15 | 61.4 |
| rs9309272 | 2 | 56095366 | T | -0.022 | 0.002 | 4.8E-28 | 120.6 |
| rs4380284 | 2 | 56142098 | A | 0.015 | 0.002 | 3.5E-14 | 57.4 |
| rs11555696 | 2 | 74588605 | A | 0.013 | 0.002 | 5.4E-10 | 38.5 |
| rs6547257 | 2 | 79918454 | A | -0.012 | 0.002 | 1.2E-08 | 32.4 |
| rs4911411 | 20 | 32715842 | T | -0.015 | 0.002 | 4.3E-14 | 57.0 |
| rs2425091 | 20 | 34306723 | A | 0.014 | 0.002 | 5.8E-12 | 47.4 |
| rs11696781 | 20 | 58348589 | A | -0.013 | 0.002 | 9.9E-11 | 41.8 |
| rs2253829 | 20 | 62373079 | C | -0.011 | 0.002 | 6.4E-09 | 33.7 |
| rs235770 | 20 | 6761765 | T | -0.021 | 0.002 | 4.8E-27 | 116.0 |
| rs2229741 | 21 | 16340289 | T | -0.019 | 0.002 | 6.0E-22 | 92.7 |
| rs62218223 | 21 | 16536320 | A | -0.015 | 0.002 | 1.0E-14 | 59.9 |
| rs117123217 | 21 | 30910628 | A | -0.012 | 0.002 | 2.9E-09 | 35.3 |
| rs2150458 | 21 | 47377296 | A | 0.021 | 0.002 | 1.2E-25 | 109.6 |
| rs9608575 | 22 | 27486722 | A | -0.011 | 0.002 | 2.4E-08 | 31.2 |
| rs4820079 | 22 | 32879617 | T | -0.011 | 0.002 | 7.6E-09 | 33.4 |
| rs6519240 | 22 | 41416557 | T | -0.019 | 0.002 | 3.3E-21 | 89.3 |
| rs2294915 | 22 | 44340904 | T | -0.011 | 0.002 | 4.1E-08 | 30.1 |
| rs9330813 | 22 | 46364161 | A | -0.023 | 0.002 | 1.0E-26 | 114.4 |
| rs2116693 | 3 | 107761411 | T | 0.012 | 0.002 | 3.2E-09 | 35.1 |
| rs1710882 | 3 | 11064711 | T | -0.012 | 0.002 | 4.2E-09 | 34.5 |
| rs251449 | 3 | 118758706 | T | -0.012 | 0.002 | 5.4E-10 | 38.5 |
| rs78559200 | 3 | 127383657 | T | 0.016 | 0.002 | 1.8E-15 | 63.3 |
| rs6785073 | 3 | 141139330 | A | 0.026 | 0.002 | 3.2E-38 | 167.1 |
| rs62275408 | 3 | 146369080 | A | 0.011 | 0.002 | 4.0E-08 | 30.2 |
| rs2603783 | 3 | 16082811 | T | -0.013 | 0.002 | 2.9E-11 | 44.2 |
| rs62283809 | 3 | 171820211 | T | -0.013 | 0.002 | 7.3E-11 | 42.4 |
| rs6445046 | 3 | 171933252 | T | 0.011 | 0.002 | 8.2E-09 | 33.2 |
| rs4618261 | 3 | 178667299 | A | -0.012 | 0.002 | 2.6E-09 | 35.5 |
| rs4858582 | 3 | 24219521 | A | 0.019 | 0.002 | 1.3E-21 | 91.3 |
| rs7622604 | 3 | 29450982 | T | -0.013 | 0.002 | 7.2E-11 | 42.5 |
| rs9830029 | 3 | 33262129 | A | -0.015 | 0.002 | 2.0E-13 | 54.1 |
| rs12492279 | 3 | 41289031 | T | 0.018 | 0.002 | 2.7E-19 | 80.6 |
| rs2587916 | 3 | 4185379 | T | 0.012 | 0.002 | 2.1E-09 | 35.9 |
| rs111533129 | 3 | 49437600 | C | -0.015 | 0.002 | 2.1E-14 | 58.4 |
| rs14165 | 3 | 53847408 | A | 0.018 | 0.002 | 7.0E-19 | 78.8 |
| rs35557787 | 3 | 58234536 | T | 0.012 | 0.002 | 2.2E-09 | 35.7 |
| rs35667547 | 3 | 64547477 | C | -0.012 | 0.002 | 2.1E-08 | 31.4 |
| rs60743220 | 3 | 68945153 | T | 0.015 | 0.002 | 6.1E-15 | 60.9 |
| rs178597 | 3 | 8196941 | T | 0.025 | 0.002 | 2.6E-36 | 158.3 |
| rs6790699 | 3 | 85624189 | A | 0.016 | 0.002 | 1.9E-15 | 63.1 |
| rs9824877 | 3 | 98896242 | A | -0.018 | 0.002 | 7.0E-20 | 83.3 |
| rs13080329 | 3 | 99463810 | T | 0.011 | 0.002 | 4.9E-08 | 29.8 |
| rs13107325 | 4 | 103188709 | T | 0.015 | 0.002 | 1.2E-13 | 55.0 |
| rs28706824 | 4 | 120673378 | A | 0.014 | 0.002 | 4.2E-13 | 52.5 |
| rs11940147 | 4 | 149974078 | T | 0.011 | 0.002 | 6.6E-09 | 33.7 |
| rs6816915 | 4 | 166800976 | A | 0.011 | 0.002 | 1.6E-08 | 32.0 |
| rs147792504 | 4 | 174336590 | A | -0.014 | 0.002 | 2.8E-10 | 39.8 |
| rs4446359 | 4 | 183034387 | A | 0.011 | 0.002 | 1.2E-08 | 32.5 |
| rs28526098 | 4 | 44933151 | A | -0.013 | 0.002 | 4.6E-11 | 43.3 |
| rs72659078 | 4 | 79563579 | A | 0.011 | 0.002 | 3.1E-08 | 30.6 |
| rs78054636 | 4 | 80873617 | T | 0.021 | 0.002 | 1.1E-26 | 114.3 |
| rs1458040 | 4 | 81209680 | A | 0.011 | 0.002 | 3.3E-08 | 30.5 |
| rs72661723 | 4 | 81627784 | A | -0.012 | 0.002 | 3.4E-09 | 34.9 |
| rs74764079 | 4 | 81952637 | A | -0.027 | 0.002 | 4.9E-41 | 180.0 |
| rs2120817 | 4 | 82376938 | A | 0.016 | 0.002 | 7.3E-16 | 65.0 |
| rs13118477 | 4 | 89704367 | A | -0.014 | 0.002 | 4.2E-12 | 48.0 |
| rs154001 | 5 | 127685135 | T | -0.011 | 0.002 | 3.3E-08 | 30.5 |
| rs246073 | 5 | 140317371 | T | 0.013 | 0.002 | 1.1E-10 | 41.6 |
| rs7714980 | 5 | 143578326 | A | -0.013 | 0.002 | 1.1E-10 | 41.7 |
| rs62385438 | 5 | 158497639 | T | -0.015 | 0.002 | 1.5E-13 | 54.6 |
| rs9313328 | 5 | 166012675 | A | 0.011 | 0.002 | 4.8E-08 | 29.8 |
| rs6875105 | 5 | 173054917 | T | -0.012 | 0.002 | 3.8E-10 | 39.2 |
| rs12186577 | 5 | 178359989 | T | 0.016 | 0.002 | 2.1E-15 | 63.0 |
| rs75150349 | 5 | 45491724 | T | -0.012 | 0.002 | 1.8E-09 | 36.2 |
| rs1309551 | 5 | 64288656 | T | -0.015 | 0.002 | 3.6E-14 | 57.4 |
| rs4428386 | 5 | 71707274 | A | 0.012 | 0.002 | 3.6E-10 | 39.3 |
| rs6884773 | 5 | 87715465 | A | 0.017 | 0.002 | 4.0E-18 | 75.3 |
| rs9484245 | 6 | 100193003 | A | -0.012 | 0.002 | 5.4E-10 | 38.5 |
| rs1064583 | 6 | 116446576 | A | 0.019 | 0.002 | 1.6E-22 | 95.4 |
| rs12193446 | 6 | 129820038 | A | -0.062 | 0.002 | 1.0E-200 | 990.0 |
| rs9375702 | 6 | 130384187 | T | -0.013 | 0.002 | 3.8E-11 | 43.7 |
| rs2328160 | 6 | 141314296 | A | -0.011 | 0.002 | 1.7E-08 | 31.8 |
| rs9478898 | 6 | 151320648 | A | 0.014 | 0.002 | 1.2E-12 | 50.5 |
| rs2143358 | 6 | 163981387 | A | -0.011 | 0.002 | 1.6E-08 | 31.9 |
| rs880095 | 6 | 164249854 | T | 0.011 | 0.002 | 5.0E-08 | 29.7 |
| rs4527692 | 6 | 21160447 | T | 0.012 | 0.002 | 1.2E-09 | 37.1 |
| rs9366426 | 6 | 22064639 | T | 0.023 | 0.002 | 3.2E-32 | 139.6 |
| rs10458141 | 6 | 2439311 | A | -0.015 | 0.002 | 4.5E-14 | 57.0 |
| rs1150691 | 6 | 28168033 | A | 0.020 | 0.002 | 8.2E-24 | 101.2 |
| rs3130100 | 6 | 33283766 | T | -0.011 | 0.002 | 1.4E-08 | 32.2 |
| rs11751920 | 6 | 34655818 | C | 0.012 | 0.002 | 1.7E-09 | 36.3 |
| rs7758685 | 6 | 43825266 | A | 0.012 | 0.002 | 2.0E-09 | 36.0 |
| rs2076309 | 6 | 50789451 | T | 0.020 | 0.002 | 2.0E-25 | 108.6 |
| rs2326838 | 6 | 6901663 | A | -0.018 | 0.002 | 4.1E-19 | 79.8 |
| rs2881952 | 6 | 72320145 | A | -0.012 | 0.002 | 1.0E-09 | 37.3 |
| rs7744813 | 6 | 73643289 | A | -0.048 | 0.002 | 6.0E-132 | 597.5 |
| rs1891394 | 6 | 73649583 | T | 0.014 | 0.002 | 3.2E-12 | 48.6 |
| rs533632 | 6 | 8214304 | A | 0.012 | 0.002 | 1.8E-09 | 36.2 |
| rs1118543 | 6 | 84380492 | C | 0.018 | 0.002 | 8.2E-21 | 87.6 |
| rs2181653 | 6 | 9098630 | T | -0.011 | 0.002 | 1.4E-08 | 32.2 |
| rs1487441 | 6 | 98553894 | A | -0.014 | 0.002 | 2.2E-12 | 49.3 |
| rs2106177 | 7 | 127072695 | T | -0.011 | 0.002 | 1.3E-08 | 32.3 |
| rs58287690 | 7 | 151122699 | T | 0.017 | 0.002 | 1.7E-15 | 63.4 |
| rs34634437 | 7 | 158734813 | T | 0.012 | 0.002 | 4.7E-10 | 38.8 |
| rs12234576 | 7 | 158928375 | A | 0.022 | 0.002 | 3.1E-29 | 126.0 |
| rs59619245 | 7 | 2068630 | T | 0.015 | 0.002 | 3.6E-14 | 57.4 |
| rs2390536 | 7 | 21485397 | A | -0.012 | 0.002 | 1.7E-09 | 36.3 |
| rs7801660 | 7 | 39072473 | T | 0.011 | 0.002 | 4.6E-08 | 29.9 |
| rs10258656 | 7 | 47763485 | A | 0.014 | 0.002 | 1.6E-12 | 49.9 |
| rs7786560 | 7 | 7252249 | A | 0.015 | 0.002 | 1.4E-13 | 54.7 |
| rs6954078 | 7 | 82435878 | T | -0.015 | 0.002 | 8.8E-15 | 60.1 |
| rs1228859 | 7 | 84006549 | T | -0.011 | 0.002 | 1.7E-08 | 31.8 |
| rs854099 | 7 | 95320949 | T | -0.011 | 0.002 | 9.8E-09 | 32.9 |
| rs11771139 | 7 | 99736059 | A | -0.014 | 0.002 | 7.1E-13 | 51.5 |
| rs10100265 | 8 | 10633159 | A | 0.016 | 0.002 | 5.1E-17 | 70.3 |
| rs6988919 | 8 | 121630740 | C | 0.018 | 0.002 | 2.5E-19 | 80.8 |
| rs57594397 | 8 | 22475657 | T | 0.013 | 0.002 | 4.8E-11 | 43.3 |
| rs16890057 | 8 | 40726582 | A | 0.029 | 0.002 | 5.0E-48 | 212.0 |
| rs166928 | 8 | 53350287 | A | -0.016 | 0.002 | 2.5E-16 | 67.1 |
| rs1050403 | 8 | 56985814 | T | -0.014 | 0.002 | 4.8E-12 | 47.7 |
| rs72621438 | 8 | 60178580 | C | -0.043 | 0.002 | 2.8E-106 | 479.5 |
| rs4738813 | 8 | 61595671 | T | 0.019 | 0.002 | 6.0E-21 | 88.2 |
| rs4738095 | 8 | 71423746 | A | -0.018 | 0.002 | 5.3E-19 | 79.3 |
| rs9650252 | 8 | 75731730 | T | 0.012 | 0.002 | 2.4E-10 | 40.1 |
| rs13268738 | 8 | 87673711 | T | 0.012 | 0.002 | 2.0E-10 | 40.5 |
| rs10760673 | 9 | 101878622 | A | -0.015 | 0.002 | 8.9E-15 | 60.1 |
| rs10978697 | 9 | 109763587 | A | 0.016 | 0.002 | 9.3E-16 | 64.6 |
| rs1887521 | 9 | 112553003 | T | -0.011 | 0.002 | 1.5E-08 | 32.1 |
| rs2072438 | 9 | 123651301 | T | -0.011 | 0.002 | 1.3E-08 | 32.3 |
| rs10491745 | 9 | 12679607 | T | -0.011 | 0.002 | 9.5E-09 | 32.9 |
| rs2998190 | 9 | 127966535 | T | -0.012 | 0.002 | 6.0E-10 | 38.3 |
| rs11103381 | 9 | 139097773 | A | -0.017 | 0.002 | 5.9E-15 | 60.9 |
| rs1340044 | 9 | 18362105 | A | -0.025 | 0.002 | 2.6E-36 | 158.4 |
| rs855719 | 9 | 33817617 | T | -0.013 | 0.002 | 1.1E-10 | 41.7 |
| rs11145422 | 9 | 71755705 | T | 0.018 | 0.002 | 7.3E-20 | 83.2 |
| rs11143764 | 9 | 76648938 | T | -0.014 | 0.002 | 1.1E-12 | 50.7 |
| rs7042950 | 9 | 77149837 | A | 0.021 | 0.002 | 1.6E-25 | 109.0 |

**Abbreviations:** CHR, chromosome; SE, standard error.

## **Table S17. Genetic instruments for axial length**

Variants extracted from Jiang *et al. [19]*

| **SNP** | **CHR** | **Position** | **Effect Allele** | **Beta** | **SE** | ***P*-value** | **F statistic** |
| --- | --- | --- | --- | --- | --- | --- | --- |
| rs2490256 | 1 | 207521901 | A | 0.088 | 0.015 | 3.3E-09 | 34.9 |
| rs11118357 | 1 | 219777721 | A | 0.085 | 0.014 | 4.1E-10 | 39.2 |
| rs10887265 | 10 | 86015573 | C | 0.086 | 0.014 | 5.3E-10 | 38.6 |
| rs11606250 | 11 | 40149300 | A | 0.103 | 0.018 | 7.5E-09 | 33.3 |
| rs3138142 | 12 | 56115585 | T | -0.128 | 0.017 | 3.2E-14 | 57.5 |
| rs634990 | 15 | 35006073 | C | 0.113 | 0.014 | 7.0E-17 | 69.6 |
| rs17648524 | 16 | 7459683 | C | 0.089 | 0.014 | 1.6E-10 | 40.7 |
| rs4793821 | 17 | 54689125 | G | 0.080 | 0.014 | 5.2E-09 | 33.9 |
| rs55754534 | 18 | 47433745 | C | 0.114 | 0.019 | 3.5E-09 | 34.8 |
| rs62182444 | 2 | 172812544 | A | -0.097 | 0.016 | 6.5E-10 | 38.4 |
| rs2853447 | 2 | 233389918 | G | -0.094 | 0.017 | 1.4E-08 | 32.3 |
| rs1353386 | 4 | 81947080 | C | -0.100 | 0.018 | 2.7E-08 | 31.1 |
| rs12193446 | 6 | 129820038 | G | -0.173 | 0.023 | 7.1E-14 | 56.0 |
| rs7744813 | 6 | 73643289 | A | 0.103 | 0.014 | 4.3E-14 | 56.9 |
| rs10089517 | 8 | 60178721 | A | -0.095 | 0.014 | 2.2E-11 | 45.0 |

**Abbreviations:** CHR, chromosome; SE, standard error.

## **Table S18: Mendelian Randomization (MR) analyses for the causal effect of retinal vascular phenotypes on Alzheimer's Disease risk.**

| **Analysis** | **Causal Estimate (95% CI)** | ***P-*value** |
| --- | --- | --- |
| **Genetically predicted AT – AD** | | |
| IVW | 0.99 (0.86, 1.14) | 0.94 |
| MR-Egger | 1.38 (0.94, 2.02) | 0.09 |
| MR-Egger Intercept* | -0.01 (-0.02, 0.008) | 0.07 |
| Weighted Median | 1.21 (1.00, 1.44) | **0.04** |
| Weighted Mode | 1.26 (0.92, 1.74) | 0.15 |
| **Genetically predicted VT – AD** | | |
| IVW | 1.02 (0.61, 1.69) | 0.92 |
| MR-Egger | 1.43 (0.46, 4.41) | 0.54 |
| MR-Egger Intercept* | -0.0071 (-0.028, 0.014) | 0.52 |
| Weighted Median | 1.33 (0.76, 2.33) | 0.30 |
| Weighted Mode | 1.35 (0.73, 2.46) | 0.34 |
| **Genetically predicted VW – AD** | | |
| IVW | 0.99 (0.98, 1.01) | 0.78 |
| MR-Egger | 1.01 (0.93, 1.10) | 0.71 |
| MR-Egger Intercept* | -0.01 (-0.058, 0.037) | 0.66 |
| Weighted Median | 0.98 (0.96, 1.00) | 0.26 |
| Weighted Mode | 0.98 (0.94, 1.02) | 0.40 |
| **Genetically predicted FD – AD** | | |
| IVW | 0.10 (0.0004, 12.29) | 0.35 |
| MR-Egger | 0.0009 (0.0, 13.07) | 0.38 |
| MR-Egger Intercept* | 0.025 (-0.019, 0.07) | 0.46 |
| Weighted Median | 0.064 (0.0004, 9.15) | 0.27 |
| Weighted Mode | 0.064 (0.0004, 8.74) | 0.38 |

**Abbreviations:** AD=Alzheimer’s Disease; CI=confidence interval; IVW=inverse-variance weighted; AT= arteriolar tortuosity; VT= venular tortuosity; VW= venular width. Causal estimates are odds ratios for Alzheimer’s Disease risk except MR-Egger Intercept*.

## **Table S19. Levels of heterogeneity in MR analysis of exposure to ocular traits on Alzheimer’s disease risk before Steiger filtering.**

| **Exposure** | **MR Methods** | **Outcome: Alzheimer’s Disease** | | |
| --- | --- | --- | --- | --- |
|  |  | **Q** | **df** | **P value** |
| mRNFL thickness | MR Egger | 18.46 | 22 | 0.67 |
|  | IVW | 18.62 | 23 | 0.72 |
| mGCIPL thickness | MR Egger | 24.86 | 19 | 0.16 |
|  | IVW | 24.89 | 20 | 0.20 |
| Macular thickness | MR Egger | 161.76 | 103 | 0.0001 |
|  | IVW | 161.84 | 104 | 0.0002 |
| Outer nuclear layer | MR Egger | 65.78 | 56 | 0.17 |
|  | IVW | 65.80 | 57 | 0.19 |
| Inner segment layer | MR Egger | 15.08 | 13 | 0.30 |
|  | IVW | 15.20 | 14 | 0.36 |
| Outer segment layer | MR Egger | 19.79 | 31 | 0.94 |
|  | IVW | 20.93 | 32 | 0.93 |
| Arteriolar tortuosity | MR Egger | 94.23 | 80 | 0.13 |
|  | IVW | 98.14 | 81 | 0.09 |
| Venular tortuosity | MR Egger | 22.94 | 14 | 0.06 |
|  | IVW | 23.65 | 15 | 0.07 |
| Venular width | MR Egger | 15.01 | 11 | 0.18 |
|  | IVW | 15.27 | 12 | 0.22 |
| Fractal dimension | MR Egger | 0.64 | 1 | 0.42 |
|  | IVW | 1.90 | 2 | 0.38 |
| Optic disc area | MR Egger | 8.15 | 9 | 0.51 |
|  | IVW | 8.21 | 10 | 0.60 |
| Optic cup area | MR Egger | 45.57 | 38 | 0.18 |
|  | IVW | 45.62 | 39 | 0.21 |
| Vertical cup disc ratio | MR Egger | 20.92 | 15 | 0.13 |
|  | IVW | 21.31 | 16 | 0.16 |

**Abbreviations:** Q = estimate of Cochran’s Q statistic, df = degrees of freedom, IVW = Inverse variance weighted, RNFL= Retinal nerve fibre layer, GCIPL = ganglion cell inner plexiform layer.

## **Table S20. Levels of heterogeneity in MR analysis of AD exposure on ocular traits before Steiger filtering.**

| **MR Outcome** | **Methods** | **MR Exposure (Alzheimer’s Disease)** | | |
| --- | --- | --- | --- | --- |
|  |  | **Q** | **df** | **P value** |
| mRNFL thickness | MR Egger | 33.56 | 18 | **0.014** |
|  | IVW | 36.51 | 19 | **0.009** |
| mGCIPL thickness | MR Egger | 25.67 | 18 | 0.10 |
|  | IVW | 30.81 | 19 | **0.04** |
| Macular thickness | MR Egger | 30.33 | 18 | **0.03** |
|  | IVW | 32.21 | 19 | **0.03** |
| Outer nuclear layer | MR Egger | 25.47 | 18 | 0.11 |
|  | IVW | 25.47 | 19 | 0.14 |
| Inner segment layer | MR Egger | 23.12 | 18 | 0.18 |
|  | IVW | 24.14 | 19 | 0.19 |
| Outer segment layer | MR Egger | 18.60 | 18 | 0.41 |
|  | IVW | 19.63 | 19 | 0.41 |
| Arteriolar Tortuosity | MR Egger | 27.30 | 19 | 0.09 |
|  | IVW | 28.43 | 20 | 0.09 |
| Venular Tortuosity | MR Egger | 26.36 | 19 | 0.12 |
|  | IVW | 26.40 | 20 | 0.15 |
| Venular Width | MR Egger | 28.77 | 19 | 0.06 |
|  | IVW | 30.62 | 20 | 0.06 |
| Fractal dimension | MR Egger | 15.43 | 16 | 0.49 |
|  | IVW | 15.99 | 17 | 0.52 |
| Optic disc area | MR Egger | 34.39 | 17 | **0.007** |
|  | IVW | 38.34 | 18 | **0.003** |
| Optic cup area | MR Egger | 9.24 | 17 | 0.93 |
|  | IVW | 9.24 | 18 | 0.95 |
| Vertical cup disc ratio | MR Egger | 8.88 | 17 | 0.94 |
|  | IVW | 8.89 | 18 | 0.96 |

**Abbreviations:** Q = estimate of Cochran’s Q statistic, df= degrees of freedom, IVW= inverse variance weighted, RNFL= Retinal nerve fibre layer, GCIPL=ganglion cell inner plexiform layer.

## **Table S21. Mendelian Randomization analyses for the causal effect of Alzheimer's disease on retinal nerve fibre layer thickness after Steiger filtering**

The number of instrument variables in each analysis before and after Steiger filtering is indicated in brackets

| **Analysis** | **Estimate (95% CI)** | **P-value** |
| --- | --- | --- |
| **AD genetic risk – mRNFL thickness (20 ® 10 IVs)** | | |
| IVW | **-0.062 (-0.115, -0.008)** | **0.02** |
| MR-Egger | -0.07 (-0.136, -0.005) | 0.06 |
| MR-Egger Intercept | 0.007 (-0.02, 0.04) | 0.67 |
| Weighted Median | **-0.065 (-0.122, -0.007)** | **0.02** |
| Weighted Mode | **-0.064 (-0.119, -0.01)** | **0.04** |
| **AD genetic risk – mGCIPL thickness (20 ® 11 IVs)** | | |
| IVW | **-0.076 (-0.14, -0.006)** | **0.03** |
| MR-Egger | **-0.102 (-0.18, -0.016)** | **0.04** |
| MR-Egger Intercept | 0.022 (-0.02, 0.06) | 0.33 |
| Weighted Median | **-0.093 (-0.16, -0.020)** | **0.01** |
| Weighted Mode | **-0.093 (-0.17, -0.017)** | **0.03** |
| **AD genetic risk – Macular thickness (20 ®5 IVs)** | | |
| IVW | -0.013 (-0.118, 0.09) | 0.80 |
| MR-Egger | -0.022 (-0.153, 0.109) | 0.76 |
| MR-Egger Intercept | 0.01 (-0.086, 0.107) | 0.84 |
| Weighted Median | -0.016 (-0.124, 0.091) | 0.76 |
| Weighted Mode | -0.017 (-0.123, 0.08) | 0.76 |
| **AD genetic risk – ONL thickness (20 ®8 IVs)** | | |
| IVW | -0.06 (-0.13, 0.01) | 0.11 |
| MR-Egger | -0.06 (-0.15, 0.02) | 0.22 |
| MR-Egger Intercept | 0.002 (-0.05, 0.05) | 0.93 |
| Weighted Median | -0.05 (-0.13, 0.02) | 0.16 |
| Weighted Mode | -0.06 (-0.13, 0.02) | 0.20 |
| **AD genetic risk – IS thickness (20 ®20 IVs)** | | |
| IVW | 0.002 (-0.017, 0.020) | 0.86 |
| MR-Egger | -0.004 (-0.027, 0.018) | 0.70 |
| MR-Egger Intercept | 0.003 (-0.004, 0.013) | 0.38 |
| Weighted Median | -0.0004 (-0.018, 0.017) | 0.96 |
| Weighted Mode | -0.0009 (-0.019, 0.017) | 0.92 |
| **AD genetic risk – OS thickness (20 ® 16 IVs)** | | |
| IVW | 0.036 (-0.007, 0.08) | 0.10 |
| MR-Egger | **0.061 (-0.008, 0.11)** | **0.03** |
| MR-Egger Intercept | -0.017 (-0.039, 0.0034) | 0.12 |
| Weighted Median | **0.052 (-0.006, 0.09)** | **0.02** |
| Weighted Mode | 0.053 (-0.005, 0.10) | 0.04 |

**Abbreviations:** AD=Alzheimer’s Disease; CI=confidence interval; IV= instrument variable; IVW=inverse-variance weighted; mRNFL= macular retinal nerve fibre layer; mGCIPL= macular ganglion cell inner plexiform layer, ONL=outer nuclear layer, IS= inner segment, OS= outer segment. Estimates are given for SD change per doubling odds of Alzheimer’s disease genetic risk.

## **Table S22. Levels of heterogeneity in MR analysis of AD exposure on ocular traits after Steiger filtering**

| **MR Outcome** | **Methods** | **MR Exposure (Alzheimer’s Disease)** | | |
| --- | --- | --- | --- | --- |
|  |  | **Q** | **df** | **P value** |
| mRNFL thickness | MR Egger | 1.27 | 8 | 0.99 |
|  | IVW | 1.46 | 9 | 0.99 |
| mGCIPL thickness | MR Egger | 3.90 | 9 | 0.91 |
|  | IVW | 4.95 | 10 | 0.89 |
| Macular thickness | MR Egger | 1.14 | 3 | 0.98 |
|  | IVW | 1.19 | 4 | 0.99 |
| Outer nuclear layer | MR Egger | 1.43 | 6 | 0.96 |
|  | IVW | 1.43 | 7 | 0.98 |
| Inner segment layer | MR Egger | 23.12 | 18 | 0.18 |
|  | IVW | 24.14 | 19 | 0.19 |
| Outer segment layer | MR Egger | 6.36 | 14 | 0.95 |
|  | IVW | 9.06 | 15 | 0.87 |
| Arteriolar tortuosity | MR Egger | 27.30 | 19 | 0.09 |
|  | IVW | 28.43 | 20 | 0.09 |
| Venular tortuosity | MR Egger | 26.36 | 19 | 0.12 |
|  | IVW | 26.40 | 20 | 0.15 |
| Venular width | MR Egger | 0.31 | 5 | 0.99 |
|  | IVW | 0.32 | 6 | 0.99 |
| Fractal dimension | MR Egger | 15.43 | 16 | 0.49 |
|  | IVW | 15.99 | 17 | 0.52 |
| Optic disc area | MR Egger | 34.39 | 17 | **0.007** |
|  | IVW | 38.34 | 18 | **0.003** |
| Optic cup area | MR Egger | 9.24 | 17 | 0.93 |
|  | IVW | 9.24 | 18 | 0.95 |
| Vertical cup disc ratio | MR Egger | 8.88 | 17 | 0.94 |
|  | IVW | 8.89 | 18 | 0.96 |

**Abbreviations:** Q = estimate of Cochran’s Q statistic, df= degrees of freedom, IVW= inverse variance weighted, RNFL= Retinal nerve fibre layer, GCIPL=ganglion cell inner plexiform layer

## **Table S23: Mendelian Randomization (MR) analyses for the causal effect of the retinal phenotypes on Alzheimer's Disease risk**

| **Analysis** | **Estimate (95% CI)** | **P-value** |
| --- | --- | --- |
| **Genetically predicted mRNFL thickness – AD risk** | | |
| IVW | 1.00 (0.98, 1.03) | 0.51 |
| MR-Egger | 1.02 (0.95, 1.09) | 0.55 |
| MR-Egger Intercept* | -0.004 (-0.027, 0.017) | 0.69 |
| Weighted Median | 1.01 (0.98, 1.04) | 0.45 |
| Weighted Mode | 1.01 (0.97, 1.06) | 0.43 |
| **Genetically predicted mGCIPL thickness – AD risk** | | |
| IVW | 1.01 (0.99, 1.03) | 0.23 |
| MR-Egger | 1.00 (0.94, 1.07) | 0.79 |
| MR-Egger Intercept* | 0.0018 (-0.012, 0.026) | 0.88 |
| Weighted Median | 1.01 (0.98, 1.04) | 0.24 |
| Weighted Mode | 1.01 (0.97, 1.06) | 0.42 |
| **Genetically predicted Macular thickness– AD risk** | | |
| IVW | 1.00 (0.99, 1.00) | 0.88 |
| MR-Egger | 1.00 (0.98, 1.02) | 0.79 |
| MR-Egger Intercept* | 0.0018 (-0.022, 0.026) | 0.88 |
| Weighted Median | 1.00 (0.99, 1.00) | 0.98 |
| Weighted Mode | 0.99 (0.98, 1.01) | 0.84 |
| **Genetically predicted ONL thickness– AD risk** | | |
| IVW | 1.00 (0.99, 1.01) | 0.99 |
| MR-Egger | 1.00 (0.98, 1.02) | 0.90 |
| MR-Egger Intercept* | -0.0006 (-0.01, 0.009) | 0.89 |
| Weighted Median | 0.99 (0.98, 1.01) | 0.78 |
| Weighted Mode | 0.99 (0.97, 1.01) | 0.72 |
| **Genetically predicted IS thickness– AD risk** | | |
| IVW | 0.96 (0.87, 1.06) | 0.45 |
| MR-Egger | 0.91 (0.64, 1.29) | 0.61 |
| MR-Egger Intercept* | 0.005 (-0.026, 0.036) | 0.75 |
| Weighted Median | 0.96 (0.84, 1.09) | 0.59 |
| Weighted Mode | 0.96 (0.81, 1.13) | 0.65 |
| **Genetically predicted OS thickness– AD risk** | | |
| IVW | 1.00 (0.98, 1.02) | 0.48 |
| MR-Egger | 1.03 (0.97, 1.10) | 0.22 |
| MR-Egger Intercept* | -0.009 (-0.027, 0.008) | 0.29 |
| Weighted Median | 1.00 (0.97, 1.03) | 0.89 |
| Weighted Mode | 0.98 (0.93, 1.04) | 0.68 |

**Abbreviations:** AD=Alzheimer’s Disease; CI=confidence interval; IVW=inverse-variance weighted; RNFL= retinal nerve fibre layer; GCIPL= ganglion cell inner plexiform layer, ONL=outer nuclear layer, IS= inner segment, OS= outer segment. Causal estimates are odds ratios for Alzheimer’s Disease risk except MR-Egger Intercept*.

## **Table S24. The causal effects of Alzheimer's disease on optic disc morphology based on univariable Mendelian randomization.**

| **Analysis** | **Estimate (95% CI)** | **P-value** |
| --- | --- | --- |
| **AD genetic risk – optic disc area** | | |
| IVW | -0.009 (-0.019, 0.0005) | 0.06 |
| MR-Egger | -0.0045 (-0.016, 0.007) | 0.48 |
| MR-Egger Intercept | -0.003 (-0.007, 0.0013) | 0.19 |
| Weighted Median | -0.006 (-0.013, 0.001) | 0.11 |
| Weighted Mode | -0.007 (-0.015, 0.0003) | 0.07 |
| **AD genetic risk – optic cup area** | | |
| IVW | 0.0002 (-0.003, 0.004) | 0.88 |
| MR-Egger | 0.0002 (-0.005, 0.005) | 0.92 |
| MR-Egger Intercept | 0.00003 (-0.002, 0.002) | 0.96 |
| Weighted Median | 0.0003 (-0.004, 0.004) | 0.88 |
| Weighted Mode | 0.0005 (-0.004, 0.005) | 0.83 |
| **AD genetic risk – VCDR** | | |
| IVW | -0.001 (-0.003, 0.001) | 0.37 |
| MR-Egger | -0.0009 (-0.003, 0.001) | 0.52 |
| MR-Egger Intercept | -0.00004 (-0.001, 0.001) | 0.93 |
| Weighted Median | -0.0007 (-0.003, 0.002) | 0.57 |
| Weighted Mode | -0.0009 (-0.003, 0.002) | 0.47 |

AD=Alzheimer’s Disease; CI=confidence interval; IVW=inverse-variance weighted; VCDR=vertical cup-disk ratio. Estimates are given for SD change per doubling odds of Alzheimer’s disease genetic risk.

## **Table S25: The direct causal effects of optic disc area, refractive error and axial length on Alzheimer’s Disease risk using multivariable Mendelian randomization.**

| **Exposures** | **OR (95% CI)** | ***P-*value** | **SNPs** | **F-statistic** | **Q-statistic (p-value)** |
| --- | --- | --- | --- | --- | --- |
| **Model 1** |  |  |  |  |  |
| Optic disc area | 0·75 (0·63, 0·89) | 0·003 | 26 | 41·62 | 18.87 (0.70) |
| Axial length | 0·98 (0·90, 1·05) | 0·60 | 26 | 24·37 |  |
| **Model 2** |  |  |  |  |  |
| Optic disc area | 0·67 (0·36, 1·25) | 0·21 | 291 | 4·92 | 4212·13 (0·00) |
| Refractive error | 1·22 (0·78, 1·90) | 0·36 | 291 | 54·22 |  |

Note: Model 1 represents the estimation of direct causal effects of optic disc area and axial length on Alzheimer’s disease risk, whereas Model 2 represents the estimation of direct causal effects of optic disc area and refractive error on Alzheimer’s disease risk. The F-statistic reports the conditional F-statistic for each SNP and exposure, accounting for the testing of the strength of the instruments in the model. Q-statistic is the modified form of Cochran’s Q statistic that is used to test for horizontal pleiotropy.

Abbreviation: OR: odds ratio, CI: confidence interval, SNPs: single-nucleotide polymorphisms.

## **Table S26. The causal effects of Alzheimer's disease on ocular features based on univariable Mendelian randomization for other AD GWASs.**

| **Outcome** | **MR methods** | **AD GWAS: Bellenguez et al (2022)** | | | **AD GWAS: Jansen et al (2019)** | | | **AD GWAS: Lambert et al (2013)** | | |
| --- | --- | --- | --- | --- | --- | --- | --- | --- | --- | --- |
|  |  | **SNPs** | **β (95%CI)** | **P-value** | **SNPs** | **β (95%CI)** | **P-value** | **SNPs** | **β (95%CI)** | **P-value** |
| mRNFL | Inverse variance weighted | 74 | -0.054 (-0.175, 0.067) | 0.380 | 26 | 0.314 (-0.287, 0.915) | 0.306 | 21 | -0.043 (-0.112, 0.026) | 0.218 |
| mRNFL | MR Egger | 74 | 0.109 (-0.222, 0.440) | 0.521 | 26 | 0.179 (-0.696, 1.053) | 0.692 | 21 | -0.064 (-0.147, 0.019) | 0.148 |
| mRNFL | Weighted median | 74 | -0.008 (-0.172, 0.155) | 0.919 | 26 | 0.224 (-0.321, 0.768) | 0.421 | 21 | -0.058 (-0.107, -0.010) | 0.018 |
| mRNFL | Weighted mode | 74 | -0.001 (-0.238, 0.236) | 0.995 | 26 | 0.161 (-0.398, 0.719) | 0.578 | 21 | -0.062 (-0.110, -0.014) | 0.019 |
| mGCIPL | Inverse variance weighted | 74 | 0.073 (-0.095, 0.242) | 0.394 | 26 | 0.442 (-0.159, 1.043) | 0.149 | 21 | -0.053 (-0.155, 0.049) | 0.309 |
| mGCIPL | MR Egger | 74 | 0.242 (-0.220, 0.704) | 0.308 | 26 | 0.170 (-0.694, 1.035) | 0.702 | 21 | -0.103 (-0.221, 0.015) | 0.102 |
| mGCIPL | Weighted median | 74 | 0.062 (-0.154, 0.278) | 0.574 | 26 | 0.284 (-0.398, 0.966) | 0.414 | 21 | -0.083 (-0.146, -0.019) | 0.011 |
| mGCIPL | Weighted mode | 74 | 0.040 (-0.299, 0.380) | 0.817 | 26 | 0.302 (-0.406, 1.009) | 0.411 | 21 | -0.081 (-0.146, -0.016) | 0.024 |
| MT | Inverse variance weighted | 59 | 0.076 (-0.221, 0.373) | 0.616 | 22 | 0.078 (-0.964, 1.120) | 0.883 | 13 | -0.005 (-0.251, 0.242) | 0.970 |
| MT | MR Egger | 59 | 0.059 (-0.465, 0.583) | 0.826 | 22 | -0.601 (-2.039, 0.837) | 0.422 | 13 | 0.166 (-0.302, 0.635) | 0.501 |
| MT | Weighted median | 59 | 0.093 (-0.143, 0.330) | 0.439 | 22 | -0.259 (-1.361, 0.843) | 0.645 | 13 | 0.063 (-0.205, 0.331) | 0.645 |
| MT | Weighted mode | 59 | 0.020 (-0.226, 0.267) | 0.871 | 22 | -0.211 (-1.291, 0.870) | 0.706 | 13 | 0.054 (-0.248, 0.357) | 0.731 |
| ONL | Inverse variance weighted | 74 | -0.001 (-0.200, 0.198) | 0.992 | 26 | 0.110 (-0.488, 0.708) | 0.718 | 21 | -0.043 (-0.141, 0.055) | 0.385 |
| ONL | MR Egger | 74 | 0.010 (-0.536, 0.556) | 0.972 | 26 | 0.080 (-0.775, 0.935) | 0.856 | 21 | -0.051 (-0.171, 0.069) | 0.415 |
| ONL | Weighted median | 74 | -0.028 (-0.247, 0.191) | 0.802 | 26 | 0.138 (-0.644, 0.920) | 0.729 | 21 | -0.053 (-0.121, 0.016) | 0.132 |
| ONL | Weighted mode | 74 | -0.168 (-0.480, 0.143) | 0.293 | 26 | 0.119 (-0.683, 0.921) | 0.774 | 21 | -0.056 (-0.129, 0.017) | 0.150 |
| IS | Inverse variance weighted | 74 | 0.007 (-0.035, 0.050) | 0.737 | 26 | 0.049 (-0.115, 0.214) | 0.558 | 21 | 0.005 (-0.016, 0.025) | 0.649 |
| IS | MR Egger | 74 | 0.044 (-0.072, 0.161) | 0.458 | 26 | -0.101 (-0.326, 0.124) | 0.386 | 21 | -0.002 (-0.027, 0.022) | 0.854 |
| IS | Weighted median | 74 | 0.010 (-0.043, 0.063) | 0.704 | 26 | -0.040 (-0.216, 0.137) | 0.661 | 21 | 0.000 (-0.016, 0.016) | 0.966 |
| IS | Weighted mode | 74 | 0.056 (-0.031, 0.142) | 0.212 | 26 | -0.046 (-0.228, 0.136) | 0.624 | 21 | -0.001 (-0.018, 0.016) | 0.907 |
| OS | Inverse variance weighted | 74 | 0.062 (-0.045, 0.170) | 0.256 | 26 | -0.176 (-0.719, 0.367) | 0.526 | 21 | 0.042 (-0.001, 0.086) | 0.056 |
| OS | MR Egger | 74 | -0.154 (-0.445, 0.137) | 0.303 | 26 | 0.039 (-0.745, 0.824) | 0.923 | 21 | 0.047 (-0.006, 0.100) | 0.096 |
| OS | Weighted median | 74 | -0.007 (-0.144, 0.130) | 0.920 | 26 | 0.033 (-0.440, 0.505) | 0.892 | 21 | 0.048 (0.004, 0.091) | 0.032 |
| OS | Weighted mode | 74 | -0.042 (-0.229, 0.145) | 0.661 | 26 | -0.031 (-0.501, 0.440) | 0.900 | 21 | 0.043 (0.001, 0.085) | 0.056 |
| AT | Inverse variance weighted | 80 | 0.002 (-0.009, 0.013) | 0.667 | 29 | 0.023 (-0.026, 0.071) | 0.365 | 21 | 0.006 (0.002, 0.010) | 0.008 |
| AT | MR Egger | 80 | 0.014 (-0.012, 0.041) | 0.283 | 29 | 0.004 (-0.065, 0.073) | 0.915 | 21 | 0.005 (0.000, 0.011) | 0.079 |
| AT | Weighted median | 80 | 0.004 (-0.010, 0.017) | 0.591 | 29 | 0.009 (-0.035, 0.054) | 0.677 | 21 | 0.006 (0.002, 0.010) | 0.006 |
| AT | Weighted mode | 80 | 0.014 (-0.006, 0.034) | 0.170 | 29 | 0.009 (-0.038, 0.056) | 0.710 | 21 | 0.006 (0.002, 0.010) | 0.009 |
| VT | Inverse variance weighted | 80 | -0.004 (-0.010, 0.002) | 0.170 | 29 | 0.006 (-0.022, 0.035) | 0.668 | 21 | 0.001 (-0.001, 0.004) | 0.338 |
| VT | MR Egger | 80 | -0.003 (-0.017, 0.010) | 0.619 | 29 | 0.017 (-0.023, 0.058) | 0.401 | 21 | 0.001 (-0.002, 0.005) | 0.445 |
| VT | Weighted median | 80 | -0.006 (-0.014, 0.002) | 0.137 | 29 | 0.020 (-0.007, 0.048) | 0.152 | 21 | 0.002 (-0.001, 0.004) | 0.132 |
| VT | Weighted mode | 80 | -0.008 (-0.021, 0.005) | 0.218 | 29 | 0.018 (-0.011, 0.047) | 0.232 | 21 | 0.002 (-0.001, 0.005) | 0.170 |
| VW | Inverse variance weighted | 80 | 0.084 (-0.158, 0.326) | 0.498 | 29 | 0.248 (-0.689, 1.185) | 0.604 | 21 | 0.000 (-0.125, 0.125) | 0.998 |
| VW | MR Egger | 80 | 0.461 (-0.111, 1.034) | 0.118 | 29 | -0.112 (-1.438, 1.213) | 0.869 | 21 | 0.040 (-0.111, 0.190) | 0.611 |
| VW | Weighted median | 80 | 0.179 (-0.149, 0.507) | 0.285 | 29 | -0.014 (-1.215, 1.188) | 0.982 | 21 | 0.014 (-0.093, 0.120) | 0.800 |
| VW | Weighted mode | 80 | 0.226 (-0.248, 0.700) | 0.353 | 29 | 0.126 (-1.104, 1.356) | 0.843 | 21 | 0.014 (-0.091, 0.120) | 0.791 |
| FD | Inverse variance weighted | 67 | 0.000 (-0.001, 0.000) | 0.431 | 23 | 0.000 (-0.002, 0.003) | 0.812 | 19 | 0.000 (0.000, 0.000) | 0.121 |
| FD | MR Egger | 67 | -0.001 (-0.002, 0.001) | 0.513 | 23 | -0.002 (-0.005, 0.002) | 0.327 | 19 | 0.000 (0.000, 0.000) | 0.213 |
| FD | Weighted median | 67 | 0.000 (-0.001, 0.001) | 0.605 | 23 | -0.001 (-0.004, 0.002) | 0.508 | 19 | 0.000 (0.000, 0.000) | 0.136 |
| FD | Weighted mode | 67 | 0.000 (-0.002, 0.002) | 0.833 | 23 | -0.001 (-0.004, 0.002) | 0.477 | 19 | 0.000 (0.000, 0.000) | 0.147 |
| ODA | Inverse variance weighted | 73 | -0.020 (-0.035, -0.005) | 0.010 | 23 | -0.098 (-0.186, -0.009) | 0.030 | 21 | -0.007 (-0.016, 0.002) | 0.117 |
| ODA | MR Egger | 73 | -0.041 (-0.082, 0.000) | 0.056 | 23 | -0.045 (-0.173, 0.083) | 0.497 | 21 | -0.006 (-0.017, 0.005) | 0.308 |
| ODA | Weighted median | 73 | -0.032 (-0.055, -0.010) | 0.005 | 23 | -0.037 (-0.119, 0.044) | 0.372 | 21 | -0.006 (-0.013, 0.002) | 0.128 |
| ODA | Weighted mode | 73 | -0.035 (-0.065, -0.006) | 0.020 | 23 | -0.073 (-0.153, 0.008) | 0.090 | 21 | -0.006 (-0.013, 0.001) | 0.123 |
| OCA | Inverse variance weighted | 73 | 0.001 (-0.006, 0.009) | 0.740 | 23 | -0.007 (-0.042, 0.028) | 0.694 | 21 | 0.001 (-0.003, 0.004) | 0.776 |
| OCA | MR Egger | 73 | 0.002 (-0.019, 0.023) | 0.853 | 23 | 0.008 (-0.042, 0.059) | 0.748 | 21 | 0.001 (-0.004, 0.005) | 0.704 |
| OCA | Weighted median | 73 | -0.003 (-0.015, 0.009) | 0.653 | 23 | 0.000 (-0.043, 0.044) | 0.989 | 21 | 0.000 (-0.004, 0.004) | 0.859 |
| OCA | Weighted mode | 73 | 0.007 (-0.010, 0.024) | 0.442 | 23 | 0.005 (-0.041, 0.050) | 0.841 | 21 | 0.001 (-0.003, 0.005) | 0.732 |
| VCDR | Inverse variance weighted | 73 | -0.002 (-0.006, 0.002) | 0.414 | 23 | -0.013 (-0.034, 0.008) | 0.238 | 21 | -0.001 (-0.003, 0.001) | 0.274 |
| VCDR | MR Egger | 73 | -0.005 (-0.017, 0.007) | 0.429 | 23 | -0.008 (-0.041, 0.024) | 0.624 | 21 | 0.000 (-0.003, 0.002) | 0.808 |
| VCDR | Weighted median | 73 | -0.003 (-0.010, 0.003) | 0.321 | 23 | -0.010 (-0.034, 0.014) | 0.420 | 21 | -0.001 (-0.003, 0.002) | 0.587 |
| VCDR | Weighted mode | 73 | -0.004 (-0.013, 0.005) | 0.380 | 23 | -0.010 (-0.035, 0.014) | 0.424 | 21 | -0.001 (-0.003, 0.001) | 0.510 |

**Abbreviations:** AD= Alzheimer’s disease, RNFL=Retinal Nerve Fibre Layer, GCIPL=Ganglion Cell Inner Plexiform Layer, ONL=outer nuclear layer, IS=inner segment layer thickness, OS=outer segment layer, AT=Arteriolar Tortuosity, VT=Venular Tortuosity, VW=Venular Width, FD=retinal vessel fractal dimension ODA=optic disc area, OCA=optic cup area, *and* VCDR=vertical cup disc ratio*.* The estimates (β) are given for SD change per doubling odds of Alzheimer’s disease genetic risk.

## **Table S27. The causal effects of ocular features on Alzheimer's disease based on univariable Mendelian randomization for other AD GWASs.**

| **Exposure** | **MR methods** | **AD GWAS: Bellenguez et al (2022)** | | | **AD GWAS: Jansen et al (2019)** | | | **AD GWAS: Lambert et al (2013)** | | |
| --- | --- | --- | --- | --- | --- | --- | --- | --- | --- | --- |
|  |  | **SNPs** | **OR (95%CI)** | **P-value** | **SNPs** | **OR (95%CI)** | **P-value** | **SNPs** | **OR (95%CI)** | **P-value** |
| mRNFL | MR Egger | 22 | 1.027 (0.989, 1.066) | 0.19 | 25 | 0.995 (0.981, 1.009) | 0.48 | 21 | 1.028 (0.945, 1.117) | 0.53 |
| mRNFL | Inverse variance weighted | 22 | 1.003 (0.990, 1.017) | 0.63 | 25 | 1.000 (0.996, 1.005) | 0.97 | 21 | 1.007 (0.981, 1.033) | 0.60 |
| mRNFL | Weighted median | 22 | 1.010 (0.991, 1.028) | 0.30 | 25 | 1.001 (0.995, 1.007) | 0.84 | 21 | 1.014 (0.977, 1.052) | 0.47 |
| mRNFL | Weighted mode | 22 | 1.012 (0.985, 1.041) | 0.39 | 25 | 1.001 (0.993, 1.010) | 0.77 | 21 | 1.020 (0.965, 1.078) | 0.49 |
| mGCIPL | MR Egger | 21 | 1.026 (0.994, 1.059) | 0.13 | 21 | 1.002 (0.994, 1.010) | 0.60 | 18 | 1.012 (0.954, 1.073) | 0.70 |
| mGCIPL | Inverse variance weighted | 21 | 1.006 (0.995, 1.018) | 0.29 | 21 | 1.004 (1.001, 1.007) | 0.01 | 18 | 1.026 (1.004, 1.049) | 0.02 |
| mGCIPL | Weighted median | 21 | 1.009 (0.995, 1.023) | 0.20 | 21 | 1.004 (0.999, 1.008) | 0.09 | 18 | 1.015 (0.983, 1.047) | 0.37 |
| mGCIPL | Weighted mode | 21 | 1.007 (0.984, 1.030) | 0.56 | 21 | 1.002 (0.995, 1.008) | 0.59 | 18 | 1.011 (0.967, 1.058) | 0.63 |
| MT | MR Egger | 106 | 1.000 (0.991, 1.009) | 0.96 | 107 | 1.000 (0.997, 1.002) | 0.91 | 87 | 0.995 (0.977, 1.013) | 0.59 |
| MT | Inverse variance weighted | 106 | 0.999 (0.995, 1.002) | 0.48 | 107 | 1.000 (0.999, 1.001) | 0.37 | 87 | 1.000 (0.992, 1.007) | 0.93 |
| MT | Weighted median | 106 | 1.000 (0.996, 1.005) | 0.90 | 107 | 1.000 (0.998, 1.001) | 0.80 | 87 | 1.001 (0.991, 1.010) | 0.87 |
| MT | Weighted mode | 106 | 1.000 (0.994, 1.005) | 0.95 | 107 | 0.999 (0.998, 1.001) | 0.47 | 87 | 1.000 (0.988, 1.011) | 0.94 |
| ONL | Inverse variance weighted | 61 | 0.999 (0.992, 1.005) | 0.70 | 61 | 1.000 (0.999, 1.002) | 0.68 | 46 | 0.998 (0.986, 1.010) | 0.71 |
| ONL | MR Egger | 61 | 0.999 (0.986, 1.012) | 0.85 | 61 | 1.000 (0.997, 1.002) | 0.76 | 46 | 0.993 (0.970, 1.017) | 0.57 |
| ONL | Weighted median | 61 | 0.997 (0.989, 1.005) | 0.46 | 61 | 1.000 (0.997, 1.002) | 0.76 | 46 | 0.994 (0.977, 1.011) | 0.47 |
| ONL | Weighted mode | 61 | 0.996 (0.988, 1.004) | 0.37 | 61 | 0.999 (0.997, 1.002) | 0.62 | 46 | 0.992 (0.973, 1.011) | 0.40 |
| IS | Inverse variance weighted | 15 | 0.958 (0.902, 1.017) | 0.16 | 15 | 0.995 (0.976, 1.014) | 0.61 | 14 | 0.928 (0.818, 1.052) | 0.24 |
| IS | MR Egger | 15 | 0.939 (0.757, 1.164) | 0.57 | 15 | 0.961 (0.901, 1.026) | 0.25 | 14 | 0.856 (0.554, 1.322) | 0.50 |
| IS | Weighted median | 15 | 0.962 (0.889, 1.040) | 0.33 | 15 | 0.989 (0.968, 1.010) | 0.31 | 14 | 0.911 (0.780, 1.064) | 0.24 |
| IS | Weighted mode | 15 | 0.958 (0.866, 1.061) | 0.43 | 15 | 0.986 (0.960, 1.012) | 0.30 | 14 | 0.926 (0.763, 1.125) | 0.45 |
| OS | Inverse variance weighted | 35 | 1.004 (0.990, 1.017) | 0.60 | 35 | 1.002 (0.999, 1.006) | 0.21 | 30 | 0.996 (0.973, 1.020) | 0.76 |
| OS | MR Egger | 35 | 0.995 (0.957, 1.035) | 0.81 | 35 | 1.002 (0.991, 1.013) | 0.77 | 30 | 1.054 (0.981, 1.133) | 0.16 |
| OS | Weighted median | 35 | 0.992 (0.976, 1.008) | 0.30 | 35 | 1.003 (0.998, 1.007) | 0.23 | 30 | 0.999 (0.968, 1.031) | 0.95 |
| OS | Weighted mode | 35 | 0.982 (0.959, 1.006) | 0.16 | 35 | 0.999 (0.992, 1.006) | 0.79 | 30 | 0.999 (0.949, 1.051) | 0.97 |
| AT | Inverse variance weighted | 83 | 0.945 (0.874, 1.021) | 0.15 | 84 | 1.004 (0.981, 1.027) | 0.73 | 73 | 0.936 (0.786, 1.114) | 0.45 |
| AT | MR Egger | 83 | 1.183 (0.954, 1.468) | 0.13 | 84 | 1.078 (1.010, 1.151) | 0.03 | 73 | 1.311 (0.784, 2.194) | 0.31 |
| AT | Weighted median | 83 | 0.930 (0.837, 1.033) | 0.18 | 84 | 1.016 (0.983, 1.050) | 0.34 | 73 | 1.017 (0.803, 1.288) | 0.89 |
| AT | Weighted mode | 83 | 0.912 (0.756, 1.099) | 0.34 | 84 | 1.034 (0.970, 1.103) | 0.31 | 73 | 1.459 (0.925, 2.301) | 0.11 |
| VT | Inverse variance weighted | 14 | 1.315 (0.980, 1.765) | 0.07 | 16 | 1.081 (1.006, 1.162) | 0.03 | 14 | 1.004 (0.567, 1.776) | 0.99 |
| VT | MR Egger | 14 | 1.202 (0.620, 2.332) | 0.60 | 16 | 1.203 (1.038, 1.394) | 0.03 | 14 | 1.392 (0.399, 4.863) | 0.61 |
| VT | Weighted median | 14 | 1.275 (0.908, 1.790) | 0.16 | 16 | 1.139 (1.047, 1.238) | 0.00 | 14 | 1.358 (0.706, 2.610) | 0.36 |
| VT | Weighted mode | 14 | 1.237 (0.847, 1.806) | 0.29 | 16 | 1.131 (1.041, 1.229) | 0.01 | 14 | 1.511 (0.758, 3.012) | 0.26 |
| VW | MR Egger | 13 | 1.028 (0.986, 1.071) | 0.22 | 13 | 1.013 (0.994, 1.031) | 0.20 | 11 | 1.047 (0.942, 1.164) | 0.41 |
| VW | Inverse variance weighted | 13 | 1.000 (0.991, 1.009) | 0.99 | 13 | 1.001 (0.997, 1.005) | 0.49 | 11 | 1.005 (0.985, 1.026) | 0.60 |
| VW | Weighted median | 13 | 1.002 (0.990, 1.014) | 0.77 | 13 | 1.001 (0.997, 1.004) | 0.60 | 11 | 1.001 (0.976, 1.028) | 0.92 |
| VW | Weighted mode | 13 | 1.006 (0.986, 1.026) | 0.59 | 13 | 1.001 (0.995, 1.007) | 0.77 | 11 | 1.006 (0.958, 1.055) | 0.83 |
| FD | Inverse variance weighted | 2 | 0.021 (0.000, 13.415) | 0.24 | 3 | 0.903 (0.257, 3.170) | 0.90 | 3 | 0.517 (0.000, 41089.732) | 0.93 |
| - | - | - | - | - | 3 | 0.862 (0.457, 1.627) | 0.65 | 3 | 0.808 (0.004, 185.345) | 0.94 |
| - | - | - | - | - | 3 | 0.866 (0.442, 1.696) | 0.68 | 3 | 0.771 (0.003, 172.090) | 0.92 |
| - | - | - | - | - | 3 | 0.837 (0.421, 1.664) | 0.66 | 3 | 0.825 (0.003, 241.860) | 0.95 |
| ODA | Inverse variance weighted | 11 | 0.980 (0.875, 1.099) | 0.73 | 11 | 0.968 (0.939, 0.999) | 0.04 | 11 | 0.780 (0.625, 0.972) | 0.03 |
| ODA | MR Egger | 11 | 0.778 (0.619, 0.977) | 0.06 | 11 | 0.931 (0.874, 0.991) | 0.05 | 11 | 0.768 (0.494, 1.196) | 0.27 |
| ODA | Weighted median | 11 | 0.890 (0.768, 1.031) | 0.12 | 11 | 0.955 (0.921, 0.991) | 0.02 | 11 | 0.875 (0.651, 1.176) | 0.38 |
| ODA | Weighted mode | 11 | 0.894 (0.773, 1.033) | 0.16 | 11 | 0.953 (0.919, 0.988) | 0.03 | 11 | 0.910 (0.634, 1.305) | 0.62 |
| OCA | MR Egger | 39 | 1.050 (1.009, 1.092) | 0.02 | 40 | 1.008 (0.994, 1.022) | 0.27 | 12 | 4.171 (0.055, 318.592) | 0.53 |
| OCA | Inverse variance weighted | 39 | 1.046 (1.009, 1.083) | 0.01 | 40 | 1.009 (0.996, 1.022) | 0.16 | 12 | 0.979 (0.492, 1.949) | 0.95 |
| OCA | Weighted median | 39 | 1.051 (0.997, 1.109) | 0.06 | 40 | 1.005 (0.989, 1.022) | 0.51 | 12 | 0.826 (0.395, 1.728) | 0.61 |
| OCA | Weighted mode | 39 | 1.039 (0.987, 1.094) | 0.15 | 40 | 1.006 (0.993, 1.019) | 0.36 | 12 | 0.769 (0.207, 2.854) | 0.70 |
| VCDR | Inverse variance weighted | 17 | 0.819 (0.490, 1.369) | 0.45 | 17 | 0.935 (0.837, 1.043) | 0.23 | 16 | 0.540 (0.198, 1.470) | 0.23 |
| VCDR | MR Egger | 17 | 0.783 (0.304, 2.017) | 0.62 | 17 | 0.892 (0.725, 1.096) | 0.29 | 16 | 0.343 (0.016, 7.396) | 0.51 |
| VCDR | Weighted median | 17 | 0.923 (0.544, 1.568) | 0.77 | 17 | 0.939 (0.802, 1.099) | 0.43 | 16 | 0.625 (0.186, 2.093) | 0.45 |
| VCDR | Weighted mode | 17 | 0.840 (0.478, 1.474) | 0.55 | 17 | 0.868 (0.708, 1.064) | 0.19 | 16 | 0.572 (0.107, 3.044) | 0.52 |

**Abbreviations:** AD= Alzheimer’s disease, RNFL=Retinal Nerve Fibre Layer, GCIPL=Ganglion Cell Inner Plexiform Layer, ONL=outer nuclear layer, IS=inner segment layer thickness, OS=outer segment layer, AT=Arteriolar Tortuosity, VT=Venular Tortuosity, VW=Venular Width, FD=retinal vessel fractal dimension ODA=optic disc area, OCA=optic cup area, *and* VCDR=vertical cup disc ratio*.* Causal estimates are odds ratios for Alzheimer’s Disease risk

## **Table S28. Genetic correlations between ocular traits or Alzheimer’s disease.**

| **Trait 1** | **Trait 2** | **Correlation** | **Standard error** | **Z** | **P-value** |
| --- | --- | --- | --- | --- | --- |
| AD | RNFL | -0.08 | 0.08 | -0.96 | 0.34 |
| AD | GCIPL | -0.05 | 0.08 | -0.68 | 0.50 |
| AD | ONL | -0.04 | 0.07 | -0.51 | 0.61 |
| AD | IS | 0.00 | 0.09 | -0.05 | 0.96 |
| AD | OS | -0.04 | 0.08 | -0.54 | 0.59 |
| AD | AT | -0.03 | 0.06 | -0.41 | 0.68 |
| AD | VT | 0.06 | 0.08 | 0.79 | 0.43 |
| AD | VW | -0.07 | 0.09 | -0.81 | 0.42 |
| AD | FD | 0.08 | 0.08 | 0.95 | 0.34 |
| AD | ODA | -0.14 | 0.09 | -1.57 | 0.12 |
| AD | OCA | -0.05 | 0.08 | -0.59 | 0.55 |
| AD | VCDR | -0.11 | 0.07 | -1.47 | 0.14 |
| AD | AL | -0.26 | 0.11 | -2.32 | **0.02** |
| AD | RE | 0.12 | 0.05 | 2.57 | **0.01** |
| RNFL | GCIPL | 0.51 | 0.05 | 10.96 | **0.00** |
| RNFL | ONL | -0.13 | 0.11 | -1.15 | 0.25 |
| RNFL | IS | -0.11 | 0.08 | -1.30 | 0.19 |
| RNFL | OS | -0.01 | 0.08 | -0.13 | 0.89 |
| RNFL | AT | -0.11 | 0.04 | -2.73 | **0.01** |
| RNFL | VT | -0.01 | 0.06 | -0.09 | 0.93 |
| RNFL | VW | -0.19 | 0.07 | -2.56 | **0.01** |
| RNFL | FD | 0.07 | 0.06 | 1.15 | 0.25 |
| RNFL | ODA | 0.07 | 0.13 | 0.50 | 0.62 |
| RNFL | OCA | 0.07 | 0.08 | 0.93 | 0.35 |
| RNFL | VCDR | 0.07 | 0.08 | 0.81 | 0.42 |
| RNFL | AL | 0.16 | 0.06 | 2.61 | **0.01** |
| RNFL | RE | 0.00 | 0.04 | 0.08 | 0.94 |
| GCIPL | ONL | 0.16 | 0.09 | 1.79 | 0.07 |
| GCIPL | IS | 0.14 | 0.07 | 2.03 | 0.04 |
| GCIPL | OS | -0.03 | 0.06 | -0.44 | 0.66 |
| GCIPL | AT | 0.05 | 0.04 | 1.10 | 0.27 |
| GCIPL | VT | 0.14 | 0.06 | 2.55 | **0.01** |
| GCIPL | VW | 0.10 | 0.06 | 1.53 | 0.13 |
| GCIPL | FD | 0.03 | 0.06 | 0.44 | 0.66 |
| GCIPL | ODA | 0.04 | 0.06 | 0.66 | 0.51 |
| GCIPL | OCA | -0.03 | 0.06 | -0.51 | 0.61 |
| GCIPL | VCDR | -0.01 | 0.05 | -0.11 | 0.92 |
| GCIPL | AL | -0.07 | 0.06 | -1.25 | 0.21 |
| GCIPL | RE | 0.07 | 0.03 | 2.25 | **0.02** |
| ONL | IS | 0.55 | 0.06 | 8.62 | **0.00** |
| ONL | OS | 0.09 | 0.05 | 1.63 | 0.10 |
| ONL | AT | 0.02 | 0.04 | 0.59 | 0.56 |
| ONL | VT | 0.07 | 0.06 | 1.32 | 0.19 |
| ONL | VW | 0.30 | 0.06 | 5.31 | **0.00** |
| ONL | FD | 0.07 | 0.05 | 1.41 | 0.16 |
| ONL | ODA | -0.09 | 0.06 | -1.55 | 0.12 |
| ONL | OCA | -0.09 | 0.05 | -1.80 | 0.07 |
| ONL | VCDR | -0.05 | 0.05 | -0.97 | 0.33 |
| ONL | AL | -0.07 | 0.06 | -1.07 | 0.29 |
| ONL | RE | 0.03 | 0.03 | 0.92 | 0.36 |
| IS | OS | -0.07 | 0.08 | -0.91 | 0.36 |
| IS | AT | 0.00 | 0.05 | 0.03 | 0.98 |
| IS | VT | 0.01 | 0.07 | 0.14 | 0.89 |
| IS | VW | 0.40 | 0.08 | 5.14 | **0.00** |
| IS | FD | 0.16 | 0.06 | 2.54 | **0.01** |
| IS | ODA | 0.04 | 0.07 | 0.56 | 0.58 |
| IS | OCA | -0.09 | 0.08 | -1.21 | 0.23 |
| IS | VCDR | -0.01 | 0.07 | -0.13 | 0.90 |
| IS | AL | -0.12 | 0.08 | -1.49 | 0.14 |
| IS | RE | 0.09 | 0.04 | 2.48 | **0.01** |
| OS | AT | -0.09 | 0.05 | -1.94 | 0.05 |
| OS | VT | -0.11 | 0.06 | -1.70 | 0.09 |
| OS | VW | 0.05 | 0.07 | 0.72 | 0.47 |
| OS | FD | -0.04 | 0.06 | -0.63 | 0.53 |
| OS | ODA | -0.04 | 0.07 | -0.61 | 0.54 |
| OS | OCA | 0.06 | 0.07 | 0.85 | 0.40 |
| OS | VCDR | 0.00 | 0.06 | -0.02 | 0.98 |
| OS | AL | -0.10 | 0.06 | -1.55 | 0.12 |
| OS | RE | -0.01 | 0.03 | -0.43 | 0.67 |
| AT | VT | 0.37 | 0.06 | 6.00 | **0.00** |
| AT | VW | 0.08 | 0.05 | 1.48 | 0.14 |
| AT | FD | -0.10 | 0.04 | -2.32 | **0.02** |
| AT | ODA | -0.04 | 0.04 | -0.93 | 0.35 |
| AT | OCA | -0.05 | 0.04 | -1.22 | 0.22 |
| AT | VCDR | -0.05 | 0.04 | -1.29 | 0.20 |
| AT | AL | -0.11 | 0.05 | -2.32 | **0.02** |
| AT | RE | 0.01 | 0.03 | 0.49 | 0.63 |
| VT | VW | -0.20 | 0.07 | -2.83 | **0.00** |
| VT | FD | 0.09 | 0.06 | 1.56 | 0.12 |
| VT | ODA | -0.23 | 0.06 | -4.01 | **0.00** |
| VT | OCA | -0.14 | 0.06 | -2.45 | **0.01** |
| VT | VCDR | -0.21 | 0.05 | -4.07 | **0.00** |
| VT | AL | 0.00 | 0.06 | -0.02 | 0.98 |
| VT | RE | 0.01 | 0.03 | 0.40 | 0.69 |
| VW | FD | -0.51 | 0.10 | -5.06 | **0.00** |
| VW | ODA | 0.01 | 0.09 | 0.07 | 0.94 |
| VW | OCA | 0.06 | 0.07 | 0.88 | 0.38 |
| VW | VCDR | 0.08 | 0.07 | 1.14 | 0.25 |
| VW | AL | -0.07 | 0.08 | -0.89 | 0.37 |
| VW | RE | 0.15 | 0.04 | 4.14 | **0.00** |
| FD | ODA | -0.07 | 0.06 | -1.25 | 0.21 |
| FD | OCA | -0.03 | 0.06 | -0.47 | 0.64 |
| FD | VCDR | 0.00 | 0.05 | 0.01 | 0.99 |
| FD | AL | -0.13 | 0.06 | -2.01 | 0.04 |
| FD | RE | 0.04 | 0.03 | 1.27 | 0.20 |
| ODA | OCA | 0.30 | 0.08 | 3.84 | **0.00** |
| ODA | VCDR | 0.63 | 0.06 | 10.82 | **0.00** |
| ODA | AL | 0.07 | 0.06 | 1.27 | 0.20 |
| ODA | RE | -0.07 | 0.03 | -2.17 | 0.03 |
| OCA | VCDR | 0.90 | 0.02 | 52.98 | **0.00** |
| OCA | AL | 0.04 | 0.07 | 0.54 | 0.59 |
| OCA | RE | 0.02 | 0.04 | 0.42 | 0.67 |
| VCDR | AL | 0.10 | 0.06 | 1.62 | 0.11 |
| VCDR | RE | -0.02 | 0.03 | -0.73 | 0.47 |
| AL | RE | -0.81 | 0.04 | -21.47 | **0.00** |

**Abbreviations:** AD= Alzheimer’s disease, RNFL=Retinal Nerve Fibre Layer, GCIPL=Ganglion Cell Inner Plexiform Layer, ONL=outer nuclear layer, IS=inner segment layer thickness, OS=outer segment layer, ODA=optic disc area, OCA=optic cup area, VCDR=vertical cup disc ratio, AT=Arteriolar Tortuosity, VT=Venular Tortuosity, VW=Venular Width, and FD=retinal vessel fractal dimension, AL=axial length, RE=refractive error.

# Supplementary References

[1] Davies NM, Holmes MV, Smith GD. Reading Mendelian randomisation studies: a guide, glossary, and checklist for clinicians. bmj. 2018;362. https://doi.org/10.1136/bmj.k601.

[2] Hemani G, Bowden J, Davey Smith G. Evaluating the potential role of pleiotropy in Mendelian randomization studies. Human molecular genetics. 2018;27(R2):R195-R208. https://doi.org/10.1093/hmg/ddy163.

[3] Skrivankova VW, Richmond RC, Woolf BA, Davies NM, Swanson SA, VanderWeele TJ, et al. Strengthening the reporting of observational studies in epidemiology using mendelian randomisation (STROBE-MR): explanation and elaboration. bmj. 2021;375. https://doi.org/10.1136/bmj.n2233.

[4] Sanderson E, Davey Smith G, Windmeijer F, Bowden J. An examination of multivariable Mendelian randomization in the single-sample and two-sample summary data settings. International journal of epidemiology. 2019;48(3):713-27. https://doi.org/10.1093/ije/dyy262.

[5] Burgess S, Thompson DJ, Rees JM, Day FR, Perry JR, Ong KK. Dissecting causal pathways using Mendelian randomization with summarized genetic data: application to age at menarche and risk of breast cancer. Genetics. 2017;207(2):481-7. https://doi.org/10.1534/genetics.117.300191.

[6] Elsworth B, Lyon M, Alexander T, Liu Y, Matthews P, Hallett J, et al. The MRC IEU OpenGWAS data infrastructure. BioRxiv. 2020:2020.08. 10.244293. https://doi.org/10.1101/2020.08.10.244293.

[7] Kunkle BW, Grenier-Boley B, Sims R, Bis JC, Damotte V, Naj AC, et al. Genetic meta-analysis of diagnosed Alzheimer’s disease identifies new risk loci and implicates Aβ, tau, immunity and lipid processing. Nature genetics. 2019;51(3):414-30. https://doi.org/10.1038/s41588-019-0358-2.

[8] Lambert J-C, Ibrahim-Verbaas CA, Harold D, Naj AC, Sims R, Bellenguez C, et al. Meta-analysis of 74,046 individuals identifies 11 new susceptibility loci for Alzheimer's disease. Nature genetics. 2013;45(12):1452-8. https://doi.org/10.1038/ng.2802.

[9] Biobank U. UK Biobank: protocol for a large-scale prospective epidemiological resource. Accessed May. 2007;7(2016):1-112.

[10] Keane PA, Grossi CM, Foster PJ, Yang Q, Reisman CA, Chan K, et al. Optical Coherence Tomography in the UK Biobank Study – Rapid Automated Analysis of Retinal Thickness for Large Population-Based Studies. PLoS One. 2016;11(10):e0164095. https://doi.org/10.1371/journal.pone.0164095.

[11] Yang Q, Reisman CA, Wang Z, Fukuma Y, Hangai M, Yoshimura N, et al. Automated layer segmentation of macular OCT images using dual-scale gradient information. Optics Express. 2010;18(20):21293. https://doi.org/10.1364/oe.18.021293.

[12] Gao XR, Huang H, Kim H. Genome-wide association analyses identify 139 loci associated with macular thickness in the UK Biobank cohort. Human molecular genetics. 2019;28(7):1162-72. https://doi.org/10.1093/hmg/ddy422.

[13] Currant H, Hysi P, Fitzgerald TW, Gharahkhani P, Bonnemaijer PWM, Senabouth A, et al. Genetic variation affects morphological retinal phenotypes extracted from UK Biobank optical coherence tomography images. PLOS Genetics. 2021;17(5):e1009497. https://doi.org/10.1371/journal.pgen.1009497.

[14] Currant H, Fitzgerald TW, Patel PJ, Khawaja AP, Eye UB, Consortium V, et al. Sub-cellular level resolution of common genetic variation in the photoreceptor layer identifies continuum between rare disease and common variation. PLoS Genetics. 2023;19(2):e1010587. https://doi.org/10.1371/journal.pgen.1010587.

[15] Jiang X, Hysi PG, Khawaja AP, Mahroo OA, Xu Z, Hammond CJ, et al. GWAS on retinal vasculometry phenotypes. PLOS Genetics. 2023;19(2):e1010583. https://doi.org/10.1371/journal.pgen.1010583.

[16] Villaplana-Velasco A, Pigeyre M, Engelmann J, Rawlik K, Canela-Xandri O, Tochel C, et al. Fine-mapping of retinal vascular complexity loci identifies Notch regulation as a shared mechanism with myocardial infarction outcomes. Communications biology. 2023;6(1):523. https://doi.org/10.1038/s42003-023-04836-9.

[17] Stosic T, Stosic BD. Multifractal analysis of human retinal vessels. IEEE Transactions on Medical Imaging. 2006;25(8):1101-7. https://doi.org/10.1109/tmi.2006.879316.

[18] Springelkamp H, Iglesias AI, Mishra A, Höhn R, Wojciechowski R, Khawaja AP, et al. New insights into the genetics of primary open-angle glaucoma based on meta-analyses of intraocular pressure and optic disc characteristics. Human molecular genetics. 2017;26(2):438-53. https://doi.org/10.1093/hmg/ddw399.

[19] Hysi PG, Choquet H, Khawaja AP, Wojciechowski R, Tedja MS, Yin J, et al. Meta-analysis of 542,934 subjects of European ancestry identifies new genes and mechanisms predisposing to refractive error and myopia. Nature genetics. 2020;52(4):401-7. https://doi.org/10.1038/s41588-020-0599-0.

[20] Jiang C, Melles RB, Yin J, Fan Q, Guo X, Cheng C-Y, et al. A multiethnic genome-wide analysis of 19,420 individuals identifies novel loci associated with axial length and shared genetic influences with refractive error and myopia. Frontiers in Genetics. 2023;14:1113058. https://doi.org/10.3389/fgene.2023.1113058.

[21] Sanderson E, Spiller W, Bowden J. Testing and correcting for weak and pleiotropic instruments in two‐sample multivariable Mendelian randomization. Statistics in medicine. 2021;40(25):5434-52. https://doi.org/10.1002/sim.9133.

[22] Jansen IE, Savage JE, Watanabe K, Bryois J, Williams DM, Steinberg S, et al. Genome-wide meta-analysis identifies new loci and functional pathways influencing Alzheimer’s disease risk. Nature genetics. 2019;51(3):404-13. https://doi.org/10.1038/s41588-018-0311-9.

[23] Bellenguez C, Küçükali F, Jansen IE, Kleineidam L, Moreno-Grau S, Amin N, et al. New insights into the genetic etiology of Alzheimer’s disease and related dementias. Nature genetics. 2022;54(4):412-36. https://doi.org/10.1038/s41588-022-01024-z.
